# Supplementary material for: The accuracy of diagnostic indicators for coeliac disease: A systematic review and meta-analysis
Source: PLoS One. 2021 Oct 25;16(10):e0258501. doi: 10.1371/journal.pone.0258501 (PMC8545431; doi:10.1371/journal.pone.0258501)

## Figure S1: Summary graph of risk of bias

Each of the bars represent 100% of the studies per indicator.

1. Symptoms


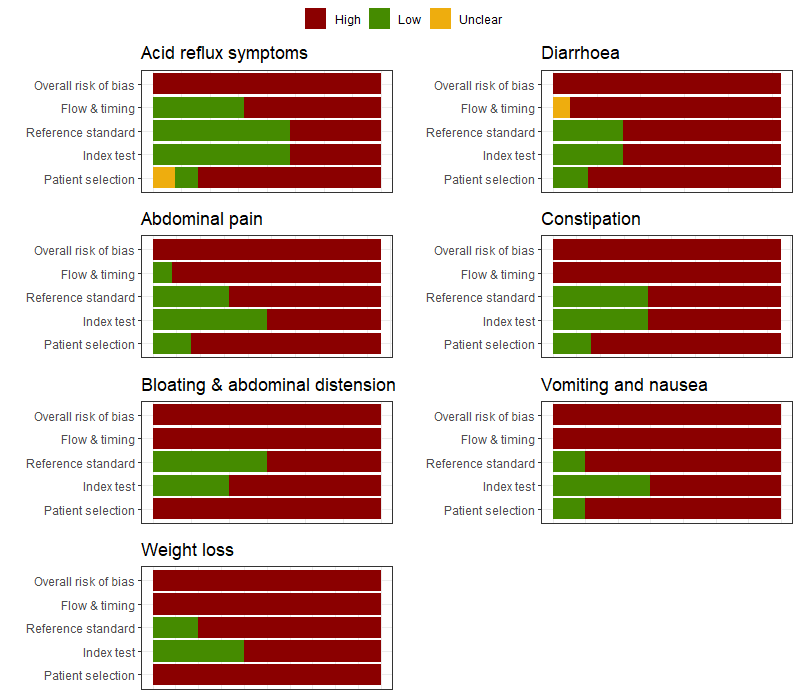


1. Risk conditions


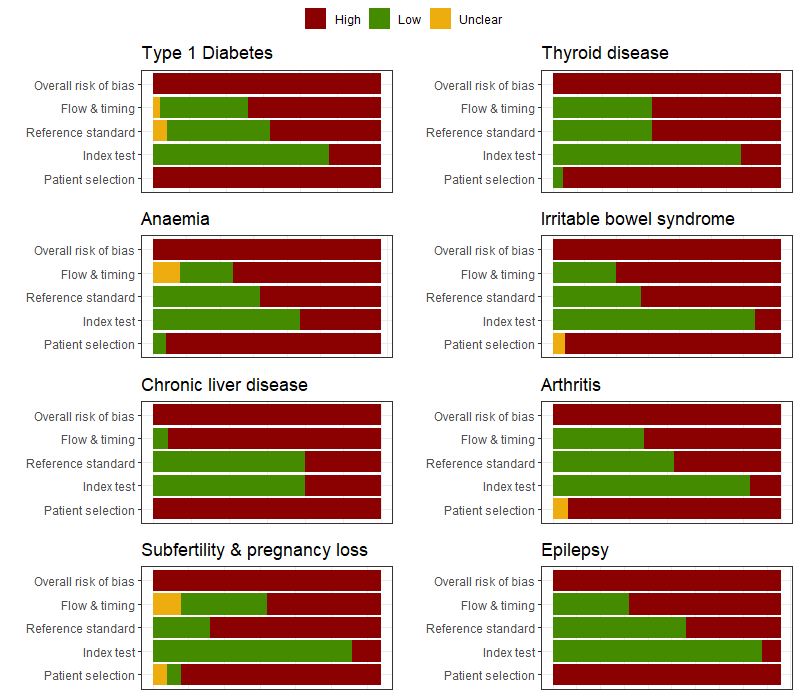


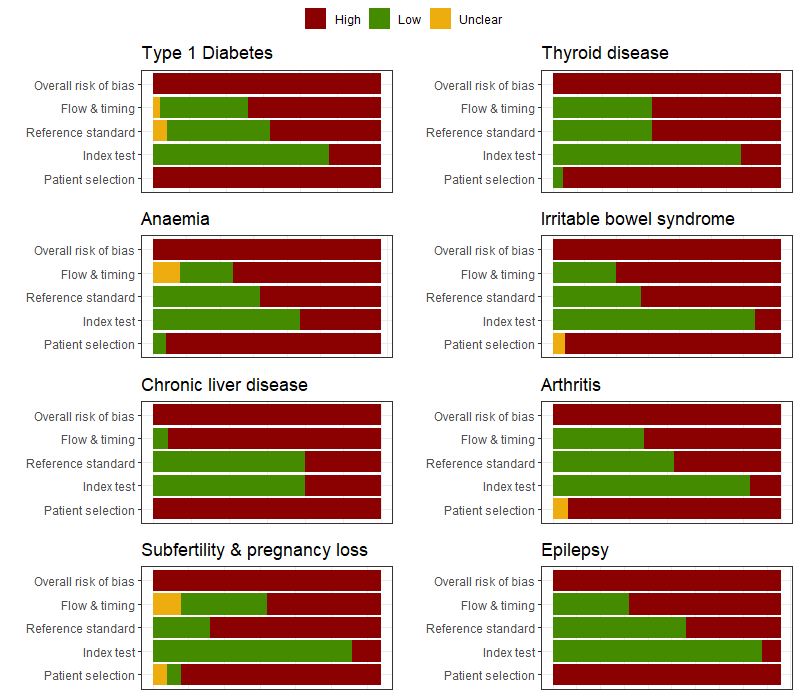


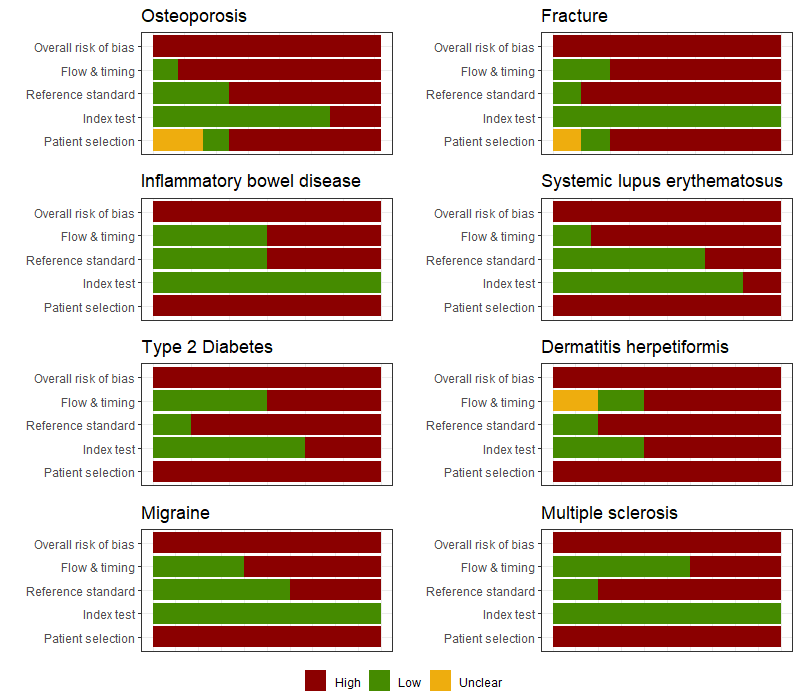


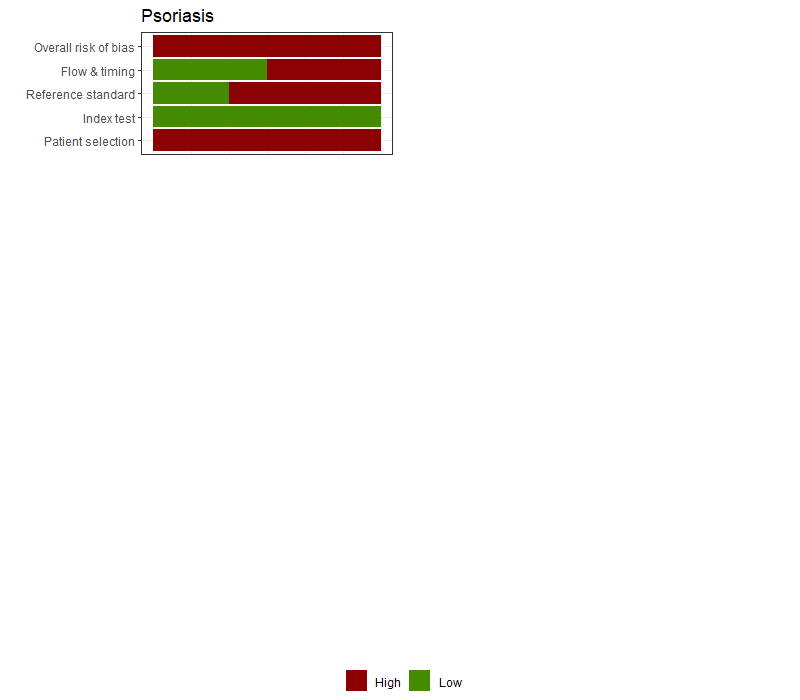


1. Genetic predisposition


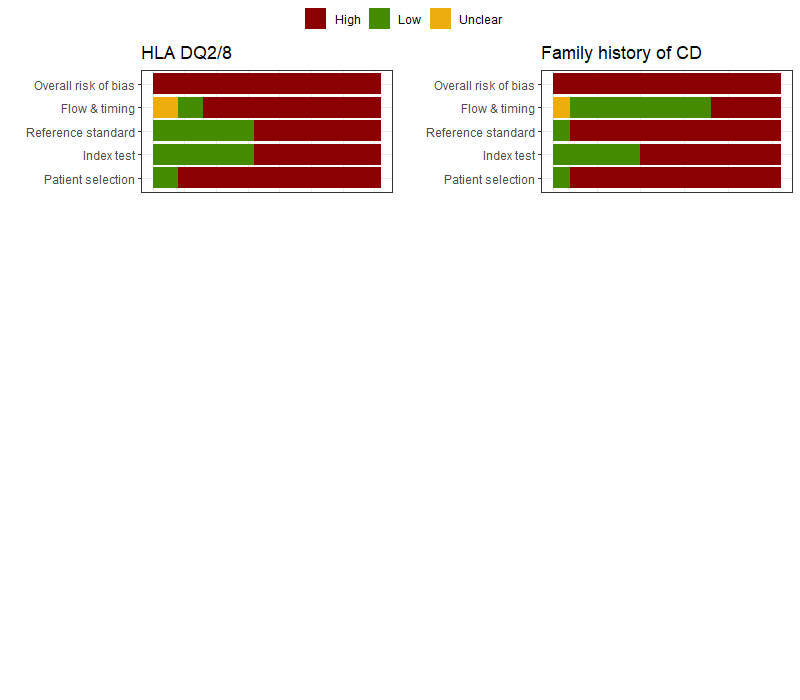

Supplement: S1 Fig — (DOCX) [file pone.0258501.s002.docx]
