# Supplementary material for: The accuracy of diagnostic indicators for coeliac disease: A systematic review and meta-analysis
Source: PLoS One. 2021 Oct 25;16(10):e0258501. doi: 10.1371/journal.pone.0258501 (PMC8545431; doi:10.1371/journal.pone.0258501)

## Figure S2: Forest plots of sensitivity and specificity

### Symptoms


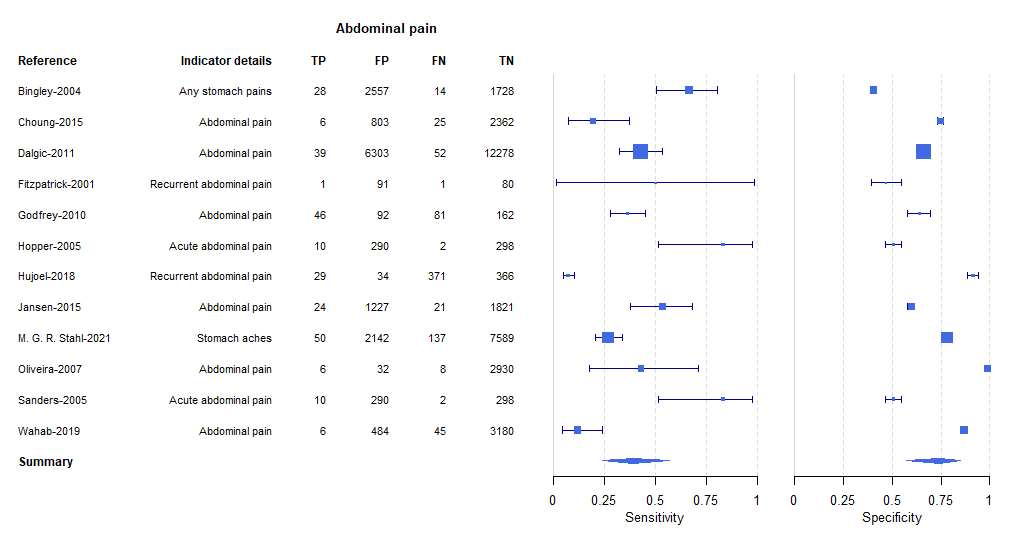


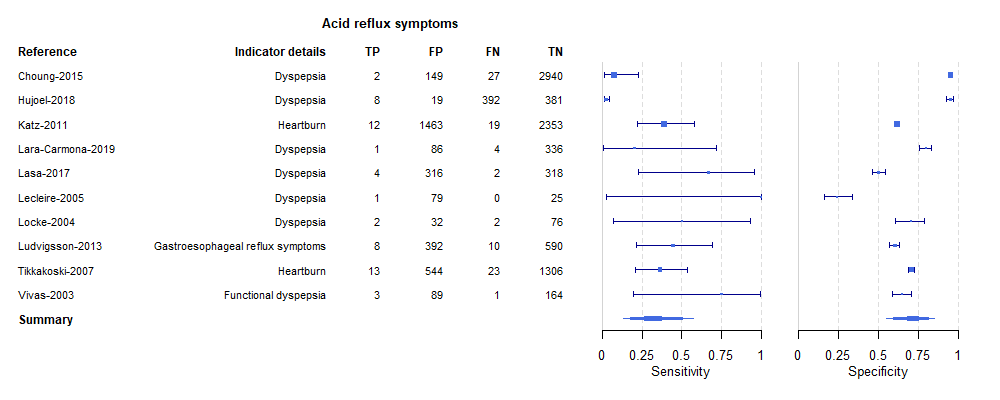


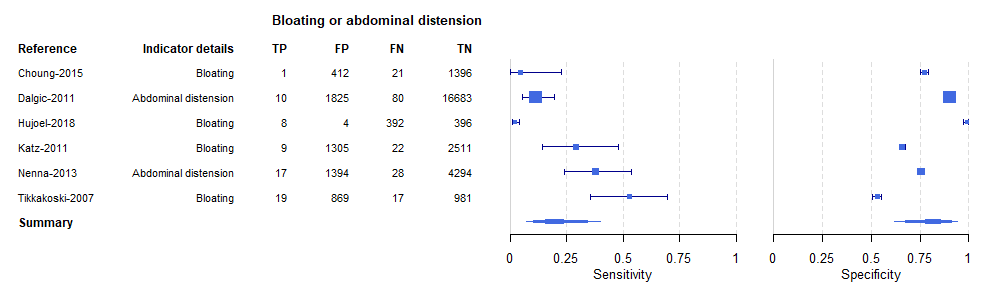


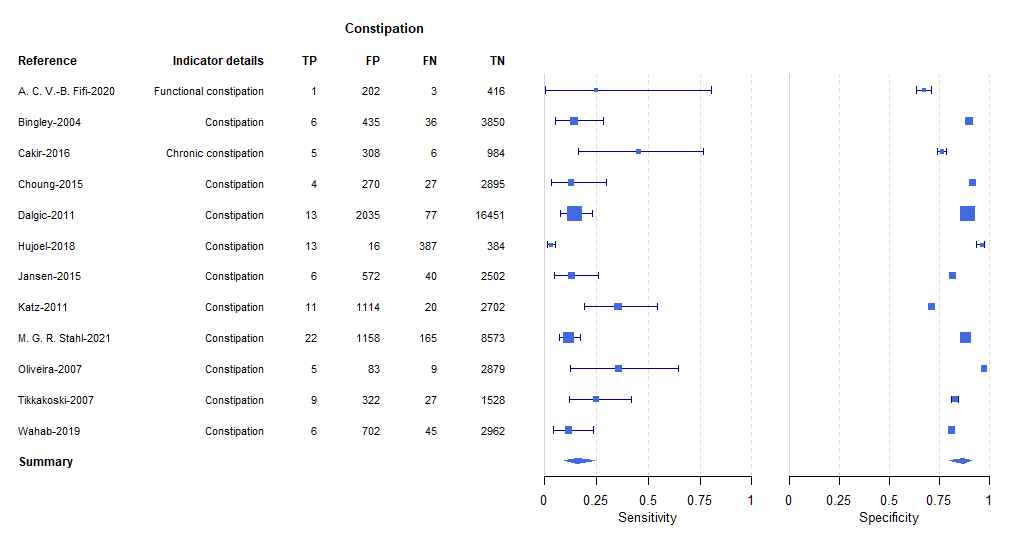


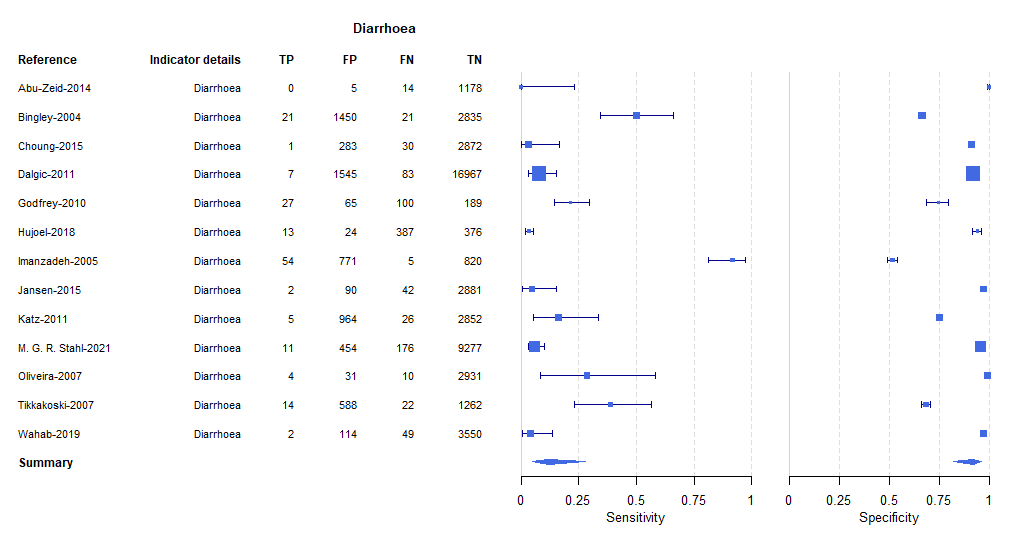


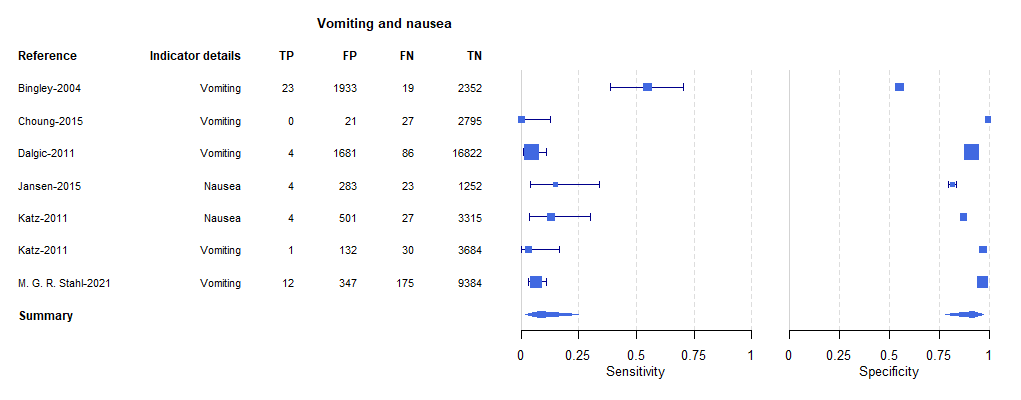


### Risk conditions


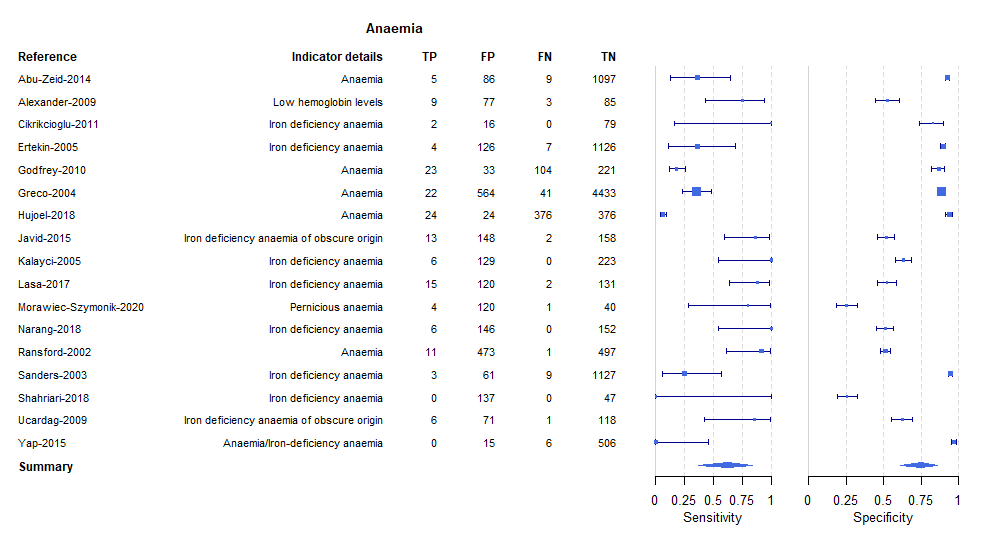


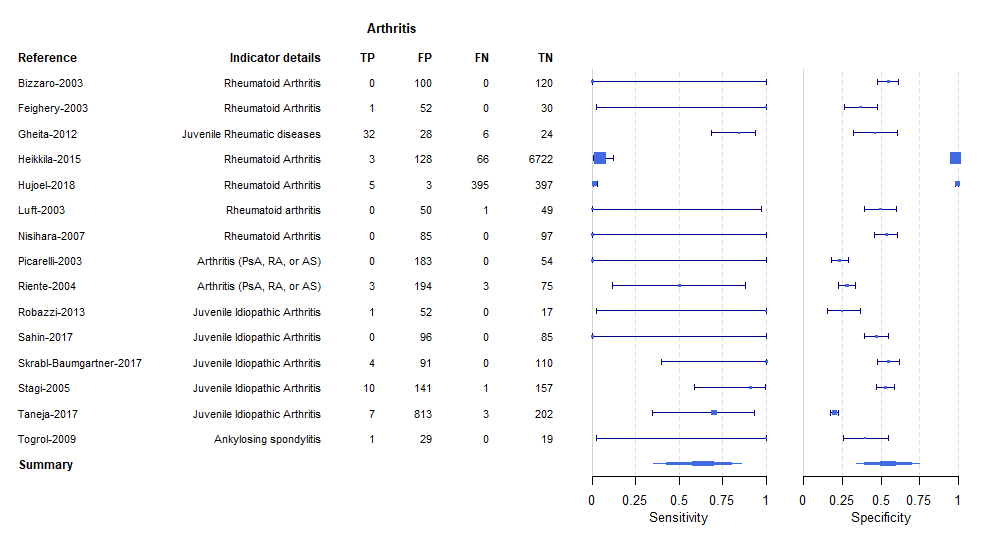


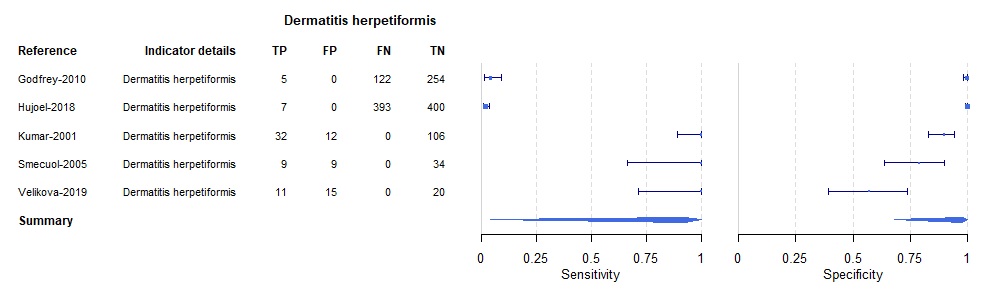


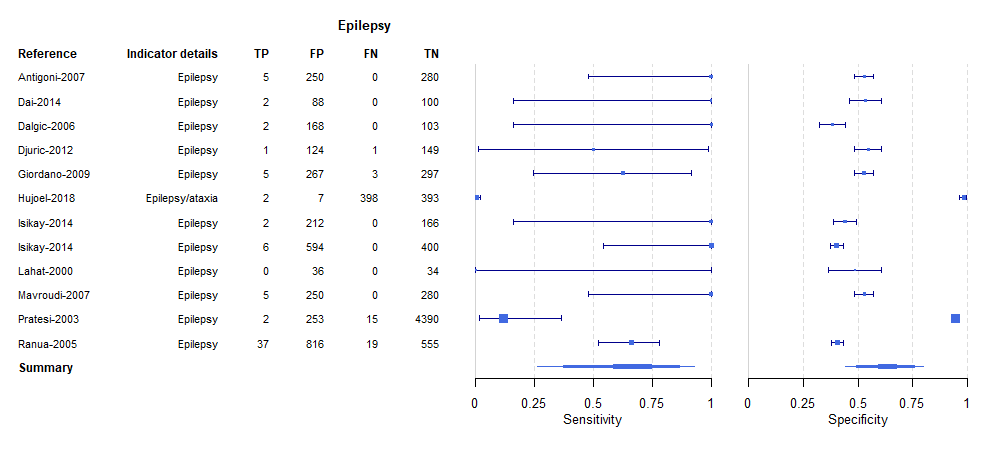


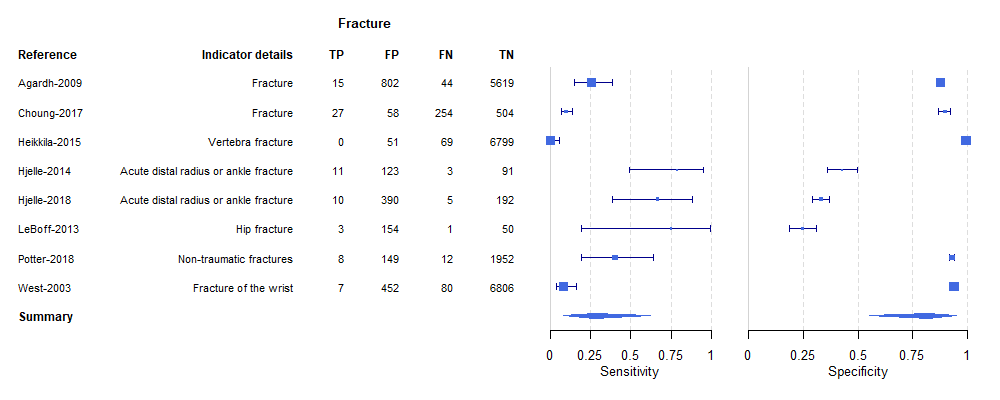


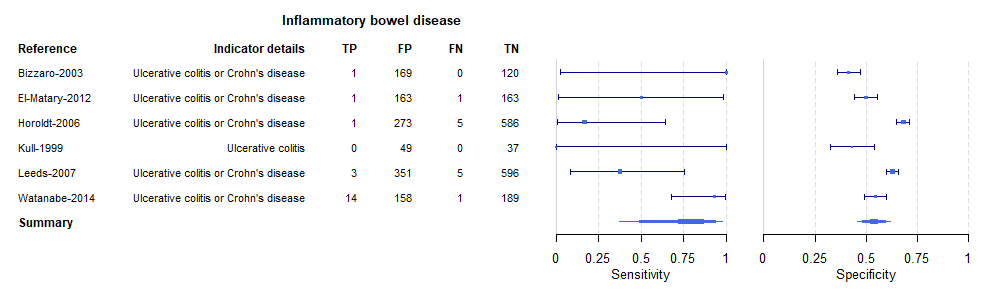


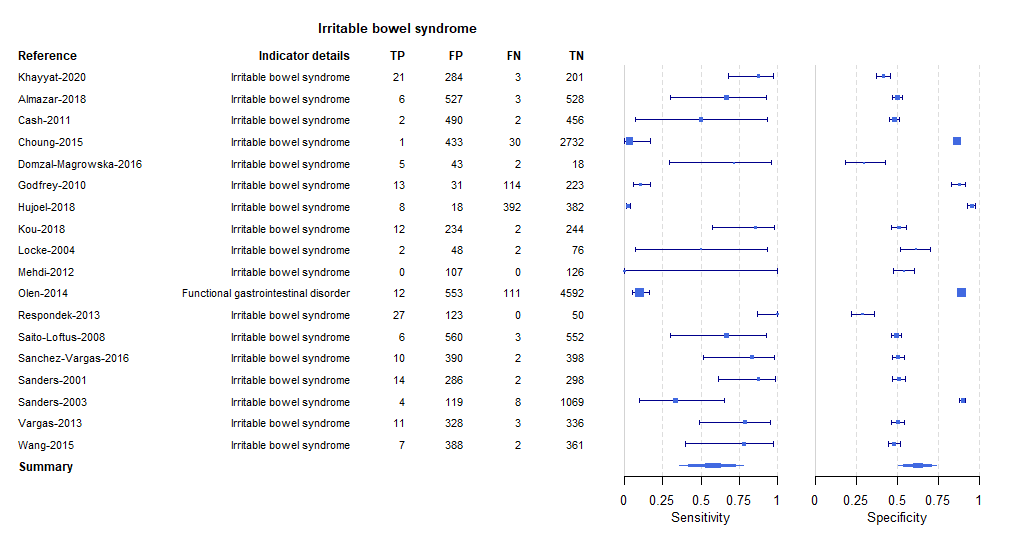


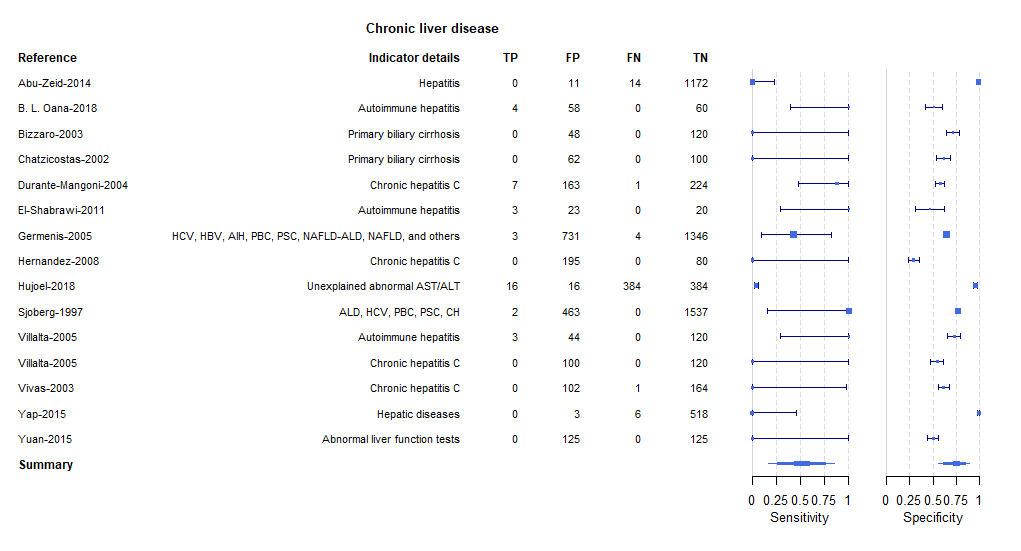


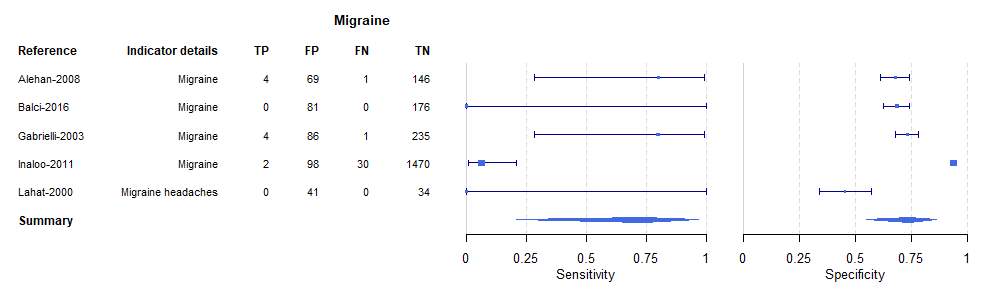


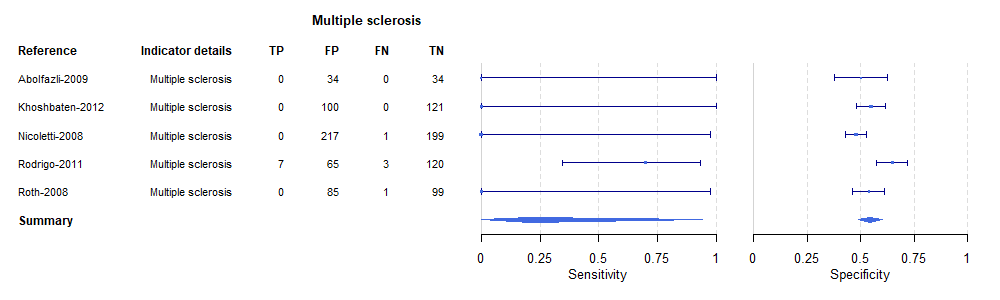


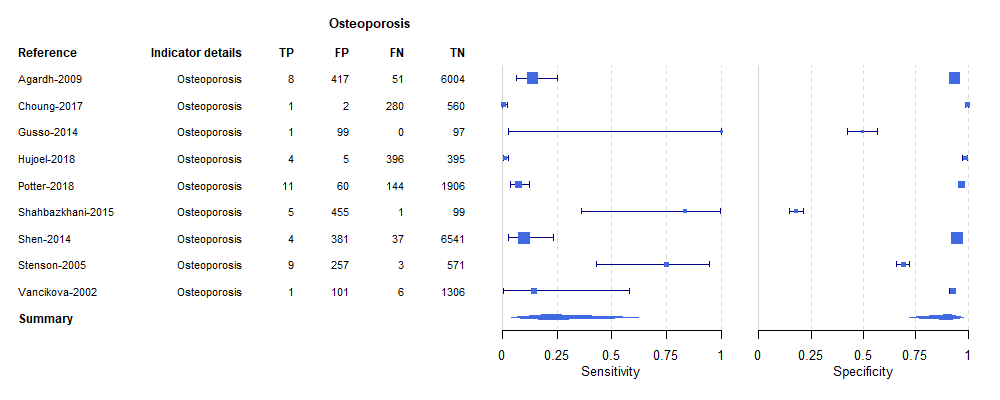


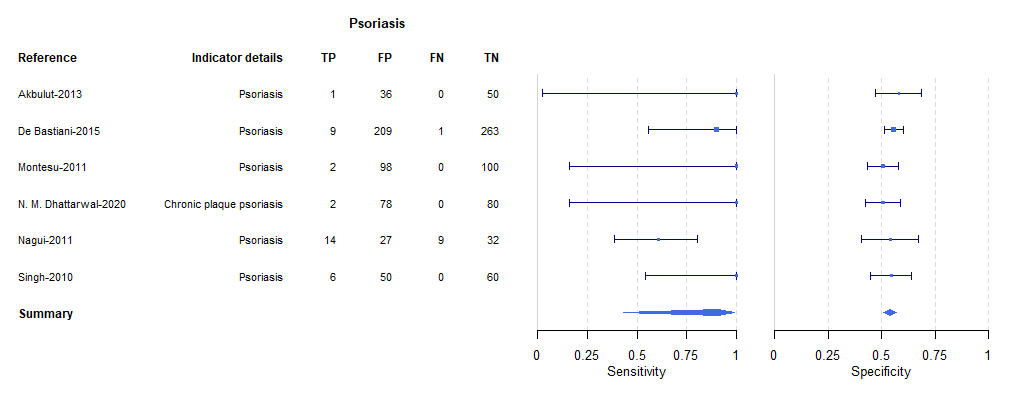


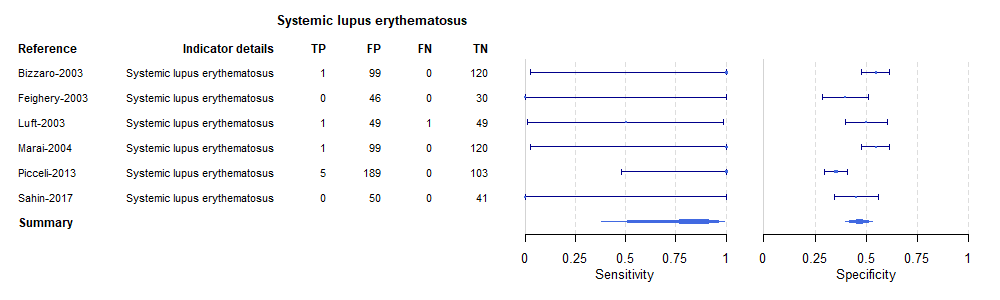


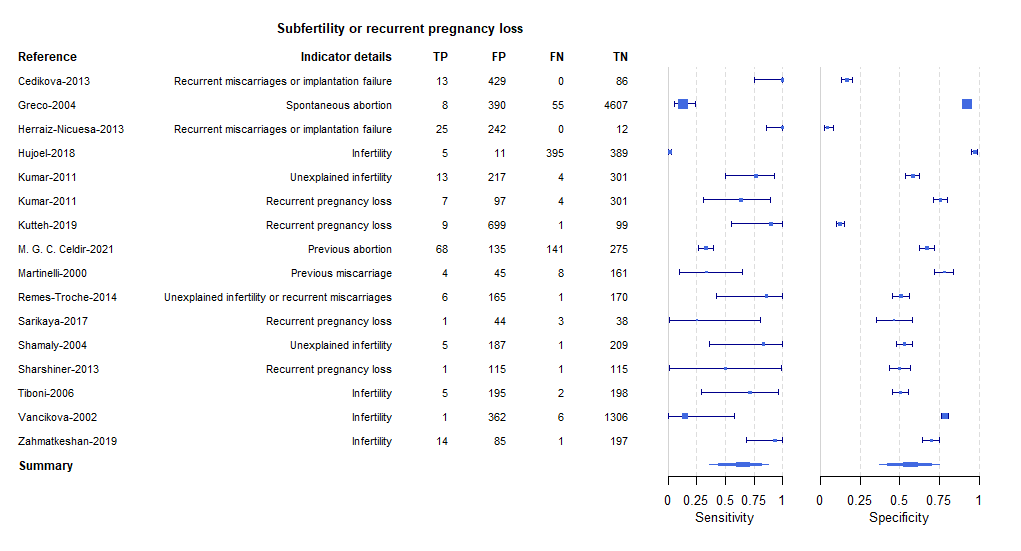


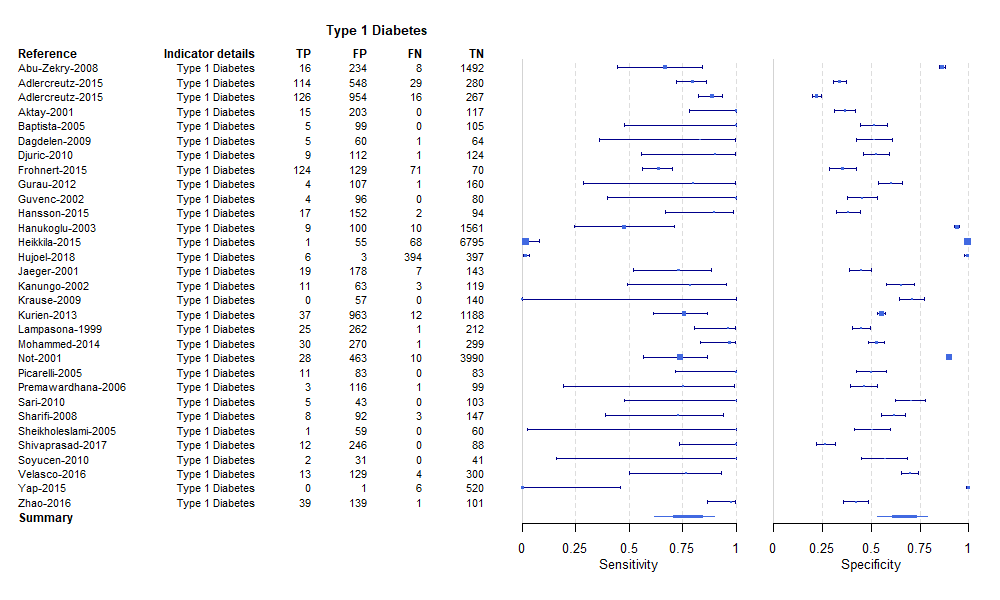


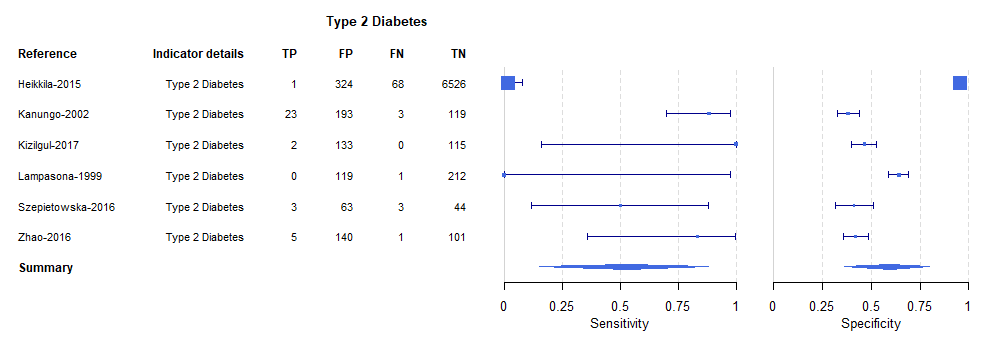


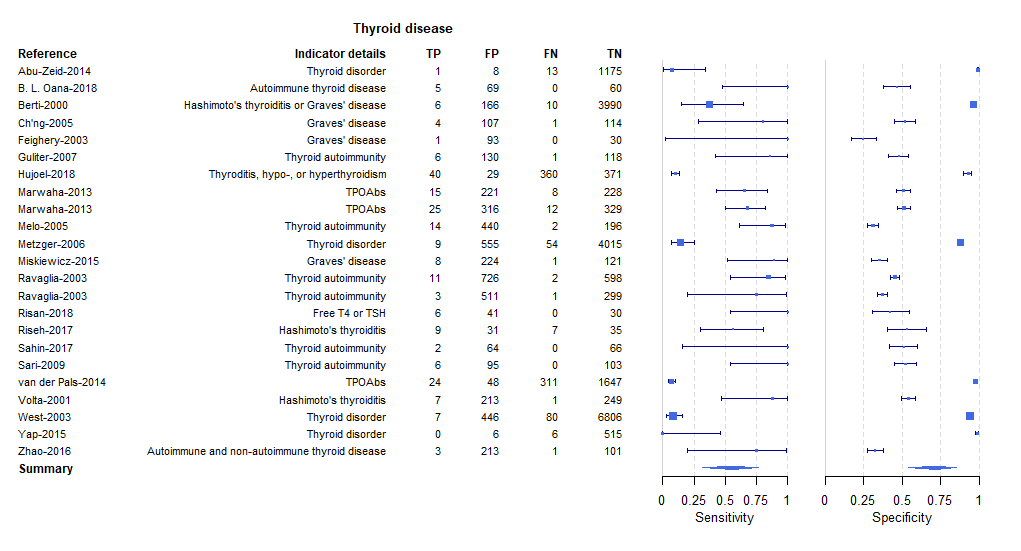


### Genetic predisposition


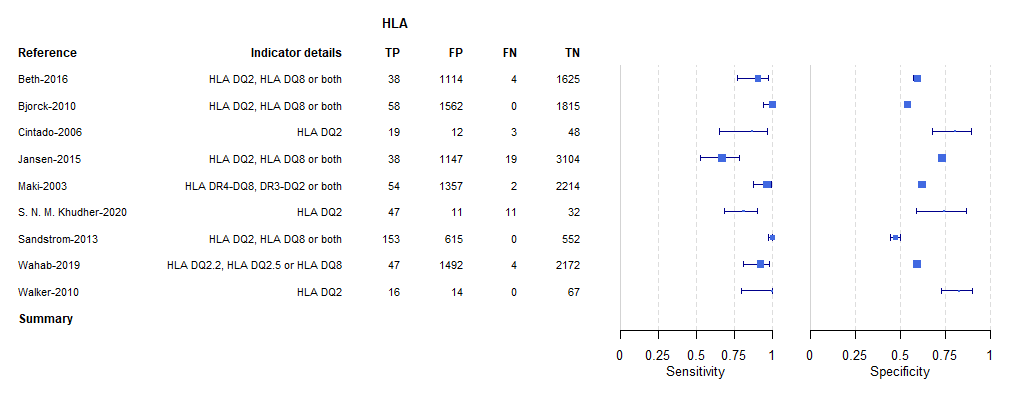


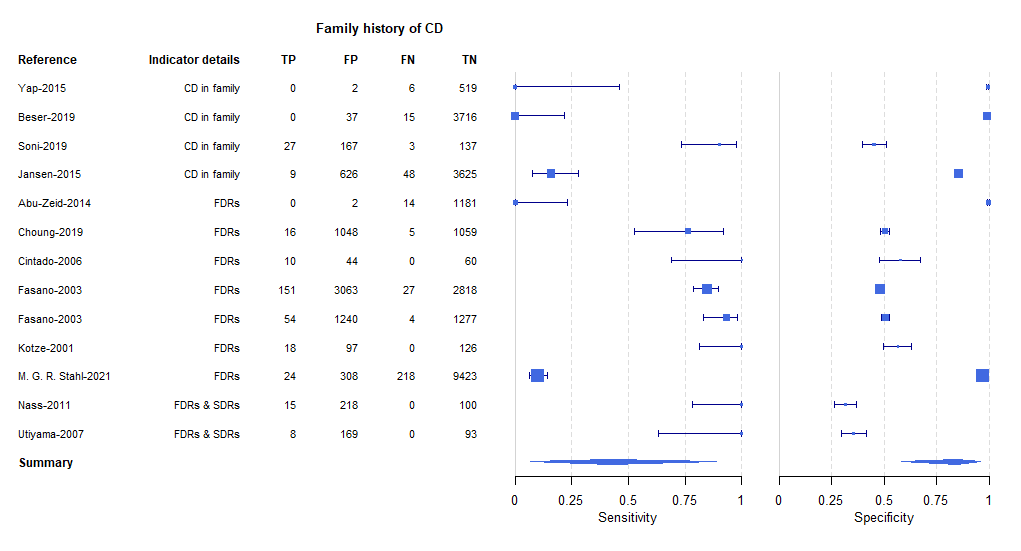

Supplement: S2 Fig — (DOCX) [file pone.0258501.s003.docx]
