# Supplementary material for: The accuracy of diagnostic indicators for coeliac disease: A systematic review and meta-analysis
Source: PLoS One. 2021 Oct 25;16(10):e0258501. doi: 10.1371/journal.pone.0258501 (PMC8545431; doi:10.1371/journal.pone.0258501)

## Figure S3: Summary ROC plots per diagnostic indicators.

Accuracy of symptoms (A), risk conditions (B), and genetic factors that predispose for coeliac disease (C) as diagnostic indicators. Blue line: HSROC line; green line: 95% confidence ellipse; red dot: summary estimate; circles: estimates per study, size is proportionate to the sample size.

#### A. Symptoms


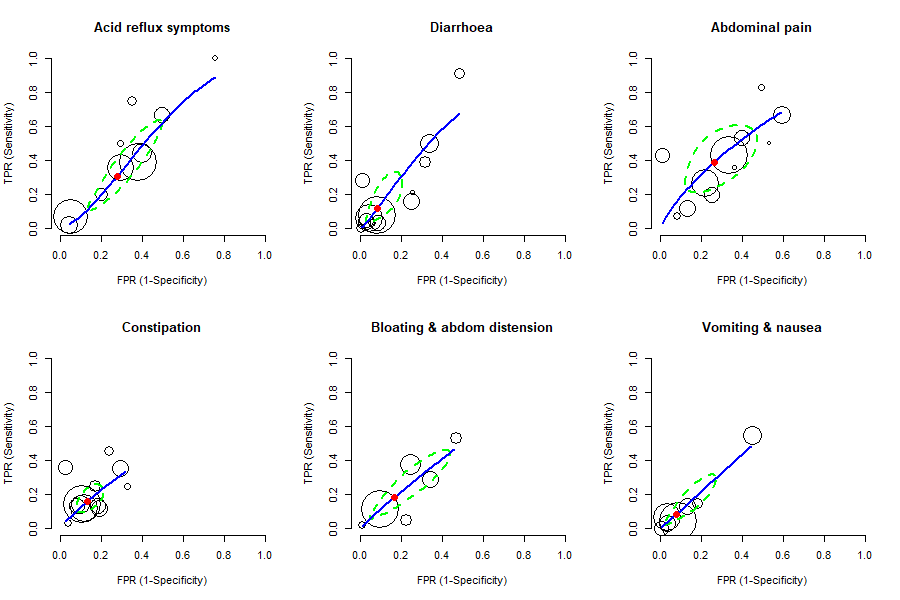


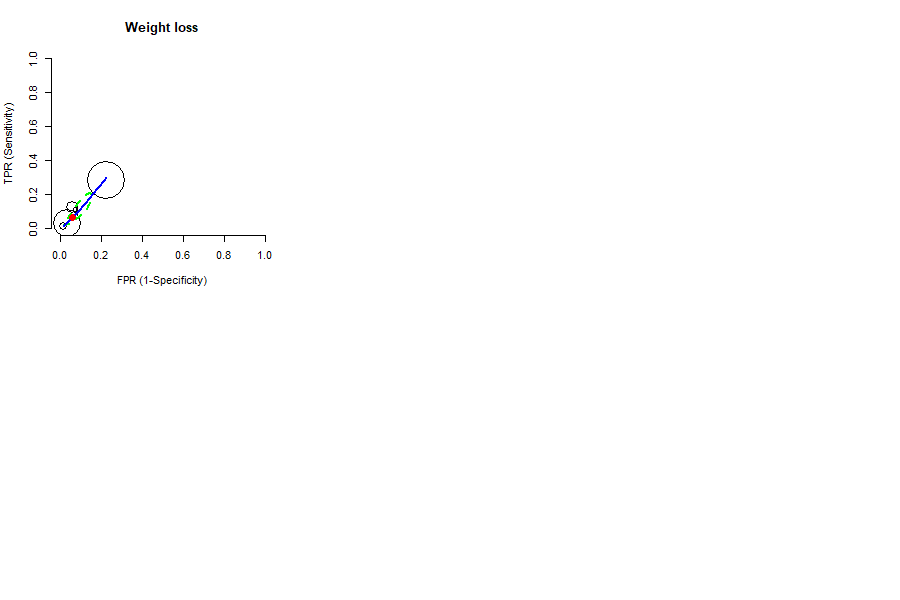


#### B. Risk conditions


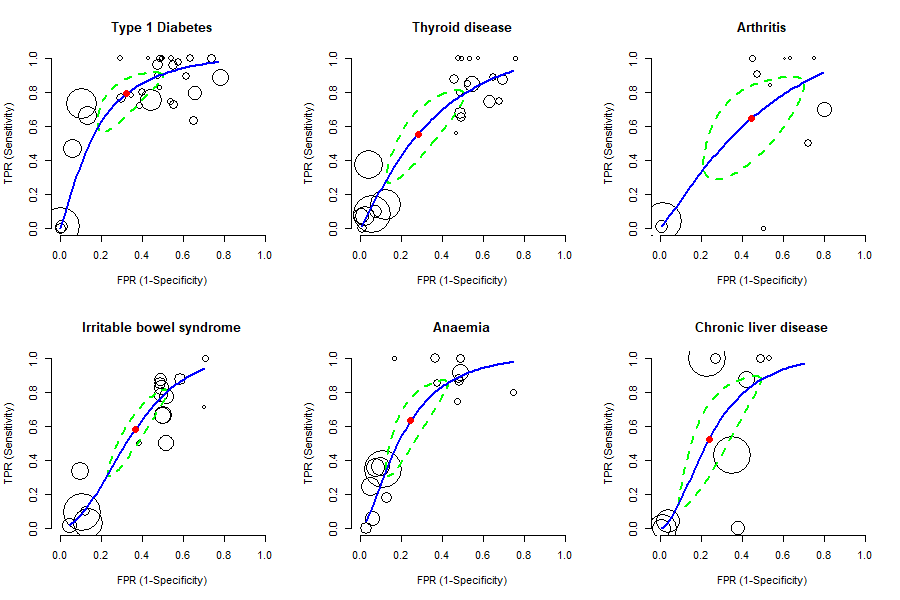


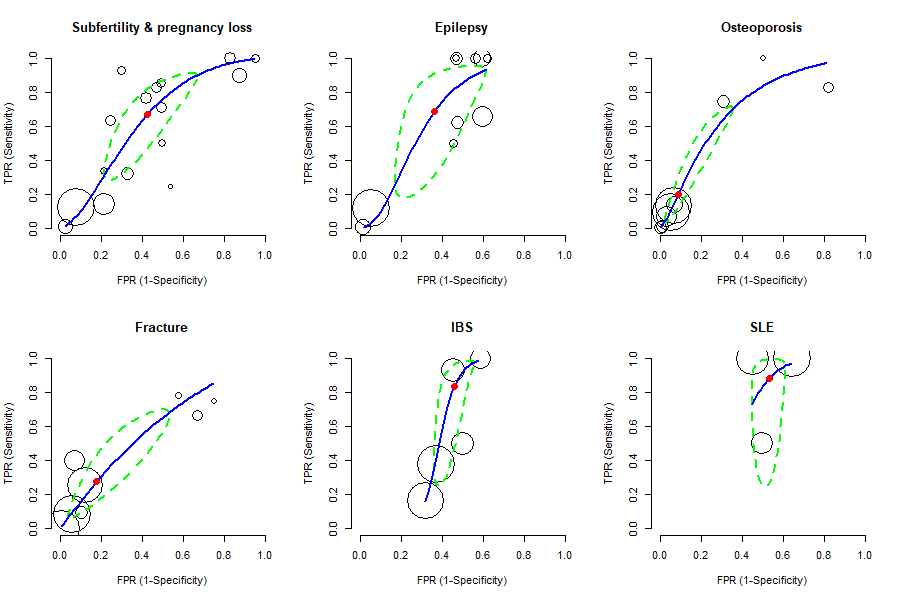


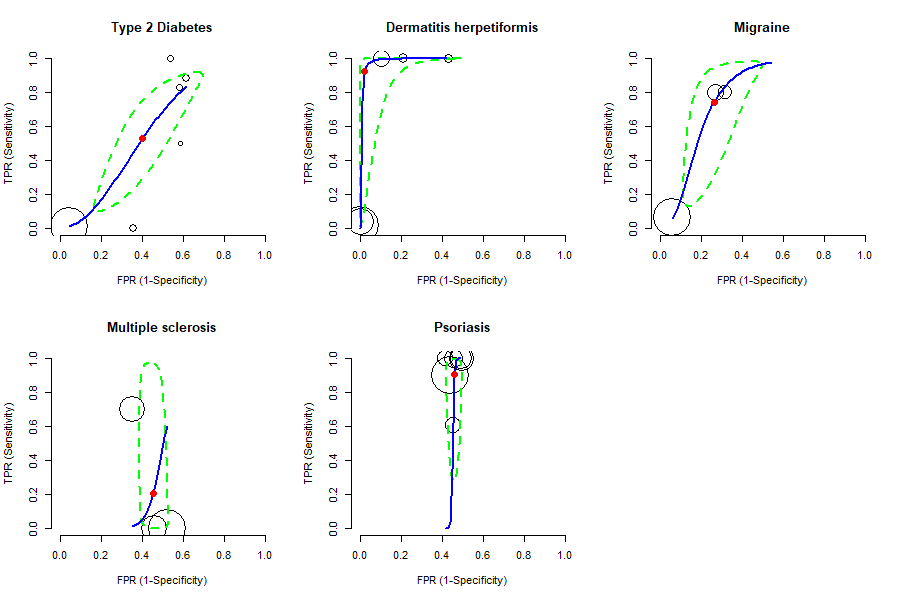


#### C. Genetic predisposition


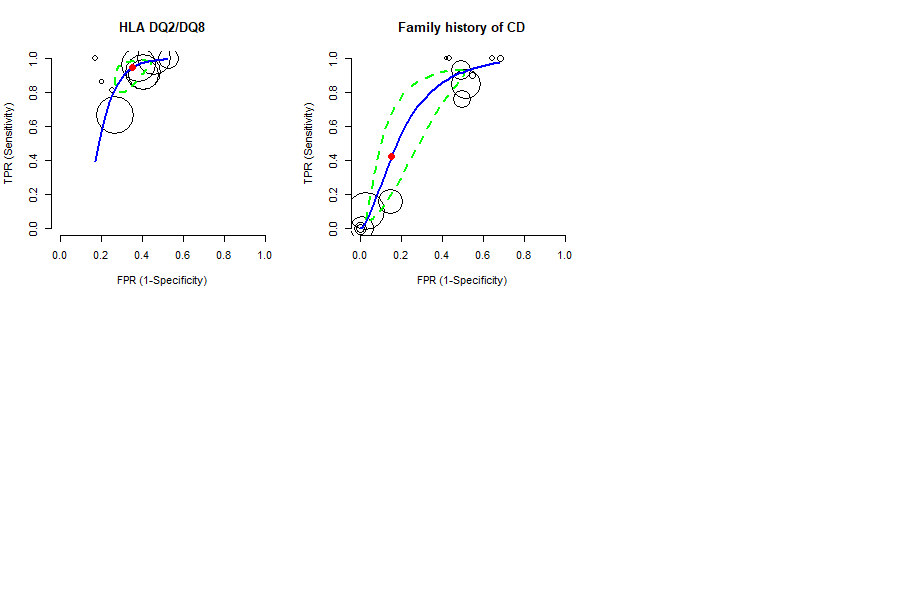

Supplement: S3 Fig — (DOCX) [file pone.0258501.s004.docx]
