# Supplementary material for: The accuracy of diagnostic indicators for coeliac disease: A systematic review and meta-analysis
Source: PLoS One. 2021 Oct 25;16(10):e0258501. doi: 10.1371/journal.pone.0258501 (PMC8545431; doi:10.1371/journal.pone.0258501)
Supplement: S1 Table — (DOCX) [file pone.0258501.s005.docx]

## Table S1: List of diagnostic indicators not included in the meta-analysis

Indicators with less than 5 identified studies were not included in the meta-analysis.

| **Diagnostic indicator** | **Number of studies** | **Reference** |
| --- | --- | --- |
| Autism | 4 | [1-4] |
| Cardiovascular disease | 4 | [5-8] |
| Low birth weight | 4 | [9-12] |
| Down syndrome | 3 | [13-15] |
| Familial Mediterranean fever | 3 | [16-18] |
| Fatigue | 3 | [19-21] |
| Flatulence | 3 | [19, 21, 22] |
| Sjogren's syndrome | 3 | [23-25] |
| Diabetes (unspecified) | 2 | [8, 26] |
| First degree relative with type 1 diabetes | 2 | [27, 28] |
| IgA nephropathy | 2 | [29, 30] |
| Iron deficiency | 2 | [19, 31] |
| Low BMI | 2 | [32, 33] |
| Low bone mineral density | 2 | [34, 35] |
| Lymphoma | 2 | [36, 37] |
| Osteopenia | 2 | [38, 39] |
| Short stature | 2 | [21, 40] |
| Small for gestational age | 2 | [11, 41] |
| Stillbirth | 2 | [42, 43] |
| Systemic sclerosis | 2 | [23, 25] |
| ADHD | 1 | [2] |
| Alzheimer<U+0092>s disease | 1 | [24] |
| Antiphospholipid syndrome | 1 | [44] |
| Apgar score <7 at 1 min | 1 | [42] |
| Apgar score <7 at 5 min | 1 | [42] |
| Aphthous stomatitis | 1 | [45] |
| APS3v | 1 | [46] |
| Asthma | 1 | [8] |
| Autoimmune cholangitis | 1 | [47] |
| Autoimmune haemolytic anaemia | 1 | [48] |
| Autoimmune myocarditis | 1 | [49] |
| Autoimmunity | 1 | [50] |
| Breech presentation | 1 | [42] |
| Bronchitis/emphysema | 1 | [8] |
| Cancer | 1 | [8] |
| Cholesterol | 1 | [8] |
| Chronic idiopathic thrombocytopenic purpura | 1 | [51] |
| Dental enamel defects | 1 | [52] |
| Dental staining | 1 | [21] |
| Depression | 1 | [53] |
| Difficulty swallowing | 1 | [54] |
| Enamel defects | 1 | [55] |
| Failure to thrive | 1 | [56] |
| H-Pylori infection | 1 | [57] |
| Headache | 1 | [26] |
| High BP | 1 | [8] |
| Hypertransaminasaemia | 1 | [58] |
| Idiopathic intrauterine growth restriction | 1 | [43] |
| Immune thrombocytopenia | 1 | [59] |
| Known neurological syndrome | 1 | [60] |
| Latent autoimmune diabetes in adults (LADA) | 1 | [61] |
| Lymphoid malignancies | 1 | [62] |
| Neurological dysfunction of unknown cause | 1 | [60] |
| Non-Hodgkin lymphoma | 1 | [63] |
| Obesity | 1 | [64] |
| Pallor | 1 | [65] |
| Pemphigus | 1 | [66] |
| Perinatal disease | 1 | [42] |
| Pregnancy term <37 weeks | 1 | [42] |
| Rheumatic fever | 1 | [67] |
| Scleroderma | 1 | [68] |
| Tumor | 1 | [26] |
| Turner's syndrome | 1 | [69] |
| Vitiligo | 1 | [70] |

## References

1. Abolfazli R, Mirbagheri SA, Zabihi AA, Abouzari M. Autism and celiac disease: Failure to validate the hypothesis of a possible link. Iranian Red Crescent Medical Journal. 2009;11(4):442-4.

2. Bala KA, Dogan M, Kaba S, Mutluer T, Aslan O, Dogan SZ. Hormone disorder and vitamin deficiency in attention deficit hyperactivity disorder (ADHD) and autism spectrum disorders (ASDs). Journal of Pediatric Endocrinology & Metabolism. 2016;29(9):1077-82. doi: 10.1515/jpem-2015-0473. PubMed PMID: WOS:000384418000011.

3. de Magistris L, Picardi A, Siniscalco D, Riccio MP, Sapone A, Cariello R, et al. Antibodies against Food Antigens in Patients with Autistic Spectrum Disorders. Biomed Research International. 2013. doi: 10.1155/2013/729349. PubMed PMID: WOS:000322981400001.

4. Lau NM, Green PHR, Taylor AK, Hellberg D, Ajamian M, Tan CZ, et al. Markers of Celiac Disease and Gluten Sensitivity in Children with Autism. PLoS ONE. 2013;8(6):e66155. doi: http://dx.doi.org/10.1371/journal.pone.0066155.

5. Heikkila K, Rissanen H, Heliovaara M, Knekt P, Maki M, Kaukinen K. Associations of tissue transglutaminase antibody seropositivity with coronary heart disease: Findings from a prospective cohort study. Nutrition, Metabolism and Cardiovascular Diseases. 2017;27(9):817-21. doi: http://dx.doi.org/10.1016/j.numecd.2017.06.005.

6. Balci O, Sezer T. The prevalence of celiac disease in children with arterial ischemic stroke. Journal of Pediatric Hematology/Oncology. 2017;39(1):46-9. doi: http://dx.doi.org/10.1097/MPH.0000000000000661.

7. Di Tola M, Barilla F, Trappolini M, Palumbo HF, Gaudio C, Picarelli A. Antitissue transglutaminase antibodies in acute coronary syndrome: an alert signal of myocardial tissue lesion? Journal of Internal Medicine. 2008;263(1):43-51. doi: 10.1111/j.1365-2796.2007.01881.x. PubMed PMID: WOS:000251553800005.

8. West J, Logan RFA, Hill PG, Lloyd A, Lewis S, Hubbard R, et al. Seroprevalence, correlates, and characteristics of undetected coeliac disease in England. Gut. 2003;52(7):960-5. doi: http://dx.doi.org/10.1136/gut.52.7.960.

9. Celdir MG, Choung RS, Rostamkolaei SK, King KS, Larson JJ, Absah I, et al. CELIAC AUTOIMMUNITY AND PREGNANCY OUTCOMES. Gastroenterology. 2019;156(6 S1):S-917. doi: http://dx.doi.org/10.1016/S0016-5085%2819%2939261-3.

10. Ozgor B, Selimoglu MA, Temel I, Seckin Y, Kafkasli A. Prevalence of celiac disease in parents of preterm or low birthweight newborns. The journal of obstetrics and gynaecology research. 2011;37(11):1615-9. doi: https://dx.doi.org/10.1111/j.1447-0756.2011.01584.x.

11. Kiefte-De Jong JC, Jaddoe VWV, Uitterlinden AG, Steegers EAP, Willemsen SP, Hofman A, et al. Levels of antibodies against tissue transglutaminase during pregnancy are associated with reduced fetal weight and birth weight. Gastroenterology. 2013;144(4):726. doi: http://dx.doi.org/10.1053/j.gastro.2013.01.003.

12. Sanchez GS, Cristobal CB, Gonzalez AS, Llorente FJR, Rodriguez EF, Gonzalez NF, et al. Maternal non-diagnosed celiac disease and risk of low birth weight. Revista Espanola De Enfermedades Digestivas. 2008;100(6):332-6. PubMed PMID: WOS:000258204700004.

13. Nisihara RM, Kotze LMS, Utiyama SRR, Oliveira NP, Fiedler PT, Messias-Reason IT. Celiac disease in children and adolescents with Down syndrome. Jornal de Pediatria. 2005;81(5):373-6. doi: http://dx.doi.org/10.2223/JPED.1381.

14. Hansson T, Dahlbom I, Rogberg S, Nyberg BI, Dahlstrom J, Anneren G, et al. Antitissue transglutaminase and antithyroid autoantibodies in children with Down syndrome and celiac disease. Journal of Pediatric Gastroenterology and Nutrition. 2005;40(2):170-4. doi: http://dx.doi.org/10.1097/00005176-200502000-00016.

15. Shamaly H, Hartman C, Pollack S, Hujerat M, Katz R, Gideoni O, et al. Tissue transglutaminase antibodies are a useful serological marker for the diagnosis of celiac disease in patients with down syndrome. Journal of Pediatric Gastroenterology and Nutrition. 2007;44(5):583-6. doi: http://dx.doi.org/10.1097/MPG.0b013e3180320679.

16. Sahin Y, Adrovic A, Barut K, Kutlu T, Cullu-Cokugras F, Sahin S, et al. The frequency of the celiac disease among children with familial Mediterranean fever. Modern Rheumatology. 2017;27(6):1036-9. doi: http://dx.doi.org/10.1080/14397595.2016.1270497.

17. Sahin Y, Barut K, Kutlu T, Cokugras FC, Adrovic A, Sahin S, et al. The frequency of celiac disease in children with colchicine-resistant familial Mediterranean fever. Journal of Pediatric Gastroenterology and Nutrition. 2017;64(Supplement 1):228. doi: http://dx.doi.org/10.1097/01.mpg.0000516381.25680.b4.

18. Isikay S, Isikay N, Kocamaz H. The prevalence of celiac disease among patients with familial mediterranean fever. Arquivos de Gastroenterologia. 2015;52(1):55-8. doi: http://dx.doi.org/10.1590/S0004-28032015000100012.

19. Hujoel IA, Van Dyke CT, Brantner T, Larson J, King KS, Sharma A, et al. Natural history and clinical detection of undiagnosed coeliac disease in a North American community. Alimentary Pharmacology and Therapeutics. 2018;47(10):1358-66. doi: http://dx.doi.org/10.1111/apt.14625.

20. Sanders DS, Patel D, Stephenson TJ, Ward AM, McCloskey EV, Hadjivassiliou M, et al. A primary care cross-sectional study of undiagnosed adult coeliac disease. European Journal of Gastroenterology and Hepatology. 2003;15(4):407-13. doi: http://dx.doi.org/10.1097/00042737-200304000-00012.

21. Dalgic B, Sari S, Ozcan B, Basturk B, Ensari A, Egritas O, et al. The evaluation of factors and symptoms related to celiac disease in Turkish children. Turk Pediatri Arsivi. 2011;46(4):314-21. doi: http://dx.doi.org/10.4274/tpa.672.

22. Tikkakoski S, Savilahti E, Kolho K-L. Undiagnosed coeliac disease and nutritional deficiencies in adults screened in primary health care. Scandinavian journal of gastroenterology. 2007;42(1):60-5.

23. Bizzaro N, Villalta D, Tonutti E, Doria A, Tampoia M, Bassetti D, et al. IgA and IgG Tissue Transglutaminase Antibody Prevalence and Clinical Significance in Connective Tissue Diseases, Inflammatory Bowel Disease, and Primary Biliary Cirrhosis. Digestive Diseases and Sciences. 2003;48(12):2360-5. doi: http://dx.doi.org/10.1023/B:DDAS.0000007875.72256.e8.

24. Roth EB, Theander E, Londos E, Sandberg-Wollheim M, Larsson A, Sjoberg K, et al. Pathogenesis of autoimmune diseases: Antibodies against transglutaminase, peptidylarginine deiminase and protein-bound citrulline in primary Sjogren's syndrome, multiple sclerosis and Alzheimer's disease. Scandinavian Journal of Immunology. 2008;67(6):626-31. doi: 10.1111/j.1365-3083.2008.02115.x. PubMed PMID: WOS:000255725000012.

25. Luft LM, Barr SG, Martin LO, Chan EKL, Fritzler MJ. Autoantibodies to Tissue Transglutaminase in Sjogren's Syndrome and Related Rheumatic Diseases. Journal of Rheumatology. 2003;30(12):2613-9.

26. Abu-Zeid YA, Jasem WS, Lebwohl B, Green PH, ElGhazali G. Seroprevalence of celiac disease among United Arab Emirates healthy adult nationals: A gender disparity. World Journal of Gastroenterology. 2014;20(42):15830-6. doi: http://dx.doi.org/10.3748/wjg.v20.i42.15830.

27. Mohammed MA, Elrabbat AM, Omar NM, Shebl AM, Mansour AH, Elmasry E, et al. Celiac disease prevalence and its HLA-genotypic profile in Egyptian patients with type 1 diabetes mellitus. Trends in Medical Research. 2014;9(2):81-97. doi: http://dx.doi.org/10.3923/tmr.2014.81.97.

28. Not T, Tommasini A, Tonini G, Buratti E, Pocecco M, Tortul C, et al. Undiagnosed coeliac disease and risk of autoimmune disorders in subjects with Type I diabetes mellitus. Diabetologia. 2001;44(2):151-5. doi: 10.1007/s001250051593. PubMed PMID: WOS:000167005200003.

29. Moeller S, Canetta PA, Taylor AK, Arguelles-Grande C, Snyder H, Green PH, et al. Lack of serologic evidence to link IgA nephropathy with celiac disease or immune reactivity to gluten. PLoS ONE. 2014;9(4):e94677. doi: http://dx.doi.org/10.1371/journal.pone.0094677.

30. Ots M, Uibo O, Metskula K, Uibo R, Salupere V. IgA-antigliadin antibodies in patients with IgA nephropathy: The secondary phenomenon? American Journal of Nephrology. 1999;19(4):453-8. doi: http://dx.doi.org/10.1159/000013497.

31. Murray JA, McLachlan S, Adams PC, Eckfeldt JH, Garner CP, Vulpe CD, et al. Association between celiac disease and iron deficiency in caucasians, but not non-caucasians. Clinical Gastroenterology and Hepatology. 2013;11(7):808-14. doi: http://dx.doi.org/10.1016/j.cgh.2013.02.009.

32. van der Pals M, Myleus A, Norstrom F, Hammarroth S, Hogberg L, Rosen A, et al. Body mass index is not a reliable tool in predicting celiac disease in children. BMC Pediatrics. 2014;14(1):165. doi: http://dx.doi.org/10.1186/1471-2431-14-165.

33. Yuan J, Zhou C, Gao J, Li J, Yu F, Lu J, et al. Prevalence of Celiac Disease Autoimmunity Among Adolescents and Young Adults in China. Clinical Gastroenterology and Hepatology. 2017;15(10):1572. doi: http://dx.doi.org/10.1016/j.cgh.2017.04.025.

34. Ben Hariz M, Kallel-Sellami M, Kallel L, Lahmer A, Halioui S, Bouraoui S, et al. Prevalence of celiac disease in Tunisia: mass-screening study in schoolchildren. European journal of gastroenterology & hepatology. 2007;19(8):687-94.

35. Hariz MB, Kallel-Sellami M, Kallel L, Lahmer A, Halioui S, Bouraoui S, et al. Prevalence of celiac disease in Tunisia: Mass-screening study in schoolchildren. European Journal of Gastroenterology and Hepatology. 2007;19(8):687-94. doi: http://dx.doi.org/10.1097/MEG.0b013e328133f0c1.

36. Rubio-Tapia A, Murray JA, Fredericksen ZS, Liebow M, Dogan A, Habermann TM, et al. Celiac disease is rare among patients with follicular lymphoma, diffuse large b-cell lymphoma, and hodgkin lymphoma: A preliminary report from the lymphoma spore molecular epidemiology resource. Gastroenterology. 2012;142(5 SUPPL. 1):S275-S6.

37. Carroccio A, Iannitto E, Di Prima L, Cirrincione S, Troncone R, Paparo F, et al. Screening for celiac disease in non-Hodgkin's lymphoma patients: A serum anti-transglutaminase-based approach. Digestive Diseases and Sciences. 2003;48(8):1530-6. doi: http://dx.doi.org/10.1023/A:1024811707311.

38. Choung RS, Larson SA, Khaleghi S, Rubio-Tapia A, Ovsyannikova IG, King KS, et al. Prevalence and Morbidity of Undiagnosed Celiac Disease From a Community-Based Study. Gastroenterology. 2017;152(4):830-9. doi: http://dx.doi.org/10.1053/j.gastro.2016.11.043.

39. Agardh D, Bjorck S, Agardh CD, Lidfeldt J. Coeliac disease-specific tissue transglutaminase autoantibodies are associated with osteoporosis and related fractures in middle-aged women. Scandinavian Journal of Gastroenterology. 2009;44(5):571-8. doi: http://dx.doi.org/10.1080/00365520902718929.

40. Ahmad F, Alam S, Shukla I, Sherwani R, Ali SM. Screening children with severe short stature for celiac disease using tissue transglutaminase. Indian Journal of Pediatrics. 2010:1-4. doi: http://dx.doi.org/10.1007/s12098-010-0040-2.

41. Baldassarre ME, Laneve A, Fontana A, Manca F, Salvia G, Barcaglioni P, et al. Usefulness of tissue transglutaminase type 2 antibodies in early pregnancy. Immunopharmacology and Immunotoxicology. 2012;34(6):932-6. doi: 10.3109/08923973.2012.680470. PubMed PMID: WOS:000310314800008.

42. Martinelli P, Troncone R, Paparo F, Torre P, Trapanese E, Fasano C, et al. Coeliac disease and unfavourable outcome of pregnancy. Gut. 2000;46(3):332-5.

43. Kumar A, Meena M, Begum N, Kumar N, Gupta RK, Aggarwal S, et al. Latent celiac disease in reproductive performance of women. Fertility and Sterility. 2011;95(3):922-7. doi: http://dx.doi.org/10.1016/j.fertnstert.2010.11.005.

44. Shamir R, Shoenfeld Y, Blank M, Eliakim R, Lahat N, Sobel E, et al. The prevalence of coeliac disease antibodies in patients with the antiphospholipid syndrome. Lupus. 2003;12(5):394-9. doi: http://dx.doi.org/10.1191/0961203303lu384oa.

45. Bijelic B, Matic IZ, Besu I, Jankovic L, Juranic Z, Marusic S, et al. Celiac disease-specific and inflammatory bowel disease-related antibodies in patients with recurrent aphthous stomatitis. Immunobiology. 2019;224(1):75-9. doi: 10.1016/j.imbio.2018.10.006. PubMed PMID: WOS:000460999400009.

46. Zhao Z, Zou J, Zhao L, Cheng Y, Cai H, Li M, et al. Celiac disease autoimmunity in patients with autoimmune diabetes and thyroid disease among Chinese population. PLoS ONE. 2016;11(7):e0157510. doi: http://dx.doi.org/10.1371/journal.pone.0157510.

47. Chatzicostas C, Roussomoustakaki M, Drygiannakis D, Niniraki M, Tzardi M, Koulentaki M, et al. Primary biliary cirrhosis and autoimmune cholangitis are not associated with coeliac disease in Crete. BMC Gastroenterology. 2002;2:5. doi: http://dx.doi.org/10.1186/1471-230X-2-5.

48. Gabrielli M, Candelli M, Franceschi F, Cremonini F, Nista EC, Santarelli L, et al. Primary autoimmune haemolytic anaemia and coeliac disease. Scandinavian journal of gastroenterology. 2004;39(6):605-6.

49. Frustaci A, Cuoco L, Chimenti C, Pieroni M, Fioravanti G, Gentiloni N, et al. Celiac disease associated with autoimmune myocarditis. Circulation. 2002;105(22):2611-8. doi: http://dx.doi.org/10.1161/01.CIR.0000017880.86166.87.

50. Vancikova Z, Chlumecky V, Sokol D, Horakova D, Hamsikova E, Fucikova T, et al. The serologic screening for celiac disease in the general population (blood donors) and in some high-risk groups of adults (patients with autoimmune diseases, osteoporosis and infertility) in the Czech republic. Folia microbiologica. 2002;47(6):753-8.

51. Altintas A, Pasa S, Cil T, Bayan K, Gokalp D, Ayyildiz O. Thyroid and celiac diseases autoantibodies in patients with adult chronic idiopathic thrombocytopenic purpura. Platelets. 2008;19(4):252-7. doi: 10.1080/09537100801894651. PubMed PMID: WOS:000257069000002.

52. El-Hodhod MA-A, El-Agouza IA, Abdel-Al H, Kabil NS, Bayomi KAE-M. Screening for celiac disease in children with dental enamel defects. ISRN pediatrics. 2012;2012:763783. doi: https://dx.doi.org/10.5402/2012/763783.

53. Khoshbaten M, Rostami Nejad M, Sharifi N, Fakhari A, Golamnejad M, Hashemi SH, et al. Celiac disease in patients with chronic psychiatric disorders. Gastroenterology and hepatology from bed to bench. 2012;5(2):90-3.

54. Choung RS, Rubio-Tapia A, Lahr BD, Kyle RA, Camilleri MJ, Locke GR, et al. Evidence Against Routine Testing of Patients With Functional Gastrointestinal Disorders for Celiac Disease: A Population-based Study. Clinical Gastroenterology and Hepatology. 2015;13(11):1937-43. doi: http://dx.doi.org/10.1016/j.cgh.2015.05.014.

55. Beth SA, Jansen MAE, Elfrink MEC, Kiefte-de Jong JC, Wolvius EB, Jaddoe VWV, et al. Generation R birth cohort study shows that specific enamel defects were not associated with elevated serum transglutaminase type 2 antibodies. Acta Paediatrica. 2016;105(10):e485-e91. doi: 10.1111/apa.13533. PubMed PMID: WOS:000383619400009.

56. Ertekin V, Selimoglu MA, Kardas F, Aktas E. Prevalence of celiac disease in Turkish children. Journal of Clinical Gastroenterology. 2005;39(8):689-91. doi: http://dx.doi.org/10.1097/01.mcg.0000174026.26838.56.

57. Borch K, Grodzinsky E, Petersson F, Jonsson KA, Mardh S, Valdimarsson T. Prevalence of coeliac disease and relations to Helicobacter pylori infection and duodenitis in a Swedish adult population sample: A histomorphological and serological survey. Inflammopharmacology. 2000;8(4):341-50. doi: http://dx.doi.org/10.1163/156856000750264401.

58. Vivas S, Ruiz De Morales JM, Martinez J, Gonzalez MC, Martin S, Martin J, et al. Human recombinant anti-transglutaminase antibody testing is useful in the diagnosis of silent coeliac disease in a selected group of at-risk patients. European Journal of Gastroenterology and Hepatology. 2003;15(5):479-83. doi: http://dx.doi.org/10.1097/01.meg.0000059104.41030.1c.

59. Karunakaran P, Kochhar R, Lal S, Nampoothiri RV, Varma N, Varma S, et al. High Prevalence of Celiac Disease in Patients with Immune Thrombocytopenia. Indian Journal of Hematology and Blood Transfusion. 2019;35(4):722-5. doi: http://dx.doi.org/10.1007/s12288-019-01120-x.

60. Ruggieri M, Incorpora G, Polizzi A, Parano E, Spina M, Pavone P. Low prevalence of neurologic and psychiatric manifestations in children with gluten sensitivity. The Journal of pediatrics. 2008;152(2):244-9. doi: https://dx.doi.org/10.1016/j.jpeds.2007.06.042.

61. Szepietowska B, Wawrusiewicz-Kurylonek N, Kretowski A, Gorska M, Szelachowska M. Endocrine autoimmunity in patients with Latent Autoimmune Diabetes in Adults (LADA) - association with HLA genotype. Endokrynologia Polska. 2016;67(2):197-201. doi: 10.5603/EP.a2016.0017. PubMed PMID: WOS:000374546600007.

62. Sjoberg K, Roth EB, Gustavsson L, Jonsson C, Siman H, Henriksson G, et al. Autoimmune markers in lymphoid malignancies. Scandinavian Journal of Immunology. 2008;67(5):509-15. doi: 10.1111/j.1365-3083.2008.02095.x. PubMed PMID: WOS:000254812400012.

63. Mearin ML, Catassi C, Brousse N, Brand R, Collin P, Fabiani E, et al. European multi-centre study on coeliac disease and non-Hodgkin lymphoma. European journal of gastroenterology & hepatology. 2006;18(2):187-94.

64. Oliveira RP, Sdepanian VL, Barreto JA, Cortez AJP, Carvalho FO, Bordin JO, et al. High prevalence of celiac disease in Brazilian blood donor volunteers based on screening by IgA antitissue transglutaminase antibody. European Journal of Gastroenterology & Hepatology. 2007;19(1):43-9. doi: 10.1097/01.meg.0000250586.61232.a3. PubMed PMID: WOS:000245597100007.

65. Nenna R, Tiberti C, Petrarca L, Lucantoni F, Mennini M, Luparia RPL, et al. The celiac iceberg: characterization of the disease in primary schoolchildren. Journal of pediatric gastroenterology and nutrition. 2013;56(4):416-21. doi: https://dx.doi.org/10.1097/MPG.0b013e31827b7f64.

66. Mejri K, Abida O, Kallel-Sellami M, Haddouk S, Laadhar L, Zarraa IR, et al. Spectrum of autoantibodies other than anti-desmoglein in pemphigus patients. Journal of the European Academy of Dermatology and Venereology. 2011;25(7):774-81. doi: 10.1111/j.1468-3083.2010.03859.x. PubMed PMID: WOS:000292478100004.

67. Robazzi TC, Adan LF, Pimentel K, Guimaraes I, Magalhaes J, Toralles MB, et al. Autoimmune endocrine disorders and coeliac disease in children and adolescents with juvenile idiopathic arthritis and rheumatic fever. Clinical and Experimental Rheumatology. 2013;31(2):310-7. PubMed PMID: WOS:000317086600023.

68. Nisihara R, Utiyama SR, Azevedo PM, Skare TL. Celiac disease screening in patients with scleroderma. Arquivos de gastroenterologia. 2011;48(2):163-4.

69. Moayeri H, Bahremand SH. Prevalence of Celiac disease in patients with Turner's syndrome. Acta Medica Iranica. 2005;43(4):287-90.

70. Shahmoradi Z, Najafian J, Naeini FF, Fahimipour F. Vitiligo and autoantibodies of celiac disease. International Journal of Preventive Medicine. 2013;4(2):200-3.
