# Supplementary material for: The accuracy of diagnostic indicators for coeliac disease: A systematic review and meta-analysis
Source: PLoS One. 2021 Oct 25;16(10):e0258501. doi: 10.1371/journal.pone.0258501 (PMC8545431; doi:10.1371/journal.pone.0258501)
Supplement: S2 Table — (DOCX) [file pone.0258501.s006.docx]

## Table S2: Study characteristics per indictor

| Study characteristics: Abdominal pain | | | | | | | | | |
| --- | --- | --- | --- | --- | --- | --- | --- | --- | --- |
| Age group | Study Design | Indicator details | Sample size | Sex (% female) | Control group | Reference standard | Care setting | Location | Reference |
| Adults | Case-control (DI) | Acute abdominal pain | 600 | NR | Healthy controls | Serology and only +ve pts biopsied | Secondary | NR | [1] |
| Adults | Case-control (DI) | Acute abdominal pain | 600 | 26-50% | Healthy controls | Serology and only +ve pts biopsied | Secondary | UK | [2] |
| Adults | Cohort | Abdominal pain | 3,196 | NR | People without abdominal pain | Double +ve for two antibodies | Community | US | [3] |
| Adults | Cohort | Abdominal pain | 2,976 | 51-75% | People without abdominal pain | Serology and only +ve pts biopsied | Community | Brazil | [4] |
| Adults | Nested case-control (CD) | Recurrent abdominal pain | 800 | 51-75% | People without recurrent abdominal pain | Double +ve for two antibodies | Primary and secondary | US | [5] |
| Adults | Nested case-control (CD) | Abdominal pain | 381 | 51-75% | People without abdominal pain | Double +ve for two antibodies | Secondary | US | [6] |
| Children | Case-control (DI) | Recurrent abdominal pain | 173 | 51-75% | Healthy controls | EMA positive | Primary | Canada | [7] |
| Children | Cohort | Any stomach pains | 4,327 | NR | Children without stomach pains | Double +ve for two antibodies | Community | UK | [8] |
| Children | Cohort | Abdominal pain | 18,672 | 51-75% | Children without abdominal pain | Serology and only +ve pts biopsied | Community | Turkey | [9] |
| Children | Cohort | Abdominal pain | 3,093 | 26-50% | Children without abdominal pain | tTG positive | Community | Netherlands | [10] |
| Children | Cohort | Abdominal pain | 3,715 | 26-50% | Children without abdominal pain | tTG positive | Community | Netherlands | [11] |
| Children | Cohort | Stomach aches | 9,918 | 51-75% | Children without stomach aches | tTG positive | Secondary | US | [12] |

| Study characteristics: Acid reflux symptoms | | | | | | | | | |
| --- | --- | --- | --- | --- | --- | --- | --- | --- | --- |
| Age group | Study Design | Indicator details | Sample size | Sex (% female) | Control group | Reference standard | Care setting | Location | Reference |
| Adults | Case-control (DI) | Dyspepsia | 640 | 51-75% | Healthy controls | Other | Secondary | Argentina | [13] |
| Adults | Case-control (DI) | Dyspepsia | 105 | NR | Healthy controls | All pts biopsied - no serology | NR | NR | [14] |
| Adults | Cohort | Dyspepsia | 3118 | NR | People without dyspepsia | Double +ve for two antibodies | Community | US | [3] |
| Adults | Cohort | Heartburn | 3847 | 51-75% | People without heartburn | Double +ve for two antibodies | Community | US | [15] |
| Adults | Cohort | Dyspepsia | 427 | 1-25% | Healthy controls | Serology and only +ve pts biopsied | Unclear | Mexico | [16] |
| Adults | Cohort | Heartburn | 1886 | NR | People without heartburn | Other | Primary | Finland | [17] |
| Adults | Nested case-control (CD) | Dyspepsia | 800 | 51-75% | People without dyspepsia | Double +ve for two antibodies | Primary and secondary | US | [5] |
| Adults | Nested case-control (DI) | Gastroesophageal reflux symptoms | 1000 | 51-75% | People without gastroesophageal reflux symptoms | Serology and all pts biopsied | Community | Sweden | [18] |
| Adults | Nested case-control (DI) | Dyspepsia | 112 | 26-50% | Healthy controls | tTG positive | Community | US | [19] |
| Mixed | Case-control (DI) | Functional dyspepsia | 257 | 26-50% | Healthy controls | Serology and only +ve pts biopsied | Secondary | Spain | [20] |

| Study characteristics: Anaemia | | | | | | | | | |
| --- | --- | --- | --- | --- | --- | --- | --- | --- | --- |
| Age group | Study Design | Indicator details | Sample size | Sex (% female) | Control group | Reference standard | Care setting | Location | Reference |
| Adults | Case-control (DI) | Low haemoglobin levels | 174 | NR | People with normal haemoglobin levels | tTG positive | Unclear | US | [21] |
| Adults | Case-control (DI) | Iron deficiency anaemia of obscure origin | 321 | NR | Healthy controls | Serology and only +ve pts biopsied | Secondary | India | [22] |
| Adults | Case-control (DI) | Iron deficiency anaemia of obscure origin | 196 | 51-75% | Healthy controls | Serology and only +ve pts biopsied | Secondary | Turkey | [23] |
| Adults | Case-control (DI) | Iron deficiency anaemia | 268 | 51-75% | Healthy controls | Serology and all pts biopsied | Secondary/community | Argentina | [24] |
| adults | Case-control (DI) | Iron deficiency anaemia | 97 | 51-75% | healthy controls | tTG positive | Secondary | Turkey | [25] |
| adults | Case-control (DI) | Pernicious anaemia | 165 | 51-75% | healthy controls | EMA positive | Unclear | Poland | [26] |
| adults | Cohort | Anaemia | 982 | NR | People without anaemia | Serology and only +ve pts biopsied | Primary | UK | [27] |
| adults | Cohort | Anaemia | 1197 | 26-50% | People without anaemia | Double +ve for two antibodies | Community | United Arab Emirates | [28] |
| adults | Cohort | Iron deficiency anaemia | 1200 | 62.8 | People without anaemia | Serology and only +ve pts biopsied | Primary | UK | [29] |
| adults | Cohort | Anaemia/Iron-deficiency anaemia | 527 | 51-75% | People without anaemia | Double +ve for two antibodies | Secondary | Malaysia | [30] |
| adults | Cohort | Anaemia | 5060 | 100% | People without anaemia | Double +ve for two antibodies | Secondary | Italy | [31] |
| adults | Nested case-control (CD) | Anaemia | 800 | 51-75% | People without anaemia | Double +ve for two antibodies | Primary and secondary | US | [5] |
| adults | Nested case-control (CD) | Anaemia | 381 | 51-75% | People without anaemia | Double +ve for two antibodies | Secondary | US | [6] |
| children | Case-control (DI) | Iron deficiency anaemia | 358 | 51-75% | healthy controls | Serology and only +ve pts biopsied | Secondary | Turkey | [32] |
| children | Case-control (DI) | Iron deficiency anaemia | 184 | 26-50% | healthy controls | Serology and only +ve pts biopsied | Secondary | Iran | [33] |
| children | Case-control (DI) | Iron deficiency anaemia | 304 | 51-75% | healthy controls | Serology and only +ve pts biopsied | Secondary | India | [34] |
| children | Cohort | Iron deficiency anaemia | 1263 | 26-50% | Children without IDA | tTG positive | Community | Turkey | [35] |

| Study characteristics: Arthritis | | | | | | | | | |
| --- | --- | --- | --- | --- | --- | --- | --- | --- | --- |
| Age group | Study Design | Indicator details | Sample size | Sex (% female) | Control group | Reference standard | Care setting | Location | Reference |
| Adults | Case-control (DI) | Rheumatoid arthritis | 220 | NR | Healthy controls | Serology and only +ve pts biopsied | Unclear | Italy | [36] |
| Adults | Case-control (DI) | Rheumatoid arthritis | 83 | NR | Healthy controls | EMA positive | Secondary | Ireland | [37] |
| Adults | Case-control (DI) | Rheumatoid arthritis | 182 | 76-100% | Healthy controls | Serology and only +ve pts biopsied | Secondary | Brazil | [38] |
| Adults | Case-control (DI) | Arthritis (PSA, RA or AS) | 237 | 26-50% | Healthy controls | Double +ve for two antibodies | Secondary | Italy | [39] |
| Adults | Case-control (DI) | Arthritis (PSA, RA or AS) | 275 | 26-50% | Healthy controls | tTG positive | Secondary | Italy | [40] |
| Adults | Cohort | Rheumatoid arthritis | 100 | NR | Healthy controls | Serology and only +ve pts biopsied | Secondary | US | [41] |
| Adults | Cohort | Rheumatoid arthritis | 6919 | 51-75% | People without rheumatoid arthritis | Double +ve for two antibodies | Community | Finland | [42] |
| Adults | Nested case-control (CD) | Rheumatoid arthritis | 800 | 51-75% | People without rheumatoid arthritis | Double +ve for two antibodies | Primary and secondary | US | [5] |
| Children | Case-control (DI) | Juvenile rheumatic diseases | 90 | 51-75% | Healthy controls | tTG positive | Secondary | Egypt | [43] |
| Children | Case-control (DI) | Juvenile idiopathic arthritis | 181 | 51-75% | Healthy controls | Serology and only +ve pts biopsied | Unclear | Turkey | [44] |
| Children | Case-control (DI) | Juvenile idiopathic arthritis | 205 | 51-75% | Healthy controls | Serology and only +ve pts biopsied | Secondary | Austria | [45] |
| Children | Case-control (DI) | Juvenile idiopathic arthritis | 309 | 51-75% | Healthy controls | Serology and only +ve pts biopsied | Secondary | Italy | [46] |
| Children | Cohort | Juvenile idiopathic arthritis | 70 | 26-50% | Healthy controls | Serology and only +ve pts biopsied | Secondary | Brazil | [47] |
| Mixed | Case-control (DI) | Juvenile idiopathic arthritis | 1025 | NR | Healthy controls | tTG positive | Secondary | US | [48] |
| Mixed | Case-control (DI) | Ankylosing spondylitis | 49 | 1-25% | Healthy controls | Serology and only +ve pts biopsied | Secondary | Turkey | [49] |

| Study characteristics: Bloating or abdominal distension | | | | | | | | | |
| --- | --- | --- | --- | --- | --- | --- | --- | --- | --- |
| Age group | Study Design | Indicator details | Sample size | Sex (% female) | Control group | Reference standard | Care setting | Location | Reference |
| Adults | Cohort | Bloating | 1830 | NR | People without bloating | Double +ve for two antibodies | Community | US | [3] |
| Adults | Cohort | Bloating | 3847 | 51-75% | People without bloating | Double +ve for two antibodies | Community | US | [15] |
| Adults | Cohort | Bloating | 1886 | NR | People without bloating | Other | Primary | Finland | [17] |
| Adults | Nested case-control (CD) | Bloating | 800 | 51-75% | People without bloating | Double +ve for two antibodies | Primary and secondary | US | [5] |
| Children | Cohort | Abdominal distension | 18598 | 51-75% | Children without abdominal distention | Serology and only +ve pts biopsied | Community | Turkey | [9] |
| Children | Cohort | Abdominal distension | 5733 | 26-50% | People without abdominal extension | Serology and only +ve pts biopsied | Community | Italy | [50] |

| Study characteristics: Constipation | | | | | | | | | |
| --- | --- | --- | --- | --- | --- | --- | --- | --- | --- |
| Age group | Study Design | Indicator details | Sample size | Sex (% female) | Control group | Reference standard | Care setting | Location | Reference |
| Adults | Cohort | Constipation | 3196 | NR | People without constipation | Double +ve for two antibodies | Community | US | [3] |
| Adults | Cohort | Constipation | 2976 | 51-75% | People without constipation | Serology and only +ve pts biopsied | Community | Brazil | [4] |
| Adults | Cohort | Constipation | 3847 | 51-75% | People without constipation | Double +ve for two antibodies | Community | US | [15] |
| Adults | Cohort | Constipation | 1886 | NR | People without constipation | Other | Primary | Finland | [17] |
| Adults | Nested case-control (CD) | Constipation | 800 | 51-75% | People without constipation | Double +ve for two antibodies | Primary and secondary | US | [5] |
| Children | Case-control (DI) | Chronic constipation | 1303 | 51-75% | Healthy controls | Serology and only +ve pts biopsied | Secondary | Turkey | [51] |
| Children | Cohort | Constipation | 4327 | NR | Children without constipation | Double +ve for two antibodies | Community | UK | [8] |
| Children | Cohort | Constipation | 18576 | 51-75% | Children without constipation | Serology and only +ve pts biopsied | Community | Turkey | [9] |
| Children | Cohort | Constipation | 3120 | 26-50% | Children without constipation | tTG positive | Community | Netherlands | [10] |
| Children | Cohort | Constipation | 3715 | 26-50% | Children without constipation | tTG positive | Community | Netherlands | [11] |
| Children | Cohort | Constipation | 9,918 | 51-75% | Children without constipation | tTG positive | Secondary | US | [12] |
| Children | Nested case-control (DI) | Functional constipation | 622 | NR | Children without functional gastrointestinal disorders | Serology and only +ve pts biopsied | Community | Colombia | [52] |

| Study characteristics: Dermatitis herpetiformis | | | | | | | | | |
| --- | --- | --- | --- | --- | --- | --- | --- | --- | --- |
| Age group | Study Design | Indicator details | Sample size | Sex (% female) | Control group | Reference standard | Care setting | Location | Reference |
| Adults | Case-control (DI) | Dermatitis herpetiformis | 150 | NR | healthy controls | Double +ve for two antibodies | Secondary | Poland | [53] |
| Adults | Case-control (DI) | Dermatitis herpetiformis | 46 | NR | healthy controls | Other | Secondary | Bulgaria | [54] |
| Adults | Nested case-control (CD) | Dermatitis herpetiformis | 381 | 51-75% | People without dermatitis herpetiformis | Double +ve for two antibodies | Secondary | US | [6] |
| Adults | Nested case-control (CD) | Dermatitis herpetiformis | 800 | 51-75% | People without dermatitis herpetiformis | Double +ve for two antibodies | Primary and secondary | US | [5] |
| Mixed | Case-control (DI) | Dermatitis herpetiformis | 52 | 26-50% | Controls | Serology and all pts biopsied | Secondary | Argentina | [55] |

| Study characteristics: Diarrhoea | | | | | | | | | |
| --- | --- | --- | --- | --- | --- | --- | --- | --- | --- |
| Age group | Study Design | Indicator details | Sample size | Sex (% female) | Control group | Reference standard | Care setting | Location | Reference |
| Adults | Cohort | Diarrhoea | 1197 | 26-50% | People without diarrhoea | Double +ve for two antibodies | Community | United Arab Emirates | [28] |
| Adults | Cohort | Diarrhoea | 3186 | NR | People without diarrhoea | Double +ve for two antibodies | Community | US | [3] |
| Adults | Cohort | Diarrhoea | 2976 | 51-75% | People without diarrhoea | Serology and only +ve pts biopsied | Community | Brazil | [4] |
| Adults | Cohort | Diarrhoea | 3847 | 51-75% | People without diarrhoea | Double +ve for two antibodies | Community | US | [15] |
| Adults | Cohort | Diarrhoea | 1886 | NR | People without diarrhoea | Other | Primary | Finland | [17] |
| Adults | Nested case-control (CD) | Diarrhoea | 800 | 51-75% | People without diarrhoea | Double +ve for two antibodies | Primary and secondary | US | [5] |
| Adults | Nested case-control (CD) | Diarrhoea | 381 | 51-75% | People without diarrhoea | Double +ve for two antibodies | Secondary | US | [6] |
| Children | Case-control (DI) | Diarrhoea | 1650 | 26-50% | healthy controls | Serology and only +ve pts biopsied | Secondary | Iran | [56] |
| Children | Cohort | Diarrhoea | 4327 | NR | Children without diarrhoea | Double +ve for two antibodies | Community | UK | [8] |
| Children | Cohort | Diarrhoea | 18602 | 51-75% | Children without diarrhoea | Serology and only +ve pts biopsied | Community | Turkey | [9] |
| Children | Cohort | Diarrhoea | 3015 | 26-50% | Children without diarrhoea | tTG positive | Community | Netherlands | [10] |
| Children | Cohort | Diarrhoea | 3715 | 26-50% | Children without diarrhoea | tTG positive | Community | Netherlands | [11] |
| Children | Cohort | Diarrhoea | 9,918 | 51-75% | Children without diarrhoea | tTG positive | Secondary | US | [12] |

| Study characteristics: Epilepsy | | | | | | | | | |
| --- | --- | --- | --- | --- | --- | --- | --- | --- | --- |
| Age group | Study Design | Indicator details | Sample size | Sex (% female) | Control group | Reference standard | Care setting | Location | Reference |
| adults | Case-control (DI) | Epilepsy | 1427 | 51-75% | healthy controls | tTG positive | Community | Finland | [57] |
| adults | Nested case-control (CD) | Epilepsy/ataxia | 800 | 51-75% | People without epilepsy/ataxia | Double +ve for two antibodies | Primary and secondary | US | [5] |
| children | Case-control (DI) | Epilepsy | 535 | 26-50% | healthy controls | Serology and only +ve pts biopsied | Secondary | Greece | [58] |
| children | Case-control (DI) | Epilepsy | 535 | 26-50% | healthy controls | Serology and only +ve pts biopsied | Secondary | Greece | [59] |
| children | Case-control (DI) | Epilepsy | 273 | 26-50% | healthy controls | Serology and only +ve pts biopsied | Secondary | Turkey | [60] |
| children | Case-control (DI) | Epilepsy | 190 | 26-50% | healthy controls | Serology and only +ve pts biopsied | Secondary | Turkey | [61] |
| children | Case-control (DI) | Epilepsy | 275 | 51-75% | healthy controls | Serology and only +ve pts biopsied | Secondary | Serbia | [62] |
| children | Case-control (DI) | Epilepsy | 572 | 26-50% | healthy controls | tTG positive | Secondary | Italy | [63] |
| children | Case-control (DI) | Epilepsy | 380 | 26-50% | healthy controls | Serology and only +ve pts biopsied | Secondary | Turkey | [64] |
| children | Case-control (DI) | Epilepsy | 1000 | 26-50% | healthy controls | Serology and only +ve pts biopsied | Secondary | Turkey | [65] |
| children | Case-control (DI) | Epilepsy | 70 | 26-50% | healthy controls | EMA positive | Secondary | Israel | [66] |
| mixed | Case-control (DI) | Epilepsy | 4660 | 26-50% | healthy controls | EMA positive | Secondary | Brazil | [67] |

| Study characteristics: Family history of CD | | | | | | | | | |
| --- | --- | --- | --- | --- | --- | --- | --- | --- | --- |
| Age group | Study Design | Indicator details | Sample size | Sex (% female) | Control group | Reference standard | Care setting | Location | Reference |
| Adults | Case-control (DI) | CD in family | 334 | NR | Healthy controls | tTG positive | Secondary | India | [68] |
| Adults | Cohort | FDRs | 1,197 | 26-50% | People without first degree relatives with CD | Double +ve for two antibodies | Community | United Arab Emirates | [28] |
| Adults | Cohort | CD in family | 527 | 51-75% | People without a family history of CD | Double +ve for two antibodies | Secondary | Malaysia | [30] |
| Adults | Nested case-control (DI) | FDRs | 2,128 | NR | People without CD in family | tTG positive | Community | US | [69] |
| Adults | Case-control (DI) | FDRs | 6,059 | 51-75% | Healthy controls | EMA positive | Community/secondary | US | [70] |
| Children | Cohort | CD in family | 3,768 | 51-75% | People without family history of CD | Serology and only +ve pts biopsied | Community | Cyprus | [71] |
| Children | Cohort | CD in family | 4,308 | 26-50% | Children without CD in family | tTG positive | Community | Netherlands | [10] |
| Children | Case-control (DI) | FDRs | 2,575 | 51-75% | Healthy controls | EMA positive | Community/secondary | US | [70] |
| Children | Cohort | FDRs | 9,973 | 51-75% | Children without a FDR with CD | tTG positive | Secondary | US | [12] |
| Mixed | Case-control (DI & CD) | FDRs | 114 | NR | Healthy controls | tTG positive | Unclear | Cuba | [72] |
| Mixed | Case-control (DI) | FDRs | 241 | 51-75% | Healthy controls | EMA positive | Secondary | Brazil | [73] |
| Mixed | Case-control (DI) | FDRs & SDRs | 333 | 51-75% | Healthy controls | Double +ve for two antibodies | Unclear | Brazil | [74] |
| Mixed | Case-control (DI) | FDRs & SDRs | 270 | 26-50% | Healthy controls | Double +ve for two antibodies | Unclear | Portugal | [75] |

| Study characteristics: Fracture | | | | | | | | | |
| --- | --- | --- | --- | --- | --- | --- | --- | --- | --- |
| Age group | Study Design | Indicator details | Sample size | Sex (% female) | Control group | Reference standard | Care setting | Location | Reference |
| adults | Case-control (DI) | Acute distal radius or ankle fracture | 597 | 76-100% | healthy controls | Serology and only +ve pts biopsied | secondary | Norway | [76] |
| adults | Case-control (DI) | Acute distal radius or ankle fracture | 228 | 76-100% | healthy controls | tTG positive | secondary | Norway | [77] |
| adults | Case-control (DI) | Hip fracture | 208 | 100% | Women without osteoporosis admitted for elective hip joint replacement | tTG positive | community | US | [78] |
| adults | Cohort | Non-traumatic fractures | 2121 | 51-75% | healthy controls | tTG positive | community | Australia | [79] |
| adults | Cohort | Fracture | 6480 | 100% | Women without fractures | tTG positive | community | Sweden | [80] |
| adults | Cohort | Vertebra fracture | 6919 | 51-75% | People without vertebra fracture | Double +ve for two antibodies | community | Finland | [42] |
| adults | Cohort | Fracture of the wrist | 7345 | 51-75% | People without wrist fracture | EMA positive | primary | UK | [81] |
| adults | Nested case-control (CD) | Fracture | 843 | 51-75% | People without fractures | Double +ve for two antibodies | community | US | [82] |

| Study characteristics: HLA | | | | | | | | | |
| --- | --- | --- | --- | --- | --- | --- | --- | --- | --- |
| Age group | Study Design | Indicator details | Sample size | Sex (% female) | Control group | Reference standard | Care setting | Location | Reference |
| Adults | Nested case-control (CD) | HLA DQ2 | 97 | NR | Healthy controls | Serology and all pts biopsied | Secondary | Sweden | [83] |
| Children | Case-control (CD) | HLA DQ2, HLA DQ8 or both | 1320 | 51-75% | Children without risk genotype | Serology and only +ve pts biopsied | Secondary | Sweden | [84] |
| Children | Cohort | HLA DQ2, HLA DQ8 or both | 2781 | 26-50% | People without risk genotype | tTG positive | Community | Netherlands | [85] |
| Children | Cohort | HLA DQ2, HLA DQ8 or both | 4308 | 26-50% | Children without risk genotype | tTG positive | Community | Netherlands | [10] |
| Children | Cohort | HLA DQ2.2, HLA DQ2.5 or HLA DQ8 | 3715 | 26-50% | Children without risk genotype | tTG positive | Community | Netherlands | [11] |
| Children | Cohort | HLA DR4-DQ8, DR3-DQ2 or both | 3627 | 51-75% | Children not carrying HLA DR3-DQ2 nor HLA DR4-DQ8 | Serology and only +ve pts biopsied | Community | Finland | [86] |
| Children | Nested case-control (DI) | HLA DQ2, HLA DQ8 or both | 3435 | 26-50% | New-borns without risk genotype | Serology and only +ve pts biopsied | Community | Sweden | [87] |
| Mixed | Case-control (DI & CD) | HLA DQ2 | 82 | NR | CD patients and healthy controls without risk genotype | tTG positive | Unclear | Cuba | [72] |
| Mixed | Case-control (CD) | HLA DQ2 | 101 | NR | People without HLA DQ2 | tTG positive | Secondary | Iraq | [88] |

| Study characteristics: Inflammatory bowel disease | | | | | | | | | |
| --- | --- | --- | --- | --- | --- | --- | --- | --- | --- |
| Age group | Study Design | Indicator details | Sample size | Sex (% female) | Control group | Reference standard | Care setting | Location | Reference |
| Adults | Case-control (DI) | Ulcerative colitis or Crohn's disease | 865 | 51-75% | Healthy controls | Serology and only +ve pts biopsied | Secondary | UK | [89] |
| Adults | Case-control (DI) | Ulcerative colitis or Crohn's disease | 955 | 51-75% | Healthy controls | Serology and only +ve pts biopsied | Secondary | UK | [90] |
| Adults | Case-control (DI) | Ulcerative colitis or Crohn's disease | 362 | 26-50% | Healthy controls | Double +ve for two antibodies | Secondary | Japan | [91] |
| Adults | Case-control (DI) | Ulcerative colitis or Crohn's disease | 290 | NR | Healthy controls | Serology and only +ve pts biopsied | Unclear | Italy | [36] |
| Children | Case-control (DI) | Ulcerative colitis or Crohn's disease | 328 | 26-50% | Healthy controls | tTG positive | Secondary | NR | [92] |
| Mixed | Case-control (DI) | Ulcerative colitis | 86 | 51-75% | Healthy controls | EMA positive | Secondary | Estonia | [93] |

| Study characteristics: irritable bowel syndrome | | | | | | | | | |
| --- | --- | --- | --- | --- | --- | --- | --- | --- | --- |
| Age group | Study Design | Indicator details | Sample size | Sex (% female) | Control group | Reference standard | Care setting | Location | Reference |
| Adults | Case-control (DI) | Irritable bowel syndrome | 233 | 76-100% | healthy controls | tTG positive | Secondary | Iran | [94] |
| Adults | Case-control (DI) | Irritable bowel syndrome | 200 | 51-75% | healthy controls | tTG positive | Secondary | Poland | [95] |
| Adults | Case-control (DI) | Irritable bowel syndrome | 1,064 | 76-100% | healthy controls | Double +ve for two antibodies | Secondary | US | [96] |
| Adults | Case-control (DI) | Irritable bowel syndrome | 950 | NR | healthy controls | Serology and only +ve pts biopsied | Secondary | US | [97] |
| Adults | Case-control (DI) | Irritable bowel syndrome | 68 | 51-75% | healthy controls | tTG positive | NR | Poland | [98] |
| Adults | Case-control (DI) | Irritable bowel syndrome | 492 | 51-75% | Controls who underwent colonoscopy examination for colorectal cancer (CRC) screening or polyp surveillance. | tTG positive | Secondary | China | [99] |
| Adults | Case-control (DI) | Irritable bowel syndrome | 1,121 | 76-100% | Healthy controls | Double +ve for two antibodies | Secondary | US | [100] |
| Adults | Case-control (DI) | Irritable bowel syndrome | 800 | 76-100% | Healthy controls | Serology and only +ve pts biopsied | Secondary | Mexico | [101] |
| Adults | Case-control (DI) | Irritable bowel syndrome | 600 | 51-75% | Healthy controls | Serology and only +ve pts biopsied | Secondary | UK | [102] |
| Adults | Case-control (DI) | Irritable bowel syndrome | 678 | 76-100% | Healthy controls | Serology and only +ve pts biopsied | Secondary | Mexico | [103] |
| Adults | Case-control (DI) | Irritable bowel syndrome | 758 | 51-75% | Healthy controls | Serology and only +ve pts biopsied | Secondary | China | [104] |
| Adults | Case-control (DI) | Irritable bowel syndrome | 509 | 26-50% | Healthy controls | tTG positive | Secondary | Saudi Arabia | [105] |
| Adults | Cohort | Irritable bowel syndrome | 1,200 | 62.8 | People without irritable bowel syndrome | Serology and only +ve pts biopsied | Primary | UK | [29] |
| Adults | Cohort | Irritable bowel syndrome | 3,196 | NR | People without irritable bowel disease | Double +ve for two antibodies | Community | US | [3] |
| Adults | Nested case-control (CD) | Irritable bowel syndrome | 800 | 51-75% | People without IBS | Double +ve for two antibodies | Primary and secondary | USA | [5] |
| Adults | Nested case-control (CD) | Irritable bowel syndrome | 381 | 51-75% | People without irritable bowel syndrome | Double +ve for two antibodies | Secondary | US | [6] |
| Adults | Nested case-control (DI) | Irritable bowel syndrome | 128 | 26-50% | Healthy controls | tTG positive | Community | US | [19] |
| Children | Cohort | Functional gastrointestinal disorder | 5,268 | NR | Healthy controls | Serology and only +ve pts biopsied | Community | Sweden | [106] |

| Study characteristics: Chronic liver disease | | | | | | | | | |
| --- | --- | --- | --- | --- | --- | --- | --- | --- | --- |
| Age group | Study Design | Indicator details | Sample size | Sex (% female) | Control group | Reference standard | Care setting | Location | Reference |
| Adults | Case-control (DI) | Primary biliary cirrhosis | 168 | NR | Healthy controls | Serology and only +ve pts biopsied | Unclear | Italy | [36] |
| Adults | Case-control (DI) | Primary biliary cirrhosis | 162 | 76-100% | Healthy controls | Serology and only +ve pts biopsied | Secondary | Greece | [107] |
| Adults | Case-control (DI) | Chronic hepatitis C | 395 | 26-50% | Healthy controls | Double +ve for two antibodies | Secondary | Italy | [108] |
| Adults | Case-control (DI) | Chronic hepatitis C | 275 | 26-50% | Healthy controls | Serology and only +ve pts biopsied | Secondary | USA | [109] |
| Adults | Case-control (DI) | ALD, HCV, PBC, PSC, CH | 2,002 | 26-50% | Healthy controls | Serology and only +ve pts biopsied | Secondary | Sweden | [110] |
| Adults | Case-control (DI) | Abnormal liver function tests | 250 | 1-25% | Healthy controls | tTG positive | Secondary | China | [111] |
| Adults | Cohort | Hepatitis | 1,197 | 26-50% | People without hepatitis | Double +ve for two antibodies | Community | United Arab Emirates | [28] |
| Adults | Cohort | Hepatic diseases | 527 | 51-75% | People without hepatic diseases | Double +ve for two antibodies | Secondary | Malaysia | [30] |
| Adults | Nested case-control (CD) | Unexplained abnormal AST/ALT | 800 | 51-75% | People without unexplained abnormal AST/ALT | Double +ve for two antibodies | Primary and secondary | USA | [5] |
| Children | Case-control (DI) | Autoimmune hepatitis | 46 | 26-50% | Healthy controls | Serology and only +ve pts biopsied | Secondary | Egypt | [112] |
| Children | Case-control (DI) | Autoimmune hepatitis | 122 | NR | Healthy controls | Serology and only +ve pts biopsied | NR | Romania | [113] |
| Mixed | Case-control (DI) | HCV, HBV, AIH, PBC, PSC, NAFLD-ALD, NAFLD, and others | 2,084 | 51-75% | Healthy controls | Serology and only +ve pts biopsied | Secondary | Greece | [114] |
| Mixed | Case-control (DI) | Autoimmune hepatitis | 167 | NR | Healthy controls | Other | Secondary | Italy | [115] |
| Mixed | Case-control (DI) | Chronic hepatitis C | 267 | 26-50% | Healthy controls | Serology and only +ve pts biopsied | Secondary | Spain | [20] |
| Mixed | Case-control (DI) | Chronic hepatitis C | 220 | NR | Healthy controls | Other | Secondary | Italy | [115] |

| Study characteristics: Migraine | | | | | | | | | |
| --- | --- | --- | --- | --- | --- | --- | --- | --- | --- |
| Age group | Study Design | Indicator details | Sample size | Sex (% female) | Control group | Reference standard | Care setting | Location | Reference |
| Adults | Case-control (DI) | Migraine | 326 | 51-75% | Healthy controls | Serology and only +ve pts biopsied | Secondary | Italy | [116] |
| Children | Case-control (DI) | Migraine | 257 | 51-75% | Healthy controls | Serology and only +ve pts biopsied | Secondary | Turkey | [117] |
| Children | Case-control (DI) | Migraine | 220 | 51-75% | Healthy controls | tTG positive | Secondary | Turkey | [118] |
| Children | Case-control (DI) | Migraine | 1,600 | 26-50% | Healthy controls | Serology and only +ve pts biopsied | Secondary | Iran | [119] |
| Children | Case-control (DI) | Migraine headaches | 75 | 26-50% | Healthy controls | EMA positive | Secondary | Israel | [66] |

| Study characteristics: Multiple sclerosis | | | | | | | | | |
| --- | --- | --- | --- | --- | --- | --- | --- | --- | --- |
| Age group | Study Design | Indicator details | Sample size | Sex (% female) | Control group | Reference standard | Care setting | Location | Reference |
| adults | Case-control (DI) | Multiple sclerosis | 68 | NR | healthy controls | Serology and only +ve pts biopsied | unclear | Iran | [120] |
| adults | Case-control (DI) | Multiple sclerosis | 417 | 51-75% | healthy controls | tTG positive | secondary | Italy | [121] |
| adults | Case-control (DI) | Multiple sclerosis | 195 | 76-100% | healthy controls | tTG positive | secondary | Spain | [122] |
| adults | Case-control (DI) | Multiple sclerosis | 185 | 51-75% | healthy controls | Double +ve for two antibodies | secondary | Sweden | [123] |
| mixed | Case-control (DI) | Multiple sclerosis | 221 | 51-75% | healthy controls | tTG positive | secondary | Iran | [124] |

| Study characteristics: Osteoporosis | | | | | | | | | |
| --- | --- | --- | --- | --- | --- | --- | --- | --- | --- |
| Age group | Study Design | Indicator details | Sample size | Sex (% female) | Control group | Reference standard | Care setting | Location | Reference |
| Adults | Case-control (DI) | Osteoporosis | 197 | 76-100% | healthy controls | Serology and only +ve pts biopsied | Secondary | Brazil | [125] |
| Adults | Case-control (DI) | Osteoporosis | 560 | 51-75% | healthy controls | Serology and only +ve pts biopsied | Secondary | Iran | [126] |
| Adults | Case-control (DI) | Osteoporosis | 840 | 76-100% | healthy controls | Serology and only +ve pts biopsied | Secondary | USA | [127] |
| Adults | Case-control (DI) | Osteoporosis | 1,414 | 26-50% | healthy controls | Double +ve for two antibodies | Secondary | Czechia | [128] |
| Adults | Cohort | Osteoporosis | 2,121 | 51-75% | People without osteoporosis | tTG positive | Community | Australia | [79] |
| Adults | Cohort | Osteoporosis | 6,480 | 100% | Women without osteoporosis | tTG positive | Community | Sweden | [80] |
| Adults | Nested case-control (CD) | Osteoporosis | 800 | 51-75% | People without osteoporosis | Double +ve for two antibodies | Primary and secondary | USA | [5] |
| Adults | Nested case-control (CD) | Osteoporosis | 843 | 51-75% | People without osteoporosis | Double +ve for two antibodies | Community | US | [82] |
| Mixed | Case-control (CD) | Osteoporosis | 6,963 | NR | Patients without osteoporosis | Other | Secondary | USA | [129] |

| Study characteristics: Psoriasis | | | | | | | | | |
| --- | --- | --- | --- | --- | --- | --- | --- | --- | --- |
| Age group | Study Design | Indicator details | Sample size | Sex (% female) | Control group | Reference standard | Care setting | Location | Reference |
| Adults | Case-control (DI) | Psoriasis | 482 | 51-75% | General population | Serology and only +ve pts biopsied | Primary | Italy | [130] |
| Adults | Case-control (DI) | Psoriasis | 87 | 51-75% | Healthy controls | Serology and only +ve pts biopsied | Secondary | Turkey | [131] |
| Adults | Case-control (DI) | Chronic plaque psoriasis | 160 | 26-50% | Healthy controls | tTG positive | Unclear | India | [132] |
| Mixed | Case-control (DI) | Psoriasis | 200 | 26-50% | Healthy controls | tTG positive | Secondary | Italy | [133] |
| Mixed | Case-control (DI) | Psoriasis | 82 | 26-50% | Healthy controls | tTG positive | Secondary | Egypt | [134] |
| Mixed | Case-control (DI) | Psoriasis | 116 | 26-50% | Healthy controls | tTG positive | Secondary | India | [135] |

| Study characteristics: Systemic lupus erythematosus | | | | | | | | | |
| --- | --- | --- | --- | --- | --- | --- | --- | --- | --- |
| Age group | Study Design | Indicator details | Sample size | Sex (% female) | Control group | Reference standard | Care setting | Location | Reference |
| Adults | Case-control (DI) | Systemic lupus erythematosus | 220 | 76-100% | healthy controls | Serology and only +ve pts biopsied | secondary | Italy | [136] |
| Adults | Case-control (DI) | Systemic lupus erythematosus | 220 | NR | Healthy controls | Serology and only +ve pts biopsied | Unclear | Italy | [36] |
| Adults | Case-control (DI) | Systemic lupus erythematosus | 76 | NR | Healthy controls | EMA positive | Secondary | Ireland | [37] |
| Adults | Case-control (DI) | Systemic lupus erythematosus | 297 | 76-100% | Healthy controls | Double +ve for two antibodies | Secondary | Brazil | [137] |
| Adults | Cohort | Systemic lupus erythematosus | 100 | NR | Healthy controls | Serology and only +ve pts biopsied | Secondary | US | [41] |
| Children | Case-control (DI) | Systemic lupus erythematosus | 91 | 76-100% | Healthy controls | Serology and only +ve pts biopsied | Unclear | Turkey | [138] |

| Study characteristics: Subfertility or recurrent pregnancy loss | | | | | | | | | |
| --- | --- | --- | --- | --- | --- | --- | --- | --- | --- |
| Age group | Study Design | Indicator details | Sample size | Sex (% female) | Control group | Reference standard | Care setting | Location | Reference |
| Adults | Case-control (DI) | Recurrent miscarriages or implantation failure | 528 | 100% | Healthy controls | tTG positive | Secondary | Czech Republic | [139] |
| Adults | Case-control (DI) | Unexplained infertility or recurrent miscarriages | 342 | 100% | Healthy controls | tTG positive | Secondary | Mexico | [140] |
| Adults | Case-control (DI) | Recurrent miscarriages or implantation failure | 279 | 100% | Healthy controls | Serology and only +ve pts biopsied | Secondary | Spain | [141] |
| Adults | Case-control (DI) | Unexplained infertility | 535 | 100% | Healthy controls | tTG positive | Secondary | India | [142] |
| Adults | Case-control (DI) | Recurrent pregnancy loss | 808 | 100% | Healthy controls | tTG positive | Secondary | US | [143] |
| Adults | Case-control (DI) | Recurrent pregnancy loss | 409 | 100% | Healthy controls | tTG positive | Secondary | India | [142] |
| Adults | Case-control (DI) | Recurrent pregnancy loss | 86 | 100% | Healthy controls | tTG positive | Secondary | Turkey | [144] |
| Adults | Case-control (DI) | Unexplained infertility | 402 | 100% | Healthy controls | Serology and only +ve pts biopsied | Secondary | Israel | [145] |
| Adults | Case-control (DI) | Recurrent pregnancy loss | 232 | 100% | Healthy controls | tTG positive | Secondary | US | [146] |
| Adults | Case-control (DI) | Infertility | 400 | 100% | Healthy controls | Serology and only +ve pts biopsied | Secondary | Italy | [147] |
| Adults | Case-control (DI) | Infertility | 297 | 100% | Healthy controls | tTG positive | Unclear | Iran | [148] |
| Adults | Case-control (DI) | Infertility | 1675 | 26-50% | Healthy controls | Double +ve for two antibodies | Secondary | Czech Republic | [128] |
| Adults | Cohort | Spontaneous abortion | 5060 | 100% | Women without spontaneous abortion | Double +ve for two antibodies | Secondary | Italy | [31] |
| Adults | Nested case-control (CD) | Infertility | 800 | 51-75% | People without infertility | Double +ve for two antibodies | Primary and secondary | US | [5] |
| Adults | Nested case-control (CD) | Previous abortion | 619 | 100% | People without previous abortion | Double +ve for two antibodies | Community | US | [149] |
| Adults | Nested case-control (DI) | Previous miscarriage | 218 | 100% | Healthy controls | Serology and only +ve pts biopsied | Secondary | Italy | [150] |

| Study characteristics: Type 1 Diabetes | | | | | | | | | |
| --- | --- | --- | --- | --- | --- | --- | --- | --- | --- |
| Age group | Study Design | Indicator details | Sample size | Sex (% female) | Control group | Reference standard | Care setting | Location | Reference |
| Adults | Case-control (DI) | Type 1 Diabetes | 600 | 51-75% | Healthy controls | Serology and only +ve pts biopsied | Secondary | Egypt | [151] |
| Adults | Case-control (DI) | Type 1 Diabetes | 177 | 51-75% | Healthy controls | Serology and only +ve pts biopsied | Secondary | Italy | [152] |
| Adults | Case-control (DI) | Type 1 Diabetes | 130 | 26-50% | Healthy controls | Serology and only +ve pts biopsied | Secondary | Turkey | [153] |
| Adults | Case-control (DI) | Type 1 Diabetes | 180 | 51-75% | Healthy controls | Serology and only +ve pts biopsied | Secondary | Turkey | [154] |
| Adults | Case-control (DI) | Type 1 Diabetes | 1,680 | 26-50% | Healthy controls | Serology and only +ve pts biopsied | Secondary | Israel | [155] |
| Adults | Case-control (DI) | Type 1 Diabetes | 2,200 | 51-75% | Healthy controls | Serology and only +ve pts biopsied | Secondary | UK | [156] |
| Adults | Case-control (DI) | Type 1 Diabetes | 346 | NR | Healthy controls | tTG positive | Secondary | India | [157] |
| Adults | Case-control (DI) | Type 1 Diabetes | 280 | 51-75% | Healthy controls | tTG positive | Secondary | China | [158] |
| Adults | Cohort | Type 1 Diabetes | 527 | 51-75% | People without type 1 diabetes | Double +ve for two antibodies | Secondary | Malaysia | [30] |
| Adults | Cohort | Type 1 Diabetes | 6,919 | 51-75% | People without type 1 diabetes | Double +ve for two antibodies | Community | Finland | [42] |
| Adults | Nested case-control (CD) | Type 1 Diabetes | 800 | 51-75% | People without type 1 diabetes | Double +ve for two antibodies | Primary and secondary | USA | [5] |
| Children | Case-control (DI) | Type 1 Diabetes | 1,750 | 26-50% | Children without type 1 diabetes | Other | Secondary | Egypt | [159] |
| Children | Case-control (DI) | Type 1 Diabetes | 971 | 26-50% | Healthy controls | Double +ve for two antibodies | Community | Sweden | [160] |
| Children | Case-control (DI) | Type 1 Diabetes | 1,363 | 26-50% | Healthy controls | Double +ve for two antibodies | Community | Denmark | [160] |
| Children | Case-control (DI) | Type 1 Diabetes | 335 | 26-50% | Healthy controls | Serology and only +ve pts biopsied | Secondary | USA | [161] |
| Children | Case-control (DI) | Type 1 Diabetes | 209 | 26-50% | Healthy controls | Serology and only +ve pts biopsied | Secondary | Brazil | [162] |
| Children | Case-control (DI) | Type 1 Diabetes | 246 | 51-75% | Healthy controls | Serology and only +ve pts biopsied | Secondary | Serbia | [163] |
| Children | Case-control (DI) | Type 1 Diabetes | 394 | NR | Healthy controls | tTG positive | Unclear | US | [164] |
| Children | Case-control (DI) | Type 1 Diabetes | 272 | 26-50% | Healthy controls | tTG positive | Secondary | Romania | [165] |
| Children | Case-control (DI) | Type 1 Diabetes | 265 | 26-50% | Healthy controls | Serology and only +ve pts biopsied | Secondary | Sweden | [166] |
| Children | Case-control (DI) | Type 1 Diabetes | 197 | NR | Healthy controls | tTG positive | Secondary | Colombia | [167] |
| Children | Case-control (DI) | Type 1 Diabetes | 74 | 26-50% | Healthy controls | Serology and only +ve pts biopsied | Secondary | Turkey | [168] |
| Children | Case-control (DI) | Type 1 Diabetes | 446 | 51-75% | Healthy controls | tTG positive | Secondary | Colombia | [169] |
| Mixed | Case-control (DI) | Type 1 Diabetes | 4,491 | 26-50% | Healthy controls | Serology and only +ve pts biopsied | Secondary | Italy | [170] |
| Mixed | Case-control (DI) | Type 1 Diabetes | 219 | 26-50% | Healthy controls | tTG positive | Secondary | Sri Lanka | [171] |
| Mixed | Case-control (DI) | Type 1 Diabetes | 347 | 51-75% | Healthy controls | tTG positive | Secondary | Germany | [172] |
| Mixed | Case-control (DI) | Type 1 Diabetes | 196 | NR | Healthy controls | tTG positive | Secondary | India | [173] |
| Mixed | Case-control (DI) | Type 1 Diabetes | 151 | 51-75% | Healthy controls | Serology and only +ve pts biopsied | Secondary | Turkey | [174] |
| Mixed | Case-control (DI) | Type 1 Diabetes | 500 | 26-50% | Healthy controls | tTG positive | Secondary | Italy | [175] |
| Mixed | Case-control (DI) | Type 1 Diabetes | 250 | 51-75% | Healthy controls | tTG positive | Secondary | Iran | [176] |
| Mixed | Case-control (DI) | Type 1 Diabetes | 120 | NR | Healthy controls | Serology and only +ve pts biopsied | Secondary | Iran | [177] |

| Study characteristics: Type 2 Diabetes | | | | | | | | | |
| --- | --- | --- | --- | --- | --- | --- | --- | --- | --- |
| Age group | Study Design | Indicator details | Sample size | Sex (% female) | Control group | Reference standard | Care setting | Location | Reference |
| Adults | Case-control (DI) | Type 2 Diabetes | 250 | 51-75% | Healthy controls | Serology and only +ve pts biopsied | Secondary | Turkey | [178] |
| Adults | Case-control (DI) | Type 2 Diabetes | 113 | 51-75% | Healthy controls | tTG positive | Secondary | Poland | [179] |
| Adults | Case-control (DI) | Type 2 Diabetes | 247 | 51-75% | Healthy controls | tTG positive | Secondary | China | [158] |
| Adults | Cohort | Type 2 Diabetes | 6919 | 51-75% | People without diabetes | Double +ve for two antibodies | Community | Finland | [42] |
| Mixed | Case-control (DI) | Type 2 Diabetes | 338 | NR | Healthy controls | tTG positive | Secondary | India | [173] |
| Mixed | Case-control (DI) | Type 2 Diabetes | 332 | 26-50% | Healthy controls | tTG positive | Secondary | Italy | [175] |

| Study characteristics: Thyroid disease | | | | | | | | | |
| --- | --- | --- | --- | --- | --- | --- | --- | --- | --- |
| Age group | Study Design | Indicator details | Sample size | Sex (% female) | Control group | Reference standard | Care setting | Location | Reference |
| Adults | Case-control (DI) | Thyroid autoimmunity | 1,337 | NR | Healthy controls | Serology and only +ve pts biopsied | Secondary | Italy | [180] |
| Adults | Case-control (DI) | Thyroid autoimmunity | 814 | NR | Healthy controls | Serology and only +ve pts biopsied | Secondary | Italy | [180] |
| Adults | Case-control (DI) | Hashimoto's thyroiditis | 82 | 100% | Healthy controls | tTG positive | Secondary | Iran | [181] |
| Adults | Case-control (DI) | Graves' disease | 354 | 76-100% | Healthy controls | Serology and only +ve pts biopsied | Secondary | Poland | [182] |
| Adults | Case-control (DI) | Hashimoto's thyroiditis or Graves' disease | 4,172 | 76-100% | Healthy controls | Serology and only +ve pts biopsied | Secondary | Italy | [183] |
| Adults | Case-control (DI) | Graves' disease | 124 | NR | Healthy controls | EMA positive | Secondary | Ireland | [37] |
| Adults | Case-control (DI) | Graves' disease | 226 | 76-100% | Healthy controls | Double +ve for two antibodies | Secondary | UK | [184] |
| Adults | Case-control (DI) | Thyroid autoimmunity | 255 | 76-100% | Healthy controls | Serology and only +ve pts biopsied | Secondary | Turkey | [185] |
| Adults | Case-control (DI) | Hashimoto's thyroiditis | 470 | NR | Healthy controls | Serology and only +ve pts biopsied | Secondary | Italy | [186] |
| Adults | Case-control (DI) | Autoimmune and non-autoimmune thyroid disease | 318 | 51-75% | Healthy controls | tTG positive | Secondary | China | [158] |
| Adults | Cohort | Thyroid disorder | 1,197 | 26-50% | People without thyroid disorder | Double +ve for two antibodies | Community | United Arab Emirates | [28] |
| Adults | Cohort | Thyroid disorder | 527 | 51-75% | People without thyroid disorder | Double +ve for two antibodies | Secondary | Malaysia | [30] |
| Adults | Cohort | Thyroid disorder | 4,633 | 26-50% | Healthy controls | tTG positive | Community | Germany | [187] |
| Adults | Cohort | Thyroid disorder | 7,339 | 51-75% | People without thyroid disease | EMA positive | Primary | UK | [81] |
| Adults | Nested case-control (CD) | Thyroiditis, hypo-, or hyperthyroidism | 800 | 51-75% | People without thyroid disease | Double +ve for two antibodies | Primary and secondary | US | [5] |
| Adults | Nested case-control (DI) | TPOAbs | 682 | NR | Healthy controls | tTG positive | Community | India | [188] |
| Children | Case-control (DI) | Thyroid autoimmunity | 132 | 51-75% | Healthy controls | Serology and only +ve pts biopsied | Unclear | Turkey | [189] |
| Children | Case-control (DI) | Thyroid autoimmunity | 204 | 51-75% | Healthy controls | Serology and only +ve pts biopsied | Secondary | Turkey | [190] |
| Children | Case-control (DI) | Thyroid autoimmunity | 134 | NR | Healthy controls | Serology and only +ve pts biopsied | NR | Romania | [113] |
| Children | nested case control (based on CD) | TPOAbs | 2030 | 26-50% | Healthy controls | Serology and only +ve pts biopsied | Community | Sweden | [191] |
| Children | Nested case-control (DI) | TPOAbs | 472 | NR | Healthy controls | tTG positive | Community | India | [188] |
| Mixed | Case-control (DI) | Free T4 or TSH | 77 | 51-75% | Healthy controls | Double +ve for two antibodies | Secondary | Iraq | [192] |
| Mixed | Case-control (DI) | Thyroid autoimmunity | 652 | 76-100% | Healthy controls | tTG positive | Community | Brazil | [193] |

| Study characteristics: Vomiting and nausea | | | | | | | | | |
| --- | --- | --- | --- | --- | --- | --- | --- | --- | --- |
| Age group | Study Design | Indicator details | Sample size | Sex (% female) | Control group | Reference standard | Care setting | Location | Reference |
| Adults | Cohort | Vomiting | 2843 | NR | People without vomiting | Double +ve for two antibodies | Community | US | [3] |
| Adults | Cohort | Nausea | 3847 | 51-75% | People without nausea | Double +ve for two antibodies | Community | US | [15] |
| Adults | Cohort | Vomiting | 3847 | 51-75% | People without vomiting | Double +ve for two antibodies | Community | US | [15] |
| Children | Cohort | Vomiting | 4327 | NR | Children without vomiting | Double +ve for two antibodies | Community | UK | [8] |
| Children | Cohort | Vomiting | 18593 | 51-75% | Children without vomiting | Serology and only +ve pts biopsied | Community | Turkey | [9] |
| Children | Cohort | Nausea | 1562 | 26-50% | Children without nausea (after eating) | tTG positive | Community | Netherlands | [10] |
| Children | Cohort | Vomiting | 9918 | 51-75% | Children without vomiting | tTG positive | Secondary | US | [12] |

| Study characteristics: Weight loss | | | | | | | | | |
| --- | --- | --- | --- | --- | --- | --- | --- | --- | --- |
| Age group | Study Design | Indicator details | Sample size | Sex (% female) | Control group | Reference standard | Care setting | Location | Reference |
| Adults | Cohort | Weight loss | 1960 | NR | People without weight loss | Double +ve for two antibodies | Community | US | [3] |
| Adults | Nested case-control (CD) | Weight loss | 800 | 51-75% | People without unexplained weight loss | Double +ve for two antibodies | Primary and secondary | USA | [5] |
| Adults | Nested case-control (CD) | Weight loss | 381 | 51-75% | People without weight loss | Double +ve for two antibodies | Secondary | USA | [6] |
| Children | Cohort | Weight loss | 18680 | 51-75% | Children without weight loss | Serology and only +ve pts biopsied | Community | Turkey | [9] |
| Children | Cohort | Weight loss | 9918 | 51-75% | Children without weight loss | tTG positive | Secondary | US | [12] |

## References

1. Hopper AD, Azmy IA, Rahman N, Hurlstone DP, Leeds JS, George RR, et al. Association of adult coeliac disease with surgical abdominal pain: a case controlled study in patients referred to secondary care. Gastroenterology. 2005;128(4):A255-A. PubMed PMID: WOS:000228619302064.

2. Sanders DS, Hopper AD, Azmy IAF, Rahman N, Hurlstone DP, Leeds JS, et al. Association of adult Celiac disease with surgical abdominal pain - A case-control study in patients referred to secondary care. Annals of Surgery. 2005;242(2):201-7. doi: 10.1097/01.sla.0000171301.35513.cf. PubMed PMID: WOS:000230858000008.

3. Choung RS, Rubio-Tapia A, Lahr BD, Kyle RA, Camilleri MJ, Locke GR, et al. Evidence Against Routine Testing of Patients With Functional Gastrointestinal Disorders for Celiac Disease: A Population-based Study. Clinical Gastroenterology and Hepatology. 2015;13(11):1937-43. doi: http://dx.doi.org/10.1016/j.cgh.2015.05.014.

4. Oliveira RP, Sdepanian VL, Barreto JA, Cortez AJP, Carvalho FO, Bordin JO, et al. High prevalence of celiac disease in Brazilian blood donor volunteers based on screening by IgA antitissue transglutaminase antibody. European Journal of Gastroenterology & Hepatology. 2007;19(1):43-9. doi: 10.1097/01.meg.0000250586.61232.a3. PubMed PMID: WOS:000245597100007.

5. Hujoel IA, Van Dyke CT, Brantner T, Larson J, King KS, Sharma A, et al. Natural history and clinical detection of undiagnosed coeliac disease in a North American community. Alimentary Pharmacology and Therapeutics. 2018;47(10):1358-66. doi: http://dx.doi.org/10.1111/apt.14625.

6. Godfrey JD, Brantner TL, Brinjikji W, Christensen KN, Brogan DL, Van Dyke CT, et al. Morbidity and mortality among older individuals with undiagnosed celiac disease. Gastroenterology. 2010;139(3):763-9. doi: http://dx.doi.org/10.1053/j.gastro.2010.05.041.

7. Fitzpatrick KP, Sherman PA, Ipp T, Saunders T, Macarthur C. Screening for celiac disease in children with recurrent abdominal pain. Journal of Pediatric Gastroenterology and Nutrition. 2001;33(3):250-2. doi: 10.1097/00005176-200109000-00004. PubMed PMID: WOS:000171467800004.

8. Bingley PJ, Williams AJK, Norcross AJ, Unsworth DJ, Lock RJ, Ness AR, et al. Undiagnosed coeliac disease at age seven: Population based prospective birth cohort study. British Medical Journal. 2004;328(7435):322-3.

9. Dalgic B, Sari S, Ozcan B, Basturk B, Ensari A, Egritas O, et al. The evaluation of factors and symptoms related to celiac disease in Turkish children. Turk Pediatri Arsivi. 2011;46(4):314-21. doi: http://dx.doi.org/10.4274/tpa.672.

10. Jansen MAE, Kiefte-de Jong JC, Gaillard R, Escher JC, Hofman A, Jaddoe VWV, et al. Growth trajectories and bone mineral density in anti-tissue transglutaminase antibody-positive children: The generation R study. Clinical Gastroenterology and Hepatology. 2015;13(5):913-20. doi: http://dx.doi.org/10.1016/j.cgh.2014.09.032.

11. Wahab RJ, Beth SA, Derks IPM, Jansen PW, Moll HA, Kiefte-De Jong JC. Celiac disease autoimmunity and emotional and behavioral problems in childhood. Pediatrics. 2019;144(4):e20183933. doi: http://dx.doi.org/10.1542/peds.2018-3933.

12. Stahl MG, Rasmussen CG, Dong F, Waugh K, Norris JM, Baxter J, et al. Mass Screening for Celiac Disease: The Autoimmunity Screening for Kids Study. American Journal of Gastroenterology. 2021;116(1):180-7. doi: 10.14309/ajg.0000000000000751. PubMed PMID: WOS:000607726100027.

13. Lasa J, Spallone L, Gandara S, Chaar E, Berman S, Zagalsky D. Celiac disease prevalence is not increased in patients with functional dyspepsia. Arquivos de Gastroenterologia. 2017;54(1):37-40. doi: http://dx.doi.org/10.1590/S0004-2803.2017v54n1-07.

14. Lecleire S, Di Fiore F, Antonietti M, Savoye G, Lerebours E, Ducrotte P. Endoscopic markers for screening of villous atrophy in patients with dyspepsia or high-risk of celiac disease: a prospective controlled study. Gastroenterology. 2005;128(4):A257-A. PubMed PMID: WOS:000228619302071.

15. Katz KD, Rashtak S, Lahr BD, Melton LJ, Krause PK, Maggi K, et al. Screening for celiac disease in a North American population: Sequential serology and gastrointestinal symptoms. American Journal of Gastroenterology. 2011;106(7):1333-9. doi: http://dx.doi.org/10.1038/ajg.2011.21.

16. Lara-Carmona J, Amieva-Balmori M, Martinez-Conejo A, Jorge FJC, Garcia-Zermeno KR, Flores KH, et al. PREVALENCE OF CELIAC DISEASE (CD) IN SUBJECTS WITH DYSPEPTIC SYMPTOMS. A CASE-CONTROL STUDY. Gastroenterology. 2019;156(6 S1):S-918. doi: http://dx.doi.org/10.1016/S0016-5085%2819%2939262-5.

17. Tikkakoski S, Savilahti E, Kolho K-L. Undiagnosed coeliac disease and nutritional deficiencies in adults screened in primary health care. Scandinavian journal of gastroenterology. 2007;42(1):60-5.

18. Ludvigsson JF, Aro P, Walker MM, Vieth M, Agreus L, Talley NJ, et al. Celiac disease, eosinophilic esophagitis and gastroesophageal reflux disease, an adult population-based study. Scandinavian Journal of Gastroenterology. 2013;48(7):808-14. doi: http://dx.doi.org/10.3109/00365521.2013.792389.

19. Locke GR, 3rd, Murray JA, Zinsmeister AR, Melton LJ, 3rd, Talley NJ. Celiac disease serology in irritable bowel syndrome and dyspepsia: a population-based case-control study. Mayo Clinic proceedings. 2004;79(4):476-82.

20. Vivas S, Ruiz De Morales JM, Martinez J, Gonzalez MC, Martin S, Martin J, et al. Human recombinant anti-transglutaminase antibody testing is useful in the diagnosis of silent coeliac disease in a selected group of at-risk patients. European Journal of Gastroenterology and Hepatology. 2003;15(5):479-83. doi: http://dx.doi.org/10.1097/01.meg.0000059104.41030.1c.

21. Alexander TS, Oshilaja O, Define L. The incidence of celiac disease antibodies in plasma specimens with low hemoglobin levels. Contemporary Challenges in Autoimmunity. 2009;1173:186-9. doi: http://dx.doi.org/10.1111/j.1749-6632.2009.04755.x.

22. Javid G, Lone SN, Shoukat A, Khan BA, Yattoo GN, Shah A, et al. Prevalence of celiac disease in adult patients with iron-deficiency anemia of obscure origin in Kashmir (India). Indian Journal of Gastroenterology. 2015;34(4):314-9. doi: http://dx.doi.org/10.1007/s12664-015-0586-z.

23. Ucardag D, Guliter S, Ceneli O, Yakaryilmaz F, Atasoy P, Caglayan O. Celiac disease prevalence in patients with iron deficiency anemia of obscure origin. Turkish Journal of Gastroenterology. 2009;20(4):266-70. doi: http://dx.doi.org/10.4318/tjg.2009.0024.

24. Lasa JS, Olivera P, Soifer L, Moore R. Iron-deficiency anemia as a subclinical celiac disease presentation in an Argentinian population. Revista de Gastroenterologia de Mexico. 2017;82(3):270-3. doi: http://dx.doi.org/10.1016/j.rgmx.2016.12.008.

25. Cikrikcioglu MA, Halac G, Hursitoglu M, Erkal H, Cakirca M, Kinas BE, et al. Prevalence of gluten sensitive enteropathy antibodies in restless legs syndrome. Acta neurologica Belgica. 2011;111(4):282-6.

26. Morawiec-Szymonik E, Foltyn W, Marek B, Glogowska-Szelag J, Kos-Kudla B, Kajdaniuk D. Antibodies involved in the development of pernicious anemia and other autoimmune diseases. Polish archives of internal medicine. 2020;130(1):31-7. doi: https://dx.doi.org/10.20452/pamw.15094.

27. Ransford RAJ, Hayes M, Palmer M, Hall MJ. A controlled, prospective screening study of celiac disease presenting as iron deficiency anemia. Journal of Clinical Gastroenterology. 2002;35(3):228-33. doi: http://dx.doi.org/10.1097/00004836-200209000-00006.

28. Abu-Zeid YA, Jasem WS, Lebwohl B, Green PH, ElGhazali G. Seroprevalence of celiac disease among United Arab Emirates healthy adult nationals: A gender disparity. World Journal of Gastroenterology. 2014;20(42):15830-6. doi: http://dx.doi.org/10.3748/wjg.v20.i42.15830.

29. Sanders DS, Patel D, Stephenson TJ, Ward AM, McCloskey EV, Hadjivassiliou M, et al. A primary care cross-sectional study of undiagnosed adult coeliac disease. European Journal of Gastroenterology and Hepatology. 2003;15(4):407-13. doi: http://dx.doi.org/10.1097/00042737-200304000-00012.

30. Yap TWC, Chan WK, Leow AHR, Azmi AN, Loke MF, Vadivelu J, et al. Prevalence of serum celiac antibodies in a multiracial asian population - A first study in the Young Asian adult population of Malaysia. PLoS ONE. 2015;10(3):e0121908. doi: http://dx.doi.org/10.1371/journal.pone.0121908.

31. Greco L, Veneziano A, Di Donato L, Zampella C, Pecoraro M, Paladini D, et al. Undiagnosed coeliac disease does not appear to be associated with unfavourable outcome of pregnancy. Gut. 2004;53(1):149-51. doi: http://dx.doi.org/10.1136/gut.53.1.149.

32. Kalayci AG, Kanber Y, Birinci A, Yildiz L, Albayrak D. The prevalence of coeliac disease as detected by screening in children with iron deficiency anaemia. Acta Paediatrica, International Journal of Paediatrics. 2005;94(6):678-81. doi: http://dx.doi.org/10.1080/08035250510025879.

33. Shahriari M, Honar N, Yousefi A, Javaherizadeh H. Association of potential celiac disease and refractory iron deficiency anemia in children and adolescents. Arquivos de Gastroenterologia. 2018;55(1):78-81. doi: http://dx.doi.org/10.1590/s0004-2803.201800000-15.

34. Narang M, Natarajan R, Shah D, Puri AS, Manchanda V, Kotru M. Celiac Disease in Children with Moderate-to-Severe Iron-deficiency Anemia. Indian Pediatrics. 2018;55(1):31-4. doi: http://dx.doi.org/10.1007/s13312-018-1223-6.

35. Ertekin V, Selimoglu MA, Kardas F, Aktas E. Prevalence of celiac disease in Turkish children. Journal of Clinical Gastroenterology. 2005;39(8):689-91. doi: http://dx.doi.org/10.1097/01.mcg.0000174026.26838.56.

36. Bizzaro N, Villalta D, Tonutti E, Doria A, Tampoia M, Bassetti D, et al. IgA and IgG Tissue Transglutaminase Antibody Prevalence and Clinical Significance in Connective Tissue Diseases, Inflammatory Bowel Disease, and Primary Biliary Cirrhosis. Digestive Diseases and Sciences. 2003;48(12):2360-5. doi: http://dx.doi.org/10.1023/B:DDAS.0000007875.72256.e8.

37. Feighery L, Collins C, Feighery C, Mahmud N, Coughlan G, Willoughby R, et al. Anti-transglutaminase antibodies and the serological diagnosis of coeliac disease. British Journal of Biomedical Science. 2003;60(1):14-8. doi: http://dx.doi.org/10.1080/09674845.2003.11783671.

38. Nisihara RM, Skare TL, Silva MB, Utiyama S. Rheumatoid arthritis and anti-endomysial antibodies. Acta Reumatologica Portuguesa. 2007;32(2):163-7. PubMed PMID: WOS:000254255800009.

39. Picarelli A, Di Tola M, Sabbatella L, Vetrano S, Anania MC, Spadaro A, et al. Anti-tissue transglutaminase antibodies in arthritic patients: a disease-specific finding? Clinical chemistry. 2003;49(12):2091-4.

40. Riente L, Chimenti D, Pratesi F, Delle Sedie A, Tommasi S, Tommasi C, et al. Antibodies to tissue transglutaminase and Saccharomyces cerevisiae in Ankylosing spondylitis and psoriatic arthritis. Journal of Rheumatology. 2004;31(5):920-4. PubMed PMID: WOS:000221246300015.

41. Luft LM, Barr SG, Martin LO, Chan EKL, Fritzler MJ. Autoantibodies to Tissue Transglutaminase in Sjogren's Syndrome and Related Rheumatic Diseases. Journal of Rheumatology. 2003;30(12):2613-9.

42. Heikkila K, Heliovaara M, Impivaara O, Kroger H, Knekt P, Rissanen H, et al. Celiac disease autoimmunity and hip fracture risk: Findings from a prospective cohort study. Journal of Bone and Mineral Research. 2015;30(4):630-6. doi: http://dx.doi.org/10.1002/jbmr.2380.

43. Gheita TA, Fawzy SM, Nour El-Din AM, Gomaa HE. Asymptomatic celiac sprue in juvenile rheumatic diseases children. International journal of rheumatic diseases. 2012;15(2):220-6. doi: https://dx.doi.org/10.1111/j.1756-185X.2011.01681.x.

44. Sahin Y, Sahin S, Barut K, Cokugras FC, Erkan T, Adrovic A, et al. The frequency of the celiac disease in patients with juvenile idiopathic arthritis. Journal of Pediatric Gastroenterology and Nutrition. 2017;64(Supplement 1):222-3. doi: http://dx.doi.org/10.1097/01.mpg.0000516381.25680.b4.

45. Skrabl-Baumgartner A, Christine Hauer A, Erwa W, Jahnel J. HLA genotyping as first-line screening tool for coeliac disease in children with juvenile idiopathic arthritis. Archives of disease in childhood. 2017;102(7):607-11. doi: https://dx.doi.org/10.1136/archdischild-2016-311544.

46. Stagi S, Giani T, Simonini G, Falcini F. Thyroid function, autoimmune thyroiditis and coeliac disease in juvenile idiopathic arthritis. Rheumatology. 2005;44(4):517-20. doi: http://dx.doi.org/10.1093/rheumatology/keh531.

47. Robazzi TC, Adan LF, Pimentel K, Guimaraes I, Magalhaes J, Toralles MB, et al. Autoimmune endocrine disorders and coeliac disease in children and adolescents with juvenile idiopathic arthritis and rheumatic fever. Clinical and Experimental Rheumatology. 2013;31(2):310-7. PubMed PMID: WOS:000317086600023.

48. Taneja A, Prahalad S, Hersh AO, Ponder L, Chan LHK, Rouster-Stevens KA, et al. Prevalence of celiac antibodies and IgA deficiency in juvenile idiopathic arthritis. Arthritis and Rheumatology. 2017;69(Supplement 4):22-3. doi: http://dx.doi.org/10.1002/art.v69.S4.

49. Togrol RE, Nalbant S, Solmazgul E, Ozyurt M, Kaplan M, Kiralp MZ, et al. The significance of coeliac disease antibodies in patients with ankylosing spondylitis: A case-controlled study. Journal of International Medical Research. 2009;37(1):220-6. doi: http://dx.doi.org/10.1177/147323000903700127.

50. Nenna R, Tiberti C, Petrarca L, Lucantoni F, Mennini M, Luparia RPL, et al. The celiac iceberg: characterization of the disease in primary schoolchildren. Journal of pediatric gastroenterology and nutrition. 2013;56(4):416-21. doi: https://dx.doi.org/10.1097/MPG.0b013e31827b7f64.

51. Cakir M, Cezaroglu S, Cobanoglu U. Celiac disease in children with chronic constipation. Turkish Journal of Medical Sciences. 2016;46(3):651-6. doi: http://dx.doi.org/10.3906/sag-1502-130.

52. Fifi AC, Velasco-Benitez C, Saps M. Celiac Disease in Children with Functional Constipation: A School-Based Multicity Study. Journal of Pediatrics. 2020. doi: http://dx.doi.org/10.1016/j.jpeds.2020.07.052. PubMed PMID: 2007854302.

53. Kumar V, Jarzabek-Chorzelska M, Sulej J, Rajadhyaksha M, Jablonska S. Tissue transglutaminase and endomysial antibodies - Diagnostic markers of gluten-sensitive enteropathy in dermatitis herpetiformis. Clinical Immunology. 2001;98(3):378-82. doi: http://dx.doi.org/10.1006/clim.2000.4983.

54. Velikova T, Shahid M, Ivanova-Todorova E, Drenovska K, Tumangelova-Yuzeir K, Altankova I, et al. Celiac-Related Autoantibodies and IL-17A in Bulgarian Patients with Dermatitis Herpetiformis: A Cross-Sectional Study. Medicina (Kaunas, Lithuania). 2019;55(5). doi: https://dx.doi.org/10.3390/medicina55050136.

55. Smecuol E, Sugai E, Niveloni S, Vazquez H, Pedreira S, Mazure R, et al. Permeability, zonulin production, and enteropathy in dermatitis herpetiformis. Clinical Gastroenterology and Hepatology. 2005;3(4):335-41. doi: http://dx.doi.org/10.1016/S1542-3565%2804%2900778-5.

56. Imanzadeh F, Sayyari AA, Yaghoobi M, Akbari MR, Shafagh H, Farsar AR. Celiac disease in children with diarrhea is more frequent than previously suspected. Journal of Pediatric Gastroenterology and Nutrition. 2005;40(3):309-11. doi: http://dx.doi.org/10.1097/01.MPG.0000154012.10420.08.

57. Ranua J, Luoma K, Auvinen A, Maki M, Haapala A-M, Peltola J, et al. Celiac disease-related antibodies in an epilepsy cohort and matched reference population. Epilepsy & behavior : E&B. 2005;6(3):388-92.

58. Mavroudi A, Xinias I, Papastavrou T, Karatza E, Fotoulaki M, Panteliadis C, et al. Increased prevalence of silent celiac disease among Greek epileptic children. Pediatric neurology. 2007;36(3):165-9.

59. Antigoni M, Xinias I, Theodouli P, Karatza E, Maria F, Panteliadis C, et al. Increased Prevalence of Silent Celiac Disease Among Greek Epileptic Children. Pediatric Neurology. 2007;36(3):165-9. doi: http://dx.doi.org/10.1016/j.pediatrneurol.2006.11.011.

60. Dalgic B, Dursun I, Serdaroglu A, Dursun A. Latent and potential celiac disease in epileptic Turkish children. Journal of Child Neurology. 2006;21(1):6-7. doi: http://dx.doi.org/10.1177/08830738060210010301.

61. Dai AI, Akcali A, Varan C, Demiryurek AT. Prevalence of resistant occipital lobe epilepsy associated with celiac disease in children. Child's Nervous System. 2014;30(6):1091-8. doi: http://dx.doi.org/10.1007/s00381-014-2387-6.

62. Djuric Z, Nagorni A, Jocic-Jakubi B, Dimic M, Novak M, Milicevic R, et al. Celiac disease prevalence in epileptic children from Serbia. Turkish Journal of Pediatrics. 2012;54(3):247-50.

63. Giordano L, Valotti M, Bosetti A, Accorsi P, Caimi L, Imberti L. Celiac Disease-Related Antibodies in Italian Children With Epilepsy. Pediatric Neurology. 2009;41(1):34-6. doi: http://dx.doi.org/10.1016/j.pediatrneurol.2009.02.009.

64. Isikay S, Hizli S, Yilmaz K. Prevalence of celiac disease in Turkish children with idiopathic epilepsy. Iranian Journal of Pediatrics. 2014;24(3):280-4.

65. Isikay S, Kocamaz H. Prevalence of celiac disease in children with idiopathic epilepsy in Southeast Turkey. Pediatric Neurology. 2014;50(5):479-81. doi: http://dx.doi.org/10.1016/j.pediatrneurol.2014.01.021.

66. Lahat E, Broide E, Leshem M, Evans S, Scapa E. Prevalence of celiac antibodies in children with neurologic disorders. Pediatric Neurology. 2000;22(5):393-6. doi: http://dx.doi.org/10.1016/S0887-8994%2800%2900129-6.

67. Pratesi R, Gandolfi L, Martins RC, Tauil PL, Nobrega YK, Teixeira WA. Is the prevalence of celiac disease increased among epileptic patients? Arquivos de Neuro-Psiquiatria. 2003;61(2 B):330-4. doi: http://dx.doi.org/10.1590/S0004-282X2003000300002.

68. Soni S, Agarwal A, Singh A, Gupta V, Khadgawat R, Chaturvedi PK, et al. Prevalence of thyroid autoimmunity in first-degree relatives of patients with celiac disease. Indian Journal of Gastroenterology. 2019;38(5):450-5. doi: http://dx.doi.org/10.1007/s12664-019-00990-3.

69. Choung RS, Horwath IE, Marietta E, Bublitz JT, Olson JE, Murray JA. PREVALENCE OF CELIAC DISEASE AUTOIMMUNITY AND ASSOCIATED MORBID CONDITIONS IN FIRST-DEGREE RELATIVES OF PATIENTS WITH CELIAC DISEASE. Gastroenterology. 2019;156(6 Supplement 1):S-136. doi: http://dx.doi.org/10.1016/S0016-5085%2819%2937130-6.

70. Fasano A, Berti I, Gerarduzzi T, Not T, Colletti RB, Drago S, et al. Prevalence of Celiac disease in at-risk and not-at-risk groups in the United States: A large multicenter study. Archives of Internal Medicine. 2003;163(3):286-92. doi: http://dx.doi.org/10.1001/archinte.163.3.286.

71. Beser OF, Gulluelli E, Cokugras FC, Erkan T, Kutlu T, Yagci RV, et al. Prevalence and Clinical Features of Celiac Disease in Healthy School-Aged Children. Digestive Diseases and Sciences. 2019;64(1):173-81. doi: 10.1007/s10620-018-5320-0. PubMed PMID: WOS:000454932700025.

72. Cintado A, Sorell L, Galvan JA, Martinez L, Castaneda C, Fragoso T, et al. HLA DQA1*0501 and DQB 1*02 in Cuban celiac patients. Human Immunology. 2006;67(8):639-42. doi: 10.1016/j.humimm.2006.04.009. PubMed PMID: WOS:000240289100011.

73. Kotze LM, Utiyama SR, Nisihara RM, Zeni MP, de Sena MG, Amarante HM. Antiendomysium antibodies in Brazilian patients with celiac disease and their first-degree relatives. Arquivos de gastroenterologia. 2001;38(2):94-103.

74. Nass FR, Kotze LMD, Nisiharaa RM, de Messias-Reason LJ, Utiyama SRD. Serological and Clinical Follow-Up of Relatives of Celiac Disease Patients from Southern Brazil. Digestion. 2011;83(1-2):89-95. doi: 10.1159/000320451. PubMed PMID: WOS:000284742700014.

75. Utiyama SRDR, Nass FR, Kotze LMDS, Nisihara RM, Ambrosio AR, De Messias-Reason IT. Serological screening of relatives of celiac disease patients: Antiendomysium antibodies, anti-tissue transglutaminase or both? Arquivos de Gastroenterologia. 2007;44(2):156-61.

76. Hjelle AM, Apalset E, Mielnik P, Nilsen RM, Lundin KEA, Tell GS. Positive IgA against transglutaminase 2 in patients with distal radius and ankle fractures compared to community-based controls. Scandinavian Journal of Gastroenterology. 2018;53(10-11):1212-6. doi: http://dx.doi.org/10.1080/00365521.2018.1509122.

77. Hjelle AM, Mielnik P, Apalset E, Tell GS. Celiac disease and positive iga tissue transglutaminase in patients with distal radius or ankle fracture: Interim analysis. Annals of the Rheumatic Diseases. 2014;73(SUPPL. 2). doi: http://dx.doi.org/10.1136/annrheumdis-2014-eular.1238.

78. LeBoff MS, Cobb H, Gao LY, Hawkes W, Yu-Yahiro J, Kolatkar NS, et al. Celiac disease is not increased in women with hip fractures and low vitamin D levels. Journal of Nutrition, Health and Aging. 2013;17(6):562-5. doi: http://dx.doi.org/10.1007/s12603-013-0017-8.

79. Potter MDE, Walker MM, Hancock S, Holliday E, Brogan G, Jones M, et al. A serological diagnosis of coeliac disease is associated with osteoporosis in older Australian adults. Nutrients. 2018;10(7):849. doi: http://dx.doi.org/10.3390/nu10070849.

80. Agardh D, Bjorck S, Agardh CD, Lidfeldt J. Coeliac disease-specific tissue transglutaminase autoantibodies are associated with osteoporosis and related fractures in middle-aged women. Scandinavian Journal of Gastroenterology. 2009;44(5):571-8. doi: http://dx.doi.org/10.1080/00365520902718929.

81. West J, Logan RFA, Hill PG, Lloyd A, Lewis S, Hubbard R, et al. Seroprevalence, correlates, and characteristics of undetected coeliac disease in England. Gut. 2003;52(7):960-5. doi: http://dx.doi.org/10.1136/gut.52.7.960.

82. Choung RS, Larson SA, Khaleghi S, Rubio-Tapia A, Ovsyannikova IG, King KS, et al. Prevalence and Morbidity of Undiagnosed Celiac Disease From a Community-Based Study. Gastroenterology. 2017;152(4):830-9. doi: http://dx.doi.org/10.1053/j.gastro.2016.11.043.

83. Walker MM, Murray JA, Ronkainen J, Aro P, Storskrubb T, D'Amato M, et al. Detection of Celiac Disease and Lymphocytic Enteropathy by Parallel Serology and Histopathology in a Population-Based Study. Gastroenterology. 2010;139(1):112-9. doi: http://dx.doi.org/10.1053/j.gastro.2010.04.007.

84. Sandstrom O, Rosen A, Lagerqvist C, Carlsson A, Hernell O, Hogberg L, et al. Transglutaminase IgA antibodies in a celiac disease mass screening and the role of HLA-DQ genotyping and endomysial antibodies in sequential testing. Journal of Pediatric Gastroenterology and Nutrition. 2013;57(4):472-6. doi: http://dx.doi.org/10.1097/MPG.0b013e31829ef65d.

85. Beth SA, Jansen MAE, Elfrink MEC, Kiefte-de Jong JC, Wolvius EB, Jaddoe VWV, et al. Generation R birth cohort study shows that specific enamel defects were not associated with elevated serum transglutaminase type 2 antibodies. Acta Paediatrica. 2016;105(10):e485-e91. doi: 10.1111/apa.13533. PubMed PMID: WOS:000383619400009.

86. Maki M, Mustalahti K, Kokkonen J, Kulmala P, Haapalahti M, Karttunen T, et al. Prevalence of Celiac disease among children in Finland. The New England journal of medicine. 2003;348(25):2517-24.

87. Bjorck S, Brundin C, Lorinc E, Lynch KF, Agardh D. Screening detects a high proportion of celiac disease in young HLA-genotyped children. Journal of Pediatric Gastroenterology and Nutrition. 2010;50(1):49-53. doi: http://dx.doi.org/10.1097/MPG.0b013e3181b477a6.

88. Khudher SN, Mohammed KAS, Ali NH. Influence of HLA-DQ on clinical and serological biomarkers in patients with celiac disease. Annals of Tropical Medicine and Public Health. 2020;23 (16) (no pagination)(SP231617). doi: http://dx.doi.org/10.36295/ASRO.2020.231617. PubMed PMID: 2010049958.

89. Horoldt BS, Leeds JS, Sidhu R, Hopper AD, Robinson K, Toulson B, et al. Is there a relationship between coeliac disease and inflammatory bowel disease? A bidirectional prevalence study with controls. Gut. 2006;55:A98-A. PubMed PMID: WOS:000235922100376.

90. Leeds JS, Hoeroldt BS, Sidhu R, Hopper AD, Robinson K, Toulson B, et al. Is there an association between coeliac disease and inflammatory bowel diseases? A study of relative prevalence in comparison with population controls. Scandinavian Journal of Gastroenterology. 2007;42(10):1214-20. doi: 10.1080/00365520701365112. PubMed PMID: WOS:000249955900011.

91. Watanabe C, Komoto S, Hokari R, Kurihara C, Okada Y, Hozumi H, et al. Prevalence of serum celiac antibody in patients with IBD in Japan. Journal of Gastroenterology. 2014;49(5):825-34. doi: http://dx.doi.org/10.1007/s00535-013-0838-6.

92. El-Matary W, Fedorak RN, Senthilselvan A, Spady D. Celiac disease and inflammatory bowel disease in children: Is there a link? Gastroenterology. 2012;142(5 SUPPL. 1):S370-S1.

93. Kull K, Uibo O, Salupere R, Metskula K, Uibo R. High frequency of antigliadin antibodies and absence of antireticulin and antiendomysium antibodies in patients with ulcerative colitis. Journal of Gastroenterology. 1999;34(1):61-5. doi: http://dx.doi.org/10.1007/s005350050217.

94. Mehdi Z, Sakineh E, Mohammad F, Mansour R, Alireza A. Celiac disease: Serologic prevalence in patients with irritable bowel syndrome. Journal of Research in Medical Sciences. 2012;17(9):839-42.

95. Respondek W, Tomasiuk R, Jarosz M, Traczyk I, Mekus M. Is it reasonable to perform serological tests for celiac disease in patients with irritable bowel syndrome? Przeglad Gastroenterologiczny. 2013;8(3):184-90. doi: http://dx.doi.org/10.5114/pg.2013.36333.

96. Almazar AE, Talley NJ, Larson JJ, Atkinson EJ, Murray JA, Saito YA. Celiac disease is uncommon in irritable bowel syndrome in the USA. European Journal of Gastroenterology and Hepatology. 2018;30(2):149-54. doi: http://dx.doi.org/10.1097/MEG.0000000000001022.

97. Cash BD, Rubenstein JH, Young PE, Gentry A, Nojkov B, Lee D, et al. The prevalence of celiac disease among patients with nonconstipated irritable bowel syndrome is similar to controls. Gastroenterology. 2011;141(4):1187-93. doi: http://dx.doi.org/10.1053/j.gastro.2011.06.084.

98. Domzal-Magrowska D, Kowalski MK, Szczesniak P, Bulska M, Orszulak-Michalak D, Malecka-Panas E. The prevalence of celiac disease in patients with irritable bowel syndrome and its subtypes. Przeglad Gastroenterologiczny. 2016;11(4):276-81. doi: http://dx.doi.org/10.5114/pg.2016.57941.

99. Kou GJ, Guo J, Zuo XL, Li CQ, Liu C, Ji R, et al. Prevalence of celiac disease in adult Chinese patients with diarrhea-predominant irritable bowel syndrome: A prospective, controlled, cohort study. Journal of Digestive Diseases. 2018;19(3):136-43. doi: http://dx.doi.org/10.1111/1751-2980.12587.

100. Saito-Loftus Y, Brantner T, Zimmerman J, Talley N, Murray J. The prevalence of positive serologic tests for celiac sprue does not differ between irritable bowel syndrome (IBS) patients compared with controls. American Journal of Gastroenterology. 2008;103:S472-S. PubMed PMID: WOS:000259145201197.

101. Sanchez-Vargas LA, Thomas-Dupont P, Torres-Aguilera M, Azamar-Jacome AA, Ramirez-Ceervanes KL, Aedo-Garces MR, et al. Prevalence of celiac disease and related antibodies in patients diagnosed with irritable bowel syndrome according to the Rome III criteria. A case-control study. Neurogastroenterology and Motility. 2016;28(7):994-1000. doi: http://dx.doi.org/10.1111/nmo.12799.

102. Sanders DS, Carter MJ, Hurlstone DP, Pearce A, Ward AM, McAlindon ME, et al. Association of adult coeliac disease with irritable bowel syndrome: A case-control study in patients fulfilling ROME II criteria referred to secondary care. Lancet. 2001;358(9292):1504-8. doi: http://dx.doi.org/10.1016/S0140-6736%2801%2906581-3.

103. Vargas LAS, Garces M, Dupont PT, Meixueiro A, Jacome AAA, Roesch FB, et al. Prevalence of Antibodies Related to Celiac Disease (CD) in Patients With Irritable Bowel Syndrome (IBS) According to ROME III Criteria. A Case-Control Study. Gastroenterology. 2013;144(5):S252-S. PubMed PMID: WOS:000322997201287.

104. Wang H, Zhou G, Luo L, Crusius JBA, Yuan A, Kou J, et al. Serological screening for celiac disease in adult Chinese patients with diarrhea predominant irritable bowel syndrome. Medicine (United States). 2015;94(42):e1779. doi: http://dx.doi.org/10.1097/MD.0000000000001779.

105. Khayyat YM. Undiagnosed Celiac Disease In Patients Presenting With Irritable Bowel Syndrome Symptoms. Journal of Ayub Medical College, Abbottabad: JAMC. 2020;32(1):18-23. PubMed PMID: 32468748.

106. Olen O, Sandstrom O, Myleus A, Rosen A, Carlsson A, Hogberg L, et al. Functional gastrointestinal disorders and the association to celiac disease-a population based screening study in children. Gastroenterology. 2014;146(5 SUPPL. 1):S-173. doi: http://dx.doi.org/10.1016/S0016-5085%2814%2960611-9.

107. Chatzicostas C, Roussomoustakaki M, Drygiannakis D, Niniraki M, Tzardi M, Koulentaki M, et al. Primary biliary cirrhosis and autoimmune cholangitis are not associated with coeliac disease in Crete. BMC Gastroenterology. 2002;2:5. doi: http://dx.doi.org/10.1186/1471-230X-2-5.

108. Durante-Mangoni E, Iardino P, Resse M, Cesaro G, Sica A, Farzati B, et al. Silent celiac disease in chronic hepatitis C - Impact of interferon treatment on the disease onset and clinical outcome. Journal of Clinical Gastroenterology. 2004;38(10):901-5. doi: 10.1097/00004836-200411000-00014. PubMed PMID: WOS:000224718100014.

109. Hernandez L, Johnson TC, Naiyer AJ, Kryszak D, Ciaccio EJ, Min A, et al. Chronic hepatitis C virus and celiac disease, is there an association? Digestive diseases and sciences. 2008;53(1):256-61.

110. Sjoberg K, Lindgren S, Eriksson S. Frequent occurrence of non-specific gliadin antibodies in chronic liver disease: Endomysial but not gliadin antibodies predict coeliac disease in patients with chronic liver diseases. Scandinavian Journal of Gastroenterology. 1997;32(11):1162-7. doi: http://dx.doi.org/10.3109/00365529709002997.

111. Yuan J, Gao J, Yao Y, Chen H. Serologic testing for celiac disease in young people with elevated transaminases. Turkish Journal of Medical Sciences. 2015;45(3):668-73. doi: http://dx.doi.org/10.3906/sag-1403-127.

112. El-Shabrawi M, El-Karaksy H, Mohsen N, Isa M, Al-Biltagi M, El-Ansari M. Celiac disease in children and adolescents with autoimmune hepatitis: A single-centre experience. Journal of Tropical Pediatrics. 2011;57(2):104-8. doi: http://dx.doi.org/10.1093/tropej/fmq057.

113. Oana B, Laura O, Ioan S, Tamara M, Otilia M. Celiac disease prevalence among children with autoimmune hepatitis and autoimmune thyroid disorders. A 10-years single centre exp erience. Journal of Gastrointestinal and Liver Diseases. 2018;27 (Supplement 2):79. PubMed PMID: 632512251.

114. Germenis AE, Yiannaki EE, Zachou K, Roka V, Barbanis S, Liaskos C, et al. Prevalence and clinical significance of immunoglobulin A antibodies against tissue transglutaminase in patients with diverse chronic liver diseases. Clinical and diagnostic laboratory immunology. 2005;12(8):941-8.

115. Villalta D, Girolami D, Bidoli E, Bizzaro N, Tampoia M, Liguori M, et al. High prevalence of celiac disease in autoimmune hepatitis detected by anti-tissue tranglutaminase autoantibodies. Journal of Clinical Laboratory Analysis. 2005;19(1):6-10. doi: http://dx.doi.org/10.1002/jcla.20047.

116. Gabrielli M, Fiore G, Addolorato G, Padalino C, Candelli M, De Leo ME, et al. Association between migraine and celiac disease: Results from a preliminary case-control and therapeutic study. (vol 98, pg 625, 2003). American Journal of Gastroenterology. 2003;98(7):1674-. PubMed PMID: WOS:000184216400051.

117. Balci O, Yilmaz D, Sezer T, Hizli S. Is Celiac Disease an Etiological Factor in Children With Migraine? Journal of child neurology. 2016;31(7):929-31. doi: https://dx.doi.org/10.1177/0883073816630088.

118. Alehan F, Ozcay F, Erol I, Canan O, Cemil T. Increased risk for coeliac disease in paediatric patients with migraine. Cephalalgia. 2008;28(9):945-9. doi: http://dx.doi.org/10.1111/j.1468-2982.2008.01630.x.

119. Inaloo S, Dehghani SM, Farzadi F, Haghighat M, Imanieh MH. A comparative study of celiac disease in children with migraine headache and a normal control group. Turkish Journal of Gastroenterology. 2011;22(1):32-5. doi: http://dx.doi.org/10.4318/tjg.2011.0153.

120. Abolfazli R, Mirbagheri A, Rabbani Anari M, Samadzadeh S. The association of celiac disease with multiple sclerosis. Journal of the Neurological Sciences. 2009;285(SUPPL. 1):S214.

121. Nicoletti A, Patti F, Lo Fermo S, Sciacca A, Laisa P, Liberto A, et al. Frequency of celiac disease is not increased among multiple sclerosis patients. Multiple sclerosis (Houndmills, Basingstoke, England). 2008;14(5):698-700. doi: https://dx.doi.org/10.1177/1352458507087268.

122. Rodrigo L, Hernandez-Lahoz C, Fuentes D, Alvarez N, Lopez-Vazquez A, Gonzalez S. Prevalence of celiac disease in multiple sclerosis. BMC Neurology. 2011;11:31. doi: http://dx.doi.org/10.1186/1471-2377-11-31.

123. Roth EB, Theander E, Londos E, Sandberg-Wollheim M, Larsson A, Sjoberg K, et al. Pathogenesis of autoimmune diseases: Antibodies against transglutaminase, peptidylarginine deiminase and protein-bound citrulline in primary Sjogren's syndrome, multiple sclerosis and Alzheimer's disease. Scandinavian Journal of Immunology. 2008;67(6):626-31. doi: 10.1111/j.1365-3083.2008.02115.x. PubMed PMID: WOS:000255725000012.

124. Khoshbaten M, Farhoudi M, Nikanfar M, Ayromlou H, Shaafi S, Sadreddini SA, et al. Celiac disease and multiple sclerosis in the northwest of Iran. Bratislavske lekarske listy. 2012;113(8):495-7.

125. Gusso L, Simoes MC, Skare TL, Nisihara R, Burkiewicz CC, Utiyama S. Celiac disease screening in Brazilian patients with osteoporosis. Arquivos brasileiros de endocrinologia e metabologia. 2014;58(3):270-3.

126. Shahbazkhani B, Aletaha N, Khonche A, Farahvash B, Malekzadeh R. Is it necessary to screen for celiac disease in adult idiopathic osteoporosis? Gastroenterology and Hepatology from Bed to Bench. 2015;8(2):140-5.

127. Stenson WF, Newberry R, Lorenz R, Baldus C, Civitelli R. Increased prevalence of celiac disease and need for routine screening among patients with osteoporosis. Archives of internal medicine. 2005;165(4):393-9.

128. Vancikova Z, Chlumecky V, Sokol D, Horakova D, Hamsikova E, Fucikova T, et al. The serologic screening for celiac disease in the general population (blood donors) and in some high-risk groups of adults (patients with autoimmune diseases, osteoporosis and infertility) in the Czech republic. Folia microbiologica. 2002;47(6):753-8.

129. Shen Y, Wang M, Martinez D. Celiac disease and low femoral bone density in a multiethnic us national survey. Osteoporosis International. 2014;25(5 SUPPL. 1):578. doi: http://dx.doi.org/10.1007/s00198-014-2891-2.

130. De Bastiani R, Gabrielli M, Lora L, Napoli L, Tosetti C, Pirrotta E, et al. Association between coeliac disease and psoriasis: Italian primary care multicentre study. Dermatology. 2015;230(2):156-60. doi: http://dx.doi.org/10.1159/000369615.

131. Akbulut S, Gur G, Topal F, Senel E, Topal FE, Alli N, et al. Coeliac disease-associated antibodies in psoriasis. Annals of dermatology. 2013;25(3):298-303. doi: https://dx.doi.org/10.5021/ad.2013.25.3.298.

132. Dhattarwal N, Mahajan VK, Mehta KS, Chauhan PS, Yadav RS, Sharma SB, et al. The association of anti-gliadin and anti-transglutaminase antibodies and chronic plaque psoriasis in Indian patients: Preliminary results of a descriptive cross-sectional study. Australasian Journal of Dermatology. 2020;61(4):e378-e82. doi: http://dx.doi.org/10.1111/ajd.13308. PubMed PMID: 2004900740.

133. Montesu MA, Dessi-Fulgheri C, Pattaro C, Ventura V, Satta R, Cottoni F. Association Between Psoriasis and Coeliac Disease? A Case-control Study. Acta Dermato-Venereologica. 2011;91(1):92-3. doi: 10.2340/00015555-0960. PubMed PMID: WOS:000286553800027.

134. Nagui N, El Nabarawy E, Mahgoub D, Mashaly HM, Saad NE, El-Deeb DF. Estimation of (IgA) anti-gliadin, anti-endomysium and tissue transglutaminase in the serum of patients with psoriasis. Clinical and experimental dermatology. 2011;36(3):302-4. doi: https://dx.doi.org/10.1111/j.1365-2230.2010.03980.x.

135. Singh S, Sonkar GK, Usha, Singh S. Celiac disease-associated antibodies in patients with psoriasis and correlation with HLA Cw6. Journal of clinical laboratory analysis. 2010;24(4):269-72. doi: https://dx.doi.org/10.1002/jcla.20398.

136. Marai I, Shoenfeld Y, Bizzaro N, Villalta D, Doria A, Tonutti E, et al. IgA and IgG tissue transglutaminase antibodies in systemic lupus erythematosus. Lupus. 2004;13(4):241-4. doi: http://dx.doi.org/10.1191/0961203304lu1004oa.

137. Picceli VF, Skare TL, Nisihara R, Kotze L, Messias-Reason I, Utiyama SRR. Spectrum of autoantibodies for gastrointestinal autoimmune diseases in systemic lupus erythematosus patients. Lupus. 2013;22(11):1150-5. doi: http://dx.doi.org/10.1177/0961203313503911.

138. Sahin Y, Sahin S, Adrovic A, Erkan T, Kutlu T, Barut K, et al. The frequency of celiac disease in Turkish children with systemic lupus erythematosus. Journal of Pediatric Gastroenterology and Nutrition. 2017;64(Supplement 1):230. doi: http://dx.doi.org/10.1097/01.mpg.0000516381.25680.b4.

139. Cedikova M, Ulcova-Gallova Z, Bibkova K, Micanova Z. [The incidence of latent asymptomatic celiac disease in women with decreased fertility]. Vyskyt latentni asymptomaticke celiakie u zen se snizenou plodnosti. 2013;78(3):247-51.

140. Remes-Troche JM, Vargas LAS, Meixueiro A, Patino ED, Gonzalez-Sicilia E, Abreu JA, et al. Celiac Disease Screening in Patients Previously Diagnosed With Infertility. A Prospective Study in Mexican Population. Gastroenterology. 2014;146(5):S468-S. PubMed PMID: WOS:000371236402229.

141. Herraiz-Nicuesa L, Tejera-Alhambra M, Garcia-Segovia A, Ramos-Medina R, Alonso B, Gil-Pulido J, et al. Increasing prevalence of undiagnosed celiac disease in spanish women with recurrent reproductive failure. Human Reproduction. 2013;28(SUPPL. 1). doi: http://dx.doi.org/10.1093/humrep/det209.

142. Kumar A, Meena M, Begum N, Kumar N, Gupta RK, Aggarwal S, et al. Latent celiac disease in reproductive performance of women. Fertility and Sterility. 2011;95(3):922-7. doi: http://dx.doi.org/10.1016/j.fertnstert.2010.11.005.

143. Kutteh MA, Abiad M, Norman GL, Kutteh WH. Comparison of celiac disease markers in women with early recurrent pregnancy loss and normal controls. American Journal of Reproductive Immunology. 2019;82(1):e13127. doi: http://dx.doi.org/10.1111/aji.13127.

144. Sarikaya E, Tokmak A, Aksoy RT, Pekcan MK, Alisik M, Alkan A. The Association Between Serological Markers of Celiac Disease and Idiopathic Recurrent Pregnancy Loss. Fetal and Pediatric Pathology. 2017;36(5):373-9. doi: http://dx.doi.org/10.1080/15513815.2017.1346018.

145. Shamaly H, Mahameed A, Sharony A, Shamir R. Infertility and celiac disease: Do we need more than one serological marker? Acta Obstetricia et Gynecologica Scandinavica. 2004;83(12):1184-8. doi: http://dx.doi.org/10.1111/j.0001-6349.2004.00592.x.

146. Sharshiner R, Romero S, Silver R, Branch DW. Celiac disease serum markers and recurrent pregnancy loss. American Journal of Obstetrics and Gynecology. 2013;208(1 SUPPL.1):S75-S6. doi: http://dx.doi.org/10.1016/j.ajog.2012.10.315.

147. Tiboni GM, de Vita MG, Faricelli R, Giampietro F, Liberati M. Serological testing for celiac disease in women undergoing assisted reproduction techniques. Human Reproduction. 2006;21(2):376-9. doi: http://dx.doi.org/10.1093/humrep/dei314.

148. Zahmatkeshan M. Prevalence of coeliac disease in infertile women. Journal of Pediatric Gastroenterology and Nutrition. 2019;68(Supplement 1):199. doi: http://dx.doi.org/10.1097/MPG.0000000000002403.

149. Celdir MG, Choung RS, Rostamkolaei SK, Jansson-Knodell CL, King KS, Larson JJ, et al. Reproductive Characteristics and Pregnancy Outcomes in Hidden Celiac Disease Autoimmunity. American Journal of Gastroenterology. 2021;116(3):593-9. doi: https://dx.doi.org/10.14309/ajg.0000000000001148. PubMed PMID: 33560653.

150. Martinelli P, Troncone R, Paparo F, Torre P, Trapanese E, Fasano C, et al. Coeliac disease and unfavourable outcome of pregnancy. Gut. 2000;46(3):332-5.

151. Mohammed MA, Elrabbat AM, Omar NM, Shebl AM, Mansour AH, Elmasry E, et al. Celiac disease prevalence and its HLA-genotypic profile in Egyptian patients with type 1 diabetes mellitus. Trends in Medical Research. 2014;9(2):81-97. doi: http://dx.doi.org/10.3923/tmr.2014.81.97.

152. Picarelli A, Sabbatella L, Di Tola M, Vetrano S, Casale C, Anania MC, et al. Anti-endomysial antibody of IgG1 isotype detection strongly increases the prevalence of coeliac disease in patients affected by type I diabetes mellitus. Clinical and Experimental Immunology. 2005;142(1):111-5. doi: http://dx.doi.org/10.1111/j.1365-2249.2005.02866.x.

153. Dagdelen S, Hascelik G, Bayraktar M. Simultaneous triple organ specific autoantibody profiling in adult patients with type 1 diabetes mellitus and their first-degree relatives. International Journal of Clinical Practice. 2009;63(3):449-56. doi: 10.1111/j.1742-1241.2007.01619.x. PubMed PMID: WOS:000262953500016.

154. Guvenc S, Kaymakoglu S, Gurel N, Karsidag K, Demir K, Dincer D, et al. The prevalence of manifest and latent celiac disease in type 1 diabetes mellitus. Turkish Journal of Gastroenterology. 2002;13(2):103-7.

155. Hanukoglu A, Mizrachi A, Dalal I, Admoni O, Rakover Y, Bistritzer Z, et al. Extrapancreatic autoimmune manifestations in type 1 diabetes patients and their first-degree relatives: A multicenter study. Diabetes Care. 2003;26(4):1235-40. doi: http://dx.doi.org/10.2337/diacare.26.4.1235.

156. Kurien M, Leeds JS, Hopper AD, Wild G, Egner W, Tesfaye S, et al. Serological testing for coeliac disease in Type 1 diabetes mellitus: Is immunoglobulin A level measurement necessary? Diabetic Medicine. 2013;30(7):840-5. doi: http://dx.doi.org/10.1111/dme.12163.

157. Shivaprasad C, Kolly A, Pulikkal A, Kumar KMP. High prevalence of organ specific autoantibodies in Indian type 1 diabetic patients. Journal of Pediatric Endocrinology & Metabolism. 2017;30(7):707-12. doi: 10.1515/jpem-2017-0011. PubMed PMID: WOS:000408795100001.

158. Zhao Z, Zou J, Zhao L, Cheng Y, Cai H, Li M, et al. Celiac disease autoimmunity in patients with autoimmune diabetes and thyroid disease among Chinese population. PLoS ONE. 2016;11(7):e0157510. doi: http://dx.doi.org/10.1371/journal.pone.0157510.

159. Abu-Zekry M, Kryszak D, Diab M, Catassi C, Fasano A. Prevalence of celiac disease in egyptian children disputes the east west agriculture-dependent spread of the disease. Journal of Pediatric Gastroenterology and Nutrition. 2008;47(2):136-40. doi: http://dx.doi.org/10.1097/MPG.0b013e31815ce5d1.

160. Adlercreutz EH, Svensson J, Hansen D, Buschard K, Lernmark A, Mortensen HB, et al. Prevalence of celiac disease autoimmunity in children with type 1 diabetes: Regional variations across the Oresund strait between Denmark and southernmost Sweden. Pediatric Diabetes. 2015;16(7):504-9. doi: http://dx.doi.org/10.1111/pedi.12200.

161. Aktay AN, Lee PC, Kumar V, Parton E, Wyatt DT, Werlin SL. The prevalence and clinical characteristics of celiac disease in juvenile diabetes in Wisconsin. Journal of Pediatric Gastroenterology and Nutrition. 2001;33(4):462-5. doi: http://dx.doi.org/10.1097/00005176-200110000-00008.

162. Baptista ML, Koda YKL, Mitsunori R, Nisihara, Ioshii SO. Prevalence of celiac disease in Brazilian children and adolescents with type 1 diabetes mellitus. Journal of Pediatric Gastroenterology and Nutrition. 2005;41(5):621-4. doi: http://dx.doi.org/10.1097/01.mpg.0000181400.57884.c3.

163. Djuric Z, Stamenkovic H, Stankovic T, Milicevic R, Brankovic L, Ciric V, et al. Celiac disease prevalence in children and adolescents with type 1 diabetes from Serbia. Pediatrics International. 2010;52(4):579-83. doi: http://dx.doi.org/10.1111/j.1442-200X.2010.03085.x.

164. Frohnert BI, Simmons K, Liu E, Taki I, Klingensmith GJ, McFann K, et al. Poor glycemic control and celiac disease autoimmunity are independently associated with reduced bone mineral density in children with type 1 diabetes. Diabetes. 2015;64(SUPPL. 1):A72. doi: http://dx.doi.org/10.2337/db151385.

165. Gurau G, Dobre M, Nechita A. Anti-tissue transglutaminase antibodies in patients with anti-glutamate dehydrogenase positive type 1 diabetes mellitus. Revista Romana De Medicina De Laborator. 2012;20(3):39-46. PubMed PMID: WOS:000309439200005.

166. Hansson T, Dahlbom I, Tuvemo T, Frisk G. Silent coeliac disease is over-represented in children with type 1 diabetes and their siblings. Acta Paediatrica, International Journal of Paediatrics. 2015;104(2):185-91. doi: http://dx.doi.org/10.1111/apa.12823.

167. Krause I, Anaya JM, Fraser A, Barzilai O, Ram M, Abad V, et al. Anti-infectious antibodies and autoimmune-associated autoantibodies in patients with type i diabetes mellitus and their close family members. Contemporary Challenges in Autoimmunity. 2009;1173:633-9. doi: http://dx.doi.org/10.1111/j.1749-6632.2009.04619.x.

168. Soyucen E, Yilmaz S, Celtik C, Vatansever U, Oner N, Karasalihoglu S. Seroprevalence of autoimmune thyroiditis and celiac disease in children with insulin-dependent diabetes mellitus in the Thrace region of Turkey. Turkish Journal of Gastroenterology. 2010;21(3):231-5. doi: http://dx.doi.org/10.4318/tjg.2010.0093.

169. Velasco C, Ortiz C, Ruiz A. HLA typing in healthy and diabetic children with celiac disease in Cali, Colombia. Journal of Pediatric Gastroenterology and Nutrition. 2016;63(Supplement 2):S293. doi: http://dx.doi.org/10.1097/01.mpg.0000503536.79797.66.

170. Not T, Tommasini A, Tonini G, Buratti E, Pocecco M, Tortul C, et al. Undiagnosed coeliac disease and risk of autoimmune disorders in subjects with Type I diabetes mellitus. Diabetologia. 2001;44(2):151-5. doi: 10.1007/s001250051593. PubMed PMID: WOS:000167005200003.

171. Premawardhana LDKE, Wijeyaratne CN, Chen S, Wijesuriya M, Illangasekera U, Brooking H, et al. Islet cell, thyroid, adrenal and celiac disease related autoantibodies in patients with Type 1 diabetes from Sri Lanka. Journal of endocrinological investigation. 2006;29(11):968-74.

172. Jaeger C, Hatziagelaki E, Petzoldt R, Bretzel RG. Comparative analysis of organ-specific autoantibodies and celiac disease-associated antibodies in type 1 diabetic patients, their first-degree relatives, and healthy control subjects. Diabetes Care. 2001;24(1):27-32. doi: http://dx.doi.org/10.2337/diacare.24.1.27.

173. Kanungo A, Shtauvere-Brameus A, Samal KC, Sanjeevi CB. Autoantibodies to tissue transglutaminase in patients from eastern India with malnutrition-modulated diabetes mellitus, insulin-dependent diabetes mellitus, and non-insulin-dependent diabetes mellitus. In: Sanjeevi CB, editor. Immunology of Diabetes: Autoimmune Mechanisms and the Prevention and Cure of Type 1 Diabetes. Annals of the New York Academy of Sciences. 9582002. p. 232-4.

174. Sari S, Yesilkaya E, Egritas O, Bideci A, Cinaz P, Dalgic B. Prevalence of celiac disease in Turkish children with type 1 diabetes mellitus and their non-diabetic first-degree relatives. Turkish Journal of Gastroenterology. 2010;21(1):34-8. doi: http://dx.doi.org/10.4318/tjg.2010.0045.

175. Lampasona V, Bonfanti R, Bazzigaluppi E, Venerando A, Chiumello G, Bosi E, et al. Antibodies to tissue transglutaminase C in type I diabetes. Diabetologia. 1999;42(10):1195-8.

176. Sharifi N, Khoshbaten M, Aliasgarzade A, Bahrami A. Celiac disease in patients with type-1 diabetes mellitus screened by tissue transglutaminase antibodies in northwest of Iran. International journal of diabetes in developing countries. 2008;28(3):95-9. doi: https://dx.doi.org/10.4103/0973-3930.44081.

177. Sheikholeslami H, Boostani K, Hashemipoor S, Hadjmanoochehri F, Ziaii A. Comparing frequency of celiac disease in patients with diabetes mellitus type I and non-diabetic, healthy persons. Iranian Journal of Diabetes and Lipid Disorders. 2005;4(3).

178. Kizilgul M, Ozcelik O, Beysel S, Akinci H, Kan S, Ucan B, et al. Screening for celiac disease in poorly controlled type 2 diabetes mellitus: Worth it or not? BMC Endocrine Disorders. 2017;17(1):62. doi: http://dx.doi.org/10.1186/s12902-017-0212-4.

179. Szepietowska B, Wawrusiewicz-Kurylonek N, Kretowski A, Gorska M, Szelachowska M. Endocrine autoimmunity in patients with Latent Autoimmune Diabetes in Adults (LADA) - association with HLA genotype. Endokrynologia Polska. 2016;67(2):197-201. doi: 10.5603/EP.a2016.0017. PubMed PMID: WOS:000374546600007.

180. Ravaglia G, Forti P, Maioli F, Volta U, Arnone G, Pantieri G, et al. Increased prevalence of coeliac disease in autoimmune thyroiditis is restricted to aged patients. Experimental Gerontology. 2003;38(5):589-95. doi: http://dx.doi.org/10.1016/S0531-5565%2803%2900037-8.

181. Riseh SH, Farhang MA, Mobasseri M, Jafarabadi MA. THE RELATIONSHIP BETWEEN THYROID HORMONES, ANTITHYROID ANTIBODIES, ANTI-TISSUE TRANSGLUTAMINASE AND ANTI-GLIADIN ANTIBODIES IN PATIENTS WITH HASHIMOTO'S THYROIDITIS. Acta Endocrinologica-Bucharest. 2017;13(2):174-9. doi: 10.4183/aeb.2017.174. PubMed PMID: WOS:000406825700008.

182. Miskiewicz P, Gos-Zajac A, Kurylowicz A, Plazinska TM, Franaszczyk M, Bartoszewicz Z, et al. HLA DQ2 haplotype, early onset of graves disease, and positive family history of autoimmune disorders are risk factors for developing celiac disease in patients with graves disease. Endocrine Practice. 2015;21(9):993-1000. doi: http://dx.doi.org/10.4158/EP15700.OR.

183. Berti I, Trevisiol C, Tommasini A, Citta A, Neri E, Geatti O, et al. Usefulness of screening program for celiac disease in autoimmune thyroiditis. Digestive Diseases and Sciences. 2000;45(2):403-6. doi: http://dx.doi.org/10.1023/A:1005441400107.

184. Ch'ng CL, Biswas M, Benton A, Jones MK, Kingham JGC. Prospective screening for coeliac disease in patients with Graves' hyperthyroidism using anti-gliadin and tissue transglutaminase antibodies. Clinical Endocrinology. 2005;62(3):303-6. doi: http://dx.doi.org/10.1111/j.1365-2265.2005.02214.x.

185. Guliter S, Yakaryilmaz F, Ozkurt Z, Ersoy R, Ucardag D, Caglayan O, et al. Prevalence of coeliac disease in patients with autoimmune thyroiditis in a Turkish population. World journal of gastroenterology. 2007;13(10):1599-601.

186. Volta U, Ravaglia G, Granito A, Forti P, Maioli F, Petrolini N, et al. Coeliac disease in patients with autoimmune thyroiditis. Digestion. 2001;64(1):61-5. doi: http://dx.doi.org/10.1159/000048840.

187. Metzger MH, Heier M, Maki M, Bravi E, Schneider A, Lowel H, et al. Mortality excess in individuals with elevated IgA anti-transglutaminase antibodies: The KORA/MONICA Augsburg cohort study 1989-1998. European Journal of Epidemiology. 2006;21(5):359-65. doi: http://dx.doi.org/10.1007/s10654-006-9002-4.

188. Marwaha RK, Garg MK, Tandon N, Kanwar R, Narang A, Sastry A, et al. Glutamic acid decarboxylase (anti-GAD) & tissue transglutaminase (anti-TTG) antibodies in patients with thyroid autoimmunity. Indian Journal of Medical Research. 2013;137(1):82-6.

189. Sahin Y, Evliyaoglu O, Erkan T, Cokugras FC, Ercan O, Kutlu T. The frequency of celiac disease in children with autoimmune thyroiditis. Journal of Pediatric Gastroenterology and Nutrition. 2017;64(Supplement 1):227. doi: http://dx.doi.org/10.1097/01.mpg.0000516381.25680.b4.

190. Sari S, Yesilkaya E, Egritas O, Bideci A, Dalgic B. Prevalence of celiac disease in Turkish children with autoimmune thyroiditis. Digestive Diseases and Sciences. 2009;54(4):830-2. doi: http://dx.doi.org/10.1007/s10620-008-0437-1.

191. van der Pals M, Ivarsson A, Norström F, Högberg L, Svensson J, Carlsson A. Prevalence of thyroid autoimmunity in children with celiac disease compared to healthy 12-year olds. Autoimmune diseases. 2014;2014:417356. Epub 2014/03/05. doi: 10.1155/2014/417356. PubMed PMID: 24592326; PubMed Central PMCID: PMCPMC3921936.

192. Risan FA. Association between autoimmune thyroiditis with gluten sensitive enteropathy patients in Baghdad. Journal of Pharmaceutical Sciences and Research. 2018;10(12):3307-9.

193. Melo FMd, Cavalcanti MSM, Santos SBd, Lopes AKBF, Oliveira FAAd. [Association between serum markers for celiac and thyroid autoimmune diseases]. Associacao entre marcadores sorologicos de doenca celiaca e das doencas autoimunes da tireoide. 2005;49(4):542-7.
