# Supplementary material for: The accuracy of diagnostic indicators for coeliac disease: A systematic review and meta-analysis
Source: PLoS One. 2021 Oct 25;16(10):e0258501. doi: 10.1371/journal.pone.0258501 (PMC8545431; doi:10.1371/journal.pone.0258501)
Supplement: S3 Table — (DOCX) [file pone.0258501.s007.docx]

## Table S3: Summary estimates of sensitivity, specificity, and prediction values

Meta-analysis results, number of studies, total numbers of true positives (TP), false positives (FP), false negatives (FN), and true negatives (TN) are shown per diagnostic indicator. Tau represents the between-study standard deviation in sensitivity and specificity on the logit scale. *PPVs are calculated using the summary estimates of sensitivity and specificity for the general population assuming a 1% CD prevalence.

| **Diagnostic indicator** | **Studies** | **Sample size** | **TP** | **FP** | **FN** | **TN** | **Sensitivity** | **Tau sensitivity** | **Specificity** | **Tau specificity** | **PPV*** |
| --- | --- | --- | --- | --- | --- | --- | --- | --- | --- | --- | --- |
|  |  |  |  |  |  |  | **(95% CI)** |  | **(95% CI)** |  | **(95% CI)** |
| **Symptoms** |  |  |  |  |  |  |  |  |  |  |  |
| Abdominal pain | 12 | 48451 | 255 | 14345 | 759 | 33092 | 39.11 (24.03-56.61) | 1.15 | 73.51 (56.95-85.34) | 1.31 | 1.47 (0.95-2.33) |
| Diarrhoea | 13 | 55500 | 161 | 6384 | 965 | 47990 | 11.88 (4.52-27.73) | 1.81 | 91.53 (82.38-96.15) | 1.53 | 1.4 (0.76-2.46) |
| Constipation | 12 | 54286 | 101 | 7217 | 842 | 46126 | 15.87 (10.35-23.57) | 0.72 | 86.8 (80.47-91.3) | 0.82 | 1.2 (0.83-1.74) |
| Acid reflux symptoms | 10 | 12192 | 54 | 3169 | 480 | 8489 | 30.86 (12.93-57.29) | 1.68 | 72.07 (54.87-84.57) | 1.21 | 1.1 (0.77-1.4) |
| Bloating or abdominal distension | 6 | 32694 | 64 | 5809 | 560 | 26261 | 18.3 (7.09-39.69) | 1.28 | 83.26 (62.41-93.71) | 1.36 | 1.09 (0.79-1.52) |
| Weight loss | 5 | 31739 | 52 | 4722 | 769 | 26196 | 6.3 (2.23-16.54) | 1.17 | 94.09 (86.95-97.44) | 0.98 | 1.06 (0.71-1.55) |
| Vomiting and nausea | 7 | 44937 | 48 | 4898 | 387 | 39604 | 8.5 (2.49-25.23) | 1.52 | 91.88 (77.96-97.31) | 1.45 | 1.05 (0.67-1.6) |
| **Risk conditions** |  |  |  |  |  |  |  |  |  |  |  |
| Dermatitis herpetiformis | 5 | 1429 | 64 | 36 | 515 | 814 | 92.64 (4.34-99.97) | 5.2 | 97.69 (68.32-99.88) | 3.05 | 28.83 (2.88-72.44) |
| Migraine | 5 | 2478 | 10 | 375 | 32 | 2061 | 74.3 (21.45-96.84) | 2.09 | 73.6 (54.96-86.44) | 0.93 | 2.76 (1.24-3.89) |
| Anaemia | 17 | 13477 | 153 | 2346 | 562 | 10416 | 63.45 (36.89-83.75) | 2.08 | 75.25 (61.06-85.5) | 1.38 | 2.52 (1.94-3.16) |
| Type 1 Diabetes | 31 | 26635 | 699 | 6047 | 650 | 19239 | 79.27 (61.95-89.98) | 2.13 | 67.64 (53.44-79.2) | 1.66 | 2.41 (1.85-3.21) |
| Osteoporosis | 9 | 20218 | 44 | 1777 | 918 | 17479 | 20.03 (3.77-61.56) | 2.74 | 91.15 (71.89-97.65) | 2.12 | 2.24 (1.38-3) |
| Chronic liver disease | 11 | 8682 | 38 | 2144 | 410 | 6090 | 52.35 (17.3-85.23) | 2.84 | 76.09 (56.22-88.75) | 1.72 | 2.16 (1.29-2.86) |
| Psoriasis | 6 | 1127 | 34 | 498 | 10 | 585 | 90.61 (42.85-99.2) | 1.2 | 54.07 (51.02-57.08) | 0.02 | 1.95 (0.93-2.2) |
| Thyroid disease | 23 | 27031 | 212 | 4752 | 871 | 21196 | 55.6 (31.72-77.15) | 2.1 | 71.75 (53.89-84.66) | 1.81 | 1.95 (1.48-2.56) |
| Epilepsy | 12 | 10717 | 69 | 3065 | 436 | 7147 | 68.75 (25.8-93.3) | 2.51 | 63.66 (43.54-79.92) | 1.38 | 1.88 (0.99-2.65) |
| Inflammatory bowel disease | 6 | 2886 | 20 | 1163 | 12 | 1691 | 83.37 (36.58-97.76) | 2.09 | 54.03 (45.96-61.89) | 0.38 | 1.8 (0.91-2.05) |
| Systemic lupus erythematosus | 6 | 1004 | 8 | 532 | 1 | 463 | 88.55 (38.07-98.98) | 0.91 | 46.82 (40.33-53.41) | 0.28 | 1.65 (0.74-1.94) |
| Irritable bowel syndrome | 18 | 18446 | 161 | 4962 | 681 | 12642 | 58.34 (35.6-78.01) | 1.88 | 63.29 (50.76-74.25) | 1.11 | 1.58 (1.3-1.77) |
| Subfertility or recurrent pregnancy loss | 16 | 12690 | 185 | 3418 | 623 | 8464 | 67.22 (35.5-88.43) | 2.4 | 57.22 (37.4-74.97) | 1.59 | 1.56 (1.18-1.95) |
| Fracture | 8 | 24741 | 81 | 2179 | 468 | 22013 | 27.47 (8.14-61.83) | 2.02 | 82.13 (54.61-94.61) | 1.93 | 1.53 (0.99-2.31) |
| Arthritis | 15 | 10745 | 67 | 2045 | 475 | 8158 | 64.8 (35.33-86.12) | 1.93 | 55.46 (34.26-74.84) | 1.71 | 1.45 (1-2.05) |
| Type 2 Diabetes | 6 | 8199 | 34 | 972 | 76 | 7117 | 53.01 (14.9-87.91) | 2.11 | 59.72 (35.7-79.83) | 1.22 | 1.31 (0.65-1.72) |
| Multiple sclerosis | 5 | 1086 | 7 | 501 | 5 | 573 | 20.82 (0.46-93.76) | 1.4 | 54.35 (48.71-59.88) | 0.21 | 0.46 (0.01-2.12) |
| **Genetic predisposition** |  |  |  |  |  |  |  |  |  |  |  |
| Family history of CD | 13 | 31827 | 332 | 7021 | 340 | 24134 | 42.37 (6.54-88.53) | 3.96 | 84.56 (57.54-95.68) | 2.45 | 2.7 (1.23-3.88) |
| HLA DQ2/DQ8 | 10 | 19466 | 470 | 7324 | 43 | 11629 | 94.62 (84.39-98.28) | 1.51 | 64.76 (57.27-71.58) | 0.44 | 2.64 (2.23-3.12) |
