# Supplementary material for: The accuracy of diagnostic indicators for coeliac disease: A systematic review and meta-analysis
Source: PLoS One. 2021 Oct 25;16(10):e0258501. doi: 10.1371/journal.pone.0258501 (PMC8545431; doi:10.1371/journal.pone.0258501)
Supplement: S4 Table — (DOCX) [file pone.0258501.s008.docx]

## Table S4: Summary estimates of sensitivity, specificity, and prediction values of subgroup analyses

Meta-analysis results, number of studies, total numbers of true positives (TP), false positives (FP), false negatives (FN), and true negatives (TN) are shown per diagnostic indicator. Tau represents the between-study standard deviation in sensitivity and specificity on the logit scale. *PPVs are calculated using the summary estimates of sensitivity and specificity for the general population assuming a 1% CD prevalence.

| **Diagnostic indicator** | **Subgroup** | **Studies** | **Sample size** | **TP** | **FP** | **FN** | **TN** | **Sensitivity** | **Tau sensitivity** | **Specificity** | **Tau specificity** | **PPV*** |
| --- | --- | --- | --- | --- | --- | --- | --- | --- | --- | --- | --- | --- |
|  |  |  |  |  |  |  |  | **(95% CI)** |  | **(95% CI)** |  | **(95% CI)** |
| **Age group** |  |  |  |  |  |  |  |  |  |  |  |  |
| Abdominal pain | Adults | 6 | 8553 | 107 | 1541 | 489 | 6416 | 0.41 (0.16-0.7) | 1.46 | 0.81 (0.54-0.94) | 1.59 | 2.09 (0.85-5.29) |
| Abdominal pain | Children | 6 | 39898 | 148 | 12804 | 270 | 26676 | 0.43 (0.26-0.62) | 0.90 | 0.65 (0.5-0.78) | 0.78 | 1.22 (1.06-1.38) |
| Arthritis | Adults | 8 | 8816 | 12 | 795 | 465 | 7544 | 0.24 (0.06-0.59) | 1.63 | 0.69 (0.33-0.91) | 2.17 | 0.78 (0.35-1.69) |
| Arthritis | Children | 5 | 855 | 47 | 408 | 7 | 393 | 0.87 (0.74-0.94) | 0.00 | 0.46 (0.37-0.55) | 0.38 | 1.59 (1.28-1.95) |
| Constipation | Adults | 5 | 12705 | 42 | 1805 | 470 | 10388 | 0.16 (0.07-0.34) | 0.99 | 0.91 (0.81-0.96) | 1.00 | 1.79 (0.81-3.83) |
| Constipation | Children | 7 | 41581 | 59 | 5412 | 372 | 35738 | 0.16 (0.11-0.22) | 0.27 | 0.83 (0.77-0.88) | 0.49 | 0.92 (0.66-1.27) |
| Diarrhoea | Adults | 7 | 14273 | 64 | 1960 | 589 | 11660 | 0.1 (0.04-0.24) | 1.19 | 0.93 (0.79-0.98) | 1.68 | 1.43 (0.53-3.96) |
| Diarrhoea | Children | 6 | 41227 | 97 | 4424 | 376 | 36330 | 0.16 (0.03-0.51) | 2.08 | 0.9 (0.75-0.96) | 1.34 | 1.55 (0.8-2.35) |
| Type 1 Diabetes | Adults | 11 | 13839 | 154 | 2016 | 493 | 11176 | 0.65 (0.17-0.94) | 3.53 | 0.83 (0.53-0.95) | 2.44 | 3.68 (1.93-5.64) |
| Type 1 Diabetes | Children | 12 | 6522 | 445 | 2755 | 132 | 3190 | 0.82 (0.7-0.9) | 0.65 | 0.54 (0.42-0.65) | 0.79 | 1.77 (1.38-2.32) |
| Type 1 Diabetes | Mixed | 8 | 6274 | 100 | 1276 | 25 | 4873 | 0.81 (0.72-0.88) | 0.22 | 0.61 (0.48-0.73) | 0.76 | 2.07 (1.56-2.88) |
| **CD diagnosis** |  |  |  |  |  |  |  |  |  |  |  |  |
| Anaemia | Serology only | 9 | 9664 | 93 | 1061 | 547 | 7963 | 0.33 (0.13-0.61) | 1.63 | 0.85 (0.7-0.93) | 1.33 | 2.19 (1.42-3.17) |
| Anaemia | Serology +/- biopsy | 8 | 3813 | 60 | 1285 | 15 | 2453 | 0.88 (0.69-0.96) | 1.27 | 0.6 (0.42-0.76) | 1.05 | 2.16 (1.59-3.08) |
| Arthritis | Serology only | 7 | 9429 | 51 | 1401 | 473 | 7504 | 0.44 (0.11-0.84) | 2.33 | 0.65 (0.23-0.92) | 2.45 | 1.28 (0.78-2.29) |
| Arthritis | Serology +/- biopsy | 8 | 1316 | 16 | 644 | 2 | 654 | 0.89 (0.6-0.98) | 0.06 | 0.48 (0.42-0.54) | 0.30 | 1.7 (1.14-2) |
| Chronic liver disease | Serology only | 7 | 3556 | 26 | 462 | 405 | 2663 | 0.18 (0.01-0.87) | 4.05 | 0.91 (0.64-0.98) | 2.12 | 1.98 (0.37-3.7) |
| Chronic liver disease | Serology +/- biopsy | 8 | 5126 | 12 | 1682 | 5 | 3427 | 0.87 (0.12-1) | 2.11 | 0.59 (0.48-0.69) | 0.61 | 2.08 (0.3-2.8) |
| Epilepsy | Serology only | 5 | 7529 | 46 | 1379 | 435 | 5669 | 0.2 (0.03-0.66) | 2.28 | 0.79 (0.43-0.95) | 1.83 | 0.96 (0.51-1.43) |
| Epilepsy | Serology +/- biopsy | 7 | 3188 | 23 | 1686 | 1 | 1478 | 1 (0-1) | 5.39 | 0.49 (0.43-0.54) | 0.25 | 1.93 (0-2.11) |
| Irritable bowel syndrome | Serology only | 11 | 8192 | 101 | 2408 | 551 | 5132 | 0.56 (0.26-0.83) | 2.07 | 0.62 (0.45-0.76) | 1.15 | 1.47 (1.04-1.71) |
| Irritable bowel syndrome | Serology +/- biopsy | 7 | 10254 | 60 | 2554 | 130 | 7510 | 0.6 (0.3-0.84) | 1.55 | 0.65 (0.47-0.79) | 0.97 | 1.7 (1.29-2.02) |
| Thyroid disease | Serology only | 13 | 17129 | 134 | 2506 | 544 | 13945 | 0.36 (0.13-0.68) | 2.33 | 0.76 (0.5-0.91) | 2.00 | 1.48 (1.2-1.75) |
| Thyroid disease | Serology +/- biopsy | 10 | 9902 | 78 | 2246 | 327 | 7251 | 0.79 (0.48-0.94) | 1.76 | 0.66 (0.42-0.83) | 1.48 | 2.26 (1.53-3.5) |
| Type 1 Diabetes | Serology only | 17 | 15777 | 521 | 3313 | 613 | 11330 | 0.6 (0.29-0.85) | 2.51 | 0.74 (0.51-0.89) | 2.11 | 2.31 (1.57-3.4) |
| Type 1 Diabetes | Serology +/- biopsy | 14 | 10858 | 178 | 2734 | 37 | 7909 | 0.9 (0.9-0.9) | 0.98 | 0.6 (0.59-0.6) | 0.91 | 2.21 (2.19-2.23) |
