# Supplementary material for: The accuracy of diagnostic indicators for coeliac disease: A systematic review and meta-analysis
Source: PLoS One. 2021 Oct 25;16(10):e0258501. doi: 10.1371/journal.pone.0258501 (PMC8545431; doi:10.1371/journal.pone.0258501)
Supplement: S5 Table — (DOCX) [file pone.0258501.s009.docx]

## Table S5: Summary estimates of sensitivity, specificity, and prediction values of sensitivity analyses

Meta-analysis results, number of studies, total numbers of true positives (TP), false positives (FP), false negatives (FN), and true negatives (TN) are shown per diagnostic indicator. Tau represents the between-study standard deviation in sensitivity and specificity on the logit scale. *PPVs are calculated using the summary estimates of sensitivity and specificity for the general population assuming a 1% CD prevalence.

| **Diagnostic indicator** | **Subgroup** | **Studies** | **Sample size** | **TP** | **FP** | **FN** | **TN** | **Sensitivity** | **Tau sensitivity** | **Specificity** | **Tau specificity** | **PPV*** |
| --- | --- | --- | --- | --- | --- | --- | --- | --- | --- | --- | --- | --- |
|  |  |  |  |  |  |  |  | **(95% CI)** |  | **(95% CI)** |  | **(95% CI)** |
|  |  |  |  |  |  |  |  |  |  |  |  |  |
| Abdominal pain | All | 12 | 48451 | 255 | 14345 | 759 | 33092 | 0.39 (0.24-0.57) | 1.15 | 0.74 (0.57-0.85) | 1.31 | 1.47 (0.95-2.33) |
| Abdominal pain | Cohort | 7 | 45897 | 159 | 13548 | 302 | 31888 | 0.35 (0.22-0.5) | 0.78 | 0.79 (0.57-0.92) | 1.44 | 1.67 (0.84-3.73) |
| Anaemia | All | 17 | 13477 | 153 | 2346 | 562 | 10416 | 0.63 (0.37-0.84) | 2.08 | 0.75 (0.61-0.86) | 1.38 | 2.52 (1.94-3.16) |
| Anaemia | Cohort | 6 | 10229 | 45 | 1325 | 73 | 8786 | 0.34 (0.14-0.63) | 1.37 | 0.9 (0.79-0.96) | 1.08 | 3.38 (2.47-4.34) |
| Bloating or abdominal distension | All | 6 | 32694 | 64 | 5809 | 560 | 26261 | 0.18 (0.07-0.4) | 1.28 | 0.83 (0.62-0.94) | 1.36 | 1.09 (0.79-1.52) |
| Bloating or abdominal distension | Cohort | 5 | 31894 | 56 | 5805 | 168 | 25865 | 0.26 (0.14-0.44) | 0.78 | 0.74 (0.61-0.84) | 0.69 | 1.03 (0.72-1.41) |
| Constipation | All | 12 | 54286 | 101 | 7217 | 842 | 46126 | 0.16 (0.1-0.24) | 0.72 | 0.87 (0.8-0.91) | 0.82 | 1.2 (0.83-1.74) |
| Constipation | Cohort | 9 | 51561 | 82 | 6691 | 446 | 44342 | 0.16 (0.12-0.22) | 0.32 | 0.88 (0.82-0.92) | 0.71 | 1.32 (0.83-2.11) |
| Diarrhoea | All | 13 | 55500 | 161 | 6384 | 965 | 47990 | 0.12 (0.05-0.28) | 1.81 | 0.92 (0.82-0.96) | 1.53 | 1.4 (0.76-2.46) |
| Diarrhoea | Cohort | 10 | 52669 | 67 | 5524 | 473 | 46605 | 0.1 (0.04-0.2) | 1.22 | 0.94 (0.85-0.97) | 1.50 | 1.54 (0.82-2.89) |
