# Supplementary material for: The accuracy of diagnostic indicators for coeliac disease: A systematic review and meta-analysis
Source: PLoS One. 2021 Oct 25;16(10):e0258501. doi: 10.1371/journal.pone.0258501 (PMC8545431; doi:10.1371/journal.pone.0258501)
Supplement: Attachment — Submitted filename: Responses to reviewers v2.docx [file pone.0258501.s011.docx]

# Responses to reviewers’ comments

PLOS One

We would like to thank the reviewers for taking the time to review our manuscript. The original search was done in February 2020, so we felt it was necessary to update the search of this systematic review before resubmitting. We identified another 11 relevant studies and updated the analyses, figures, tables, and manuscript accordingly alongside the suggested changes from the reviewers.

## Reviewer #1

I confine my remarks to statistical aspects of this paper. The analysis itself is fine, but I have a couple suggestions about the figures.

Figure 2 - Stacked bar plots are not recommended. A better choice is a Cleveland dot plot. See the work of William S. Cleveland for examples and reasons.

**Authors’ response**

As suggested, we have changed this figure to a Cleveland dot plot, see Figure 2.

Figs. 3, 4, 5 - I would remove the columns of numbers (and put them in a table, if desired), in order to make the graphical part of the figures bigger. Also, please specify whether it is the area or the width of the boxes that indicates the number of participants.

**Authors’ response**

We have removed the sensitivity and specificity estimates from figures 3, 4, and 5. These numbers can now be found in separate tables in the supplementary material as table s4 and table s5 (Fig 3 numbers were already presented in Supplementary Table s3).

The area of the box is proportional to the number of participants in the study. We have edited the Figure 3-5 captions to clarify this.

## Reviewer #2

Elwenspoek; PLOS Medicine: accurate of diagnostic indicators for celiac disease.

ABSTRACT

Delete "rigorous" - that is just a word signalling that you are careful. Everyone thinks they are careful.

**Authors’ response**

We originally used the word rigorous, because we performed the systematic review and meta-analysis according to Cochrane’s guidelines and reporting according to PRISMA, which are internationally regarded as the highest standard for systematic review methodology and reporting. As suggested by the reviewer, we have removed the word ‘rigorous’ and replaced it with a clearer explanation:

Abstract, page 2, line 47: *“International guidance for systematic review methods were followed and the review was registered at PROSPERO (CRD42020170766).”*

Why was the analysis of first-degree relatives a sensitivity analysis?

**Authors’ response**

We identified “family history of coeliac disease” as a diagnostic indicator in the literature. Often papers did not specify whether they only included first degree relatives or any family member. However, when our analysis showed a relationship that was much weaker than expected, we included a post hoc sensitivity analysis on studies clearly specifying that they looked at first degree relatives.

Does the fact that CD is twice as common in a disorder automatically mean that patients with those diseases should be screened for CD?

We believe that a cost effectiveness analysis, taking into account the harms of underdiagnosis of CD and the harm of overtesting und unnecessary follow up testing, is necessary to determine where this threshold needs to be. We are currently working on this and our findings from this review will feed into the economic models. The main aim of this review was to identify all published evidence on potential diagnostic indicators for coeliac disease and to estimate their accuracy in predicting/diagnosing coeliac disease.

***

INTRODUCTION.

It is probably not true that patients with CD have had symptoms for 12 years before diagnosis. The paper you cite is a questionnaire study published almost 15 years back. Please update (ref #3).

**Authors’ response**

As suggested, we have updated this reference with a more recent paper that found a diagnostic delay of 3.5 years for a US cohort without GI symptoms (https://www.amjmed.com/article/S0002-9343(17)30606-X/fulltext). We have changed this sentence in the introduction accordingly:

Introduction, page 4, lines 79-80: “*Many CD patients experience a delay in diagnosis, especially when having non-specific symptoms, which can take several years.[3]*”

Please also delete the citation about untreated celiac disease and infertility. There have been so many studies showing that celiac disease is not linked to infertility. It is a mystery how this misconception survives and makes it into reviews. Also the evidence that undiagnosed CD will push up cancer incidence is weak but I will not argue about that statement.

**Authors’ response**

We have removed the citation about untreated celiac disease and infertility, because, as pointed out by the reviewer, this is not a generally accepted long-term consequence of CD anymore and the most recent systematic review found no increased prevalence of CD in women with infertility (Glimberg et al 2021: <https://www.ncbi.nlm.nih.gov/pmc/articles/PMC8022102/#rmb212374-bib-0017>)

Instead, we refer to pregnancy-related complications, which have been shown to be more common in women with CD compared to women without CD in a meta-analysis (Saconne et al 2016, https://pubmed.ncbi.nlm.nih.gov/26432464/).

Introduction, page 4, lines 80-83: *“If unrecognised and untreated, the accumulating damage in the small intestines impairs nutrient absorption which can lead to osteoporosis and anaemia, and increases the risk of developing pregnancy-related complications in woman and certain types of cancer.[4, 5]”*

It is not clear to me how the following sentence adds to your paper: "During the coronavirus pandemic, interim guidance was published in the UK for a non-biopsy protocol for adults, which may reduce biopsies by a third.[8]"

**Authors’ response**

This was to show that a biopsy avoiding strategy has already been used in practice for adults for practical reasons during the coronavirus pandemic. However, we agree that this is not essential information for this review and have removed it.

Regarding your discussion of screening and whether it fulfils criteria for general screening, please cite: <https://pubmed.ncbi.nlm.nih.gov/25922671/>

**Authors’ response**

We have now included this reference (page 5, line 98).

***

METHODS

Great that you registered your study in PROSPERO.

I had a quick look at the search strategy (your supplement), it looks relevant.

I am impressed by the patient involvement in the study.

I am impressed by your bias assessment, and your discussion around "only serology test", as opposed to "serology tests confirmed though biopsy".

Table 1. In what way are the case-control studies nested? [to me only case-control studies within a cohort are "nested")]

**Authors’ response**

We have applied the same definition of nested case control studies as the reviewer. We have now clarified this definition in the manuscript:

Table 1, Page 13, lines 240-241: *“‘Nested’ case-control studies are nested within a cohort, where cases and controls are selected from the same cohort.”*

***

RESULTS.

In my mind you present the association between Celiac disease and associated conditions in a rather strange way. The positive predictive value for (celiac disease??) in migraine is 2%? You mean the prevalence of celiac disease in migraine patients is 2%??

(Does that mean that you suggest that only 29% of patients with dermatitis herpetiformis have celiac disease??). In Figure 3, please make a comment on the 29% PPV in the figure legend since the 29 value does not show clearly in the figure.

**Authors’ response**

We agree with the reviewer that this is an unusual way of presenting the data, but this is what we set out to do: to treat diagnostic indicators as diagnostic tests, and to calculate the accuracy of each "test” (e.g., Test: does the patient have type 1 diabetes? If ‘yes’ this was treated as a positive test result, if ‘no’ this was treated as a negative test result). The positive predictive value of e.g., migraine is the chance of a migraine patient having CD, in a population with a 1% CD prevalence. 1% is believed to be the CD prevalence of the general population.

The summary estimate for dermatitis herpetiformis of 29% is very imprecise, with 95% confidence interval 3% to 72%. This suggests that DH patients may be between 3 and 72 times more likely to have CD than a randomly selected individual from the general population.

We highlight this in the text as follows (results):

Results, page 17, lines 271-273: *“Amongst risk conditions, dermatitis herpetiformis had the highest estimated sensitivity, specificity, and PPV (estimated PPV at 1% prevalence of CD = 29%, 95% CIs 3 to 72%). However, the uncertainty around these estimates was substantial, as is shown by the wide 95% CIs.”*

Just a note, I am extremely surprised you did not find an increase likelihood of CD in patients with psoriasis, chronic liver disease, or SLE.

In your revised discussion I'd like you to discuss these three conditions and why you didn't find an association with CD? The null findings here go strictly against my clinical and scientific impression (disclosure: I may be wrong…).

**Authors’ response**

We did estimate increased risks of CD in patients with psoriasis, chronic liver disease, and SLE: we estimated 1.95% (0.9-2.2%) risk of CD in patients with psoriasis, 2.2% (1.3-2.9%) in patients with chronic liver disease, and 1.7% (0.7 – 1.9%) in patients with SLE, among a general population with 1% CD prevalence. Notably the evidence for an association between CD and chronic liver disease is a little stronger in our updated review, following the update search: we now find statistical evidence that the risk of CD among chronic liver disease patients is higher than the general population. However, the estimated PPVs for SLE and psoriasis remain imprecise, with 95% CIs crossing the population prevalence level of 1%, such that we cannot be confident about a true increase in risk in these populations.

We agree with the reviewer that we worded this too strongly in the results and we have reformulated this to reflect the uncertainty of our estimates:

Results, page 17, lines 280-285: *“Although the estimated PPVs of psoriasis, epilepsy, inflammatory bowel disease, systematic lupus erythematosus, fracture, arthritis, and type 2 diabetes suggest an increased likelihood of CD in people with these conditions, there was considerable uncertainty in these estimates. The 95% CIs crossed or touched the line of population prevalence, indicating that the likelihood of CD may be similar to that in the general population. We found no evidence of an increased likelihood of CD in people with multiple sclerosis (Figure 3).”*

Also, I am very surprised that people with a family history of CD were not more likely to have CD than the general population. This goes very much against the high heredity of celiac disease (e.g. Kuja-Halkola R, Lebwohl B, Halfvarson J, et al. Heritability of non-HLA genetics in coeliac disease: a population-based study in 107 000 twins. Gut 2016;65:1793-1798), please also cite the Kuja-Halkola paper.

I note however that when you restrict your analysis to 1st_degree relatives the PPV is rather positive, 3% but not stat significant. And I note that you have commented on this in the abstract which is good.

**Authors’ response**

We have included the reference to this study in the discussion:

Discussion, page 20, lines 411-413: *“Although almost 100% of individuals with CD are carriers of the HLA DQ2/DQ8 risk alleles, they only account for a small proportion of the heritability of CD.”*

Also the association with sub fertility is surprising. Recent data do not suggest an association here…

1.	Dhalwani NN, West J, Sultan AA, et al. Women with celiac disease present with fertility problems no more often than women in the general population. Gastroenterology 2014;147:1267-74 e1; quiz e13-4.

2.	Tata LJ, Card TR, Logan RF, et al. Fertility and pregnancy-related events in women with celiac disease: a population-based cohort study. Gastroenterology 2005;128:849-55.

3.	Zugna D, Richiardi L, Akre O, et al. A nationwide population-based study to determine whether coeliac disease is associated with infertility. Gut 2010;59:1471-5.

4.	Hogen Esch CE, Van Rijssen MJ, Roos A, et al. Screening for unrecognized coeliac disease in subfertile couples. Scand J Gastroenterol 2011;46:1423-8.

**Authors’ response**

We would like to thank the reviewer for bringing this to our attention. We have included an extra paragraph in the discussion to address these discrepancies.

Discussion, page 22, lines 397-408: *“Recent data have suggested that infertility and CD are not associated, although most guidelines still name infertility as one of the risk factors for CD. Also recent meta-analyses have shown conflicting results regarding the association between infertility and CD. Singh et al. (2016), Castaño et al. (2019), and Glimberg et al. (2021) reported a pooled prevalence of biopsy‐proven CD in women with infertility between 1.4‐3.5%, 0.6‐2.8%, and 0.2‐1.2%, respectively. Glimberg et al. meta-analysed 11 studies and found a prevalence of CD among women with infertility similar to that of the general population. Our results also suggest that women with subfertility have a 1.2 to 2-fold higher risk of CD. We used a broader definition of subfertility including recurrent pregnancy loss, whereas pregnancy loss was excluded by Glimberg et al., and we only included studies with control groups, which may explain the discrepancies in our and Glimberg’s findings. Large scale prospective cohort studies, such as birth cohorts, are needed to address this question, where all participants are tested for CD and information on infertility is collected.”*

DISCUSSION

How can subgroup analyses rule out over- or underestimation? (line 345)?

**Authors’ response**

Our subgroup and sensitivity analyses cannot rule out over- or underestimation, but they allowed us to investigate known causes of over- and underestimation, namely study design and imperfect reference standards (see Lijmer et al., 1999. https://pubmed.ncbi.nlm.nih.gov/10493205/). These were also the main cause for judging studies at a high risk of bias, so we investigated their impact. The use of serological tests to diagnose CD without a confirmatory biopsy can lead to an underestimation of the association between CD and a diagnostic indicator, because the effect may be diluted by the inclusion of false positives (patients that would have been excluded if a confirmation biopsy had been performed).

Case control designs can lead to an overestimation of the association, because difficult-to-diagnose patients or borderline positives/negatives are usually not represented in a case control (because they did not fulfil the inclusion criteria for the case nor the control group, but they are present in normal clinical practice and in cohort studies).

However, we did not see significant differences in the pooled estimates if we left out case control studies, or if we compared studies that used a confirmation biopsy to studies that only used serology tests.

Since the association with migraine is one of your main findings, I suggest you discuss your findings in relation to one of the largest studies on CD and migraine: <https://pubmed.ncbi.nlm.nih.gov/26972425/>	[HR=1.67]

**Authors’ response**

We have added this reference to our discussion and added this sentence:

Discussion, page 22, lines 394-396: “*A population-based retrospective cohort study reported that among 160,000 patients headache-related visits, including migraine, occurred 1.6 to 1.8 times more frequently in CD patients than in controls.[44]”*

line 360, typo?

.	cannot be used to estimate how predictive CD (??) these indicators are in combination

**Authors’ response**

This was indeed a typo, now rectified:

Discussion, page 21, line 377-378: *“…cannot be used to estimate how predictive these indicators are in combination…”*
